# Expression alterations define unique molecular characteristics of spinal ependymomas

**DOI:** 10.18632/oncotarget.3715

**Published:** 2015-03-30

**Authors:** Anbarasu Lourdusamy, Ruman Rahman, Richard G. Grundy

**Affiliations:** ^1^ Children's Brain Tumour Research Centre, School of Medicine, Queen's Medical Centre University of Nottingham, Nottingham, UK

**Keywords:** ependymoma, gene expression, meta-analysis, co-expression network

## Abstract

Ependymomas are glial tumors that originate in either intracranial or spinal regions. Although tumors from different regions are histologically similar, they are biologically distinct. We therefore sought to identify molecular characteristics of spinal ependymomas (SEPN) in order to better understand the disease biology of these tumors. Using gene expression profiles of 256 tumor samples, we identified increased expression of 1,866 genes in SEPN when compared to intracranial ependymomas. These genes are mainly related to anterior/posterior pattern specification, response to oxidative stress, glial cell differentiation, DNA repair, and PPAR signalling, and also significantly enriched with cellular senescence genes (P = 5.5 × 10^−03^). In addition, a high number of significantly down-regulated genes in SEPN are localized to chromosome 22 (81 genes from chr22: 43,325,255 – 135,720,974; FDR = 1.77 × 10^−23^ and 22 genes from chr22: 324,739 – 32,822,302; FDR = 2.07 × 10^−09^) including *BRD1, EP300, HDAC10, HIRA, HIC2, MKL1*, and *NF2*. Evaluation of *NF2* co-expressed genes further confirms the enrichment of chromosome 22 regions. Finally, systematic integration of chromosome 22 genes with interactome and *NF2* co-expression data identifies key candidate genes. Our results reveal unique molecular characteristics of SEPN such as altered expression of cellular senescence and chromosome 22 genes.

## INTRODUCTION

Ependymomas are primary tumors of the central nervous system (CNS) representing 3%−6% of all CNS tumor [1]. Histologically, they have been classified into three grades according to the World Health Organization (WHO): grade I (subependymomas and myxopapillary ependymomas), grade II (classic ependymomas), and grade III (anaplastic ependymomas) [2]. These tumors originate from either intracranial or spinal regions of the CNS. Spinal ependymoma (SEPN) constitutes approximately 34.5% of ependymomas and accounts for 60% spinal cord gliomas, making them the most common glial tumors of the adult spine [3-7]. While 90% of all childhood ependymomas are intracranial, SEPN are more commonly found in adults of 20 to 40 years of age [7] where standard treatment of SEPN is aiming for complete resection. Although, SEPN generally have better prognosis than intracranial tumors, recurrence rate can be as high as 50%−70% without adjuvant therapy; however the beneficial role of adjuvant chemotherapy or radiotherapy in SEPN is controversial [4, 7]. Most currently known prognostic factors for SEPN are based on clinical and histological criteria, such as extent of tumor resection, and histological grade. The results of existing studies on these prognostic markers are contradictory. Therefore, there is a need to improve the understanding of the biology of SEPN in order to develop more accurate prognostic signatures and identify new therapeutic targets.

Few studies have examined the genetics of SEPN compared to intracranial ependymomas. Ebert *et al* found loss of heterozygosity (LOH) on chromosome 22q in grade II (6/14 cases) and grade III (1/3 case) SEPN, NF2 mutations in grade II SEPN (6/14 cases), and found no mutations in intracranial and myxopapillary ependymomas [8]. In a study with 52 tumors from 45 patients, Lamsuzs *et al* detected LOH on chr22q more frequently in intramedullary SEPN (14/20 cases) compared to intracranial ependymomas (6/25 cases) and found NF2 mutations in 5 out 20 cases of SEPN [9]. Singh *et al* found the NF2 gene deletion in 5/15 SEPN and loss of the NF2 gene product, merlin in 5 out of 27 cases, all of which were from spinal [10].

Microarray-based expression studies have also been used to compare SEPN with intracranial tumors and correlate molecular signatures with clinical and histologic characteristics. A study by Korshunov *et al* examined 39 ependymal neoplasms including ten SEPN and detected 14 genes that were more highly expressed in SEPN compared to intracranial ependymomas including *HOXB5, PLA2G5* and *ITIH2* [11]. Lukashova-v Zangen *et al* reported five genes (*TFAM, EDN1, GAS2L1, HUMRTVLH3* and *ADAM9*) that were preferentially expressed in SEPN by comparing grade II adult SEPN (n = 8) and adult intracranial ependymomas (n = 4) from a cohort of 47 ependymoma patients [12]. By comparing tumor samples from 16 SEPN and 16 intracranial ependymomas, Palm *et al* reported the over-expression of HOX genes in SEPN and up-regulation of several genes of Notch, Hedgehog, and BMP signaling pathways in intracranial ependymoma [13]. Finally, Taylor *et al* compared gene expression microarrays of tumor samples from SEPN (n = 3), supratentorial (n = 5), and posterior fossa (n = 21), and identified the expression signature for SEPN that consisted of 184 genes including HOX genes *HOXA7, A9, HOXB6, B7, HOXC8*, *C10*, and *IGF1* [14].

The aforementioned gene expression studies are limited to a small number of samples and are usually analysed in isolation. Meta-analysis approaches make it possible to combine multiple independent gene expression datasets and increase the statistical power for gene discovery. Such meta-analysis approaches have been successfully used to identify transcriptional signatures in cancer [15] and aging [16]. Individual gene expression studies are limited by systematic biases at both biological and technical levels, which hinder the broader application of their findings. Meta-analysis can control for such confounding factors by increasing the statistical power to detect consistent changes across multiple datasets. No systematic multi-cohort analysis has yet evaluated transcriptional alterations between SEPN and intracranial ependymomas. The present study uses microarray datasets from three independent cohorts to compare the biology of SEPN and intracranial ependymomas to identify unique molecular characteristics of SEPN such as altered expression of cellular senescence and chromosome 22 genes.

## RESULTS

We applied two different meta-analysis approaches to the normalized expression data from three independent studies to find differentially expressed genes. A total of 3,182 genes were identified as significantly differentially expressed (FDR < 0.05) between SEPN and intracranial ependymomas by both methods (Supplementary Table 1).

### Expression analysis highlights diverse processes and pathways among over-expressed genes in SEPN

**Figure 1:**
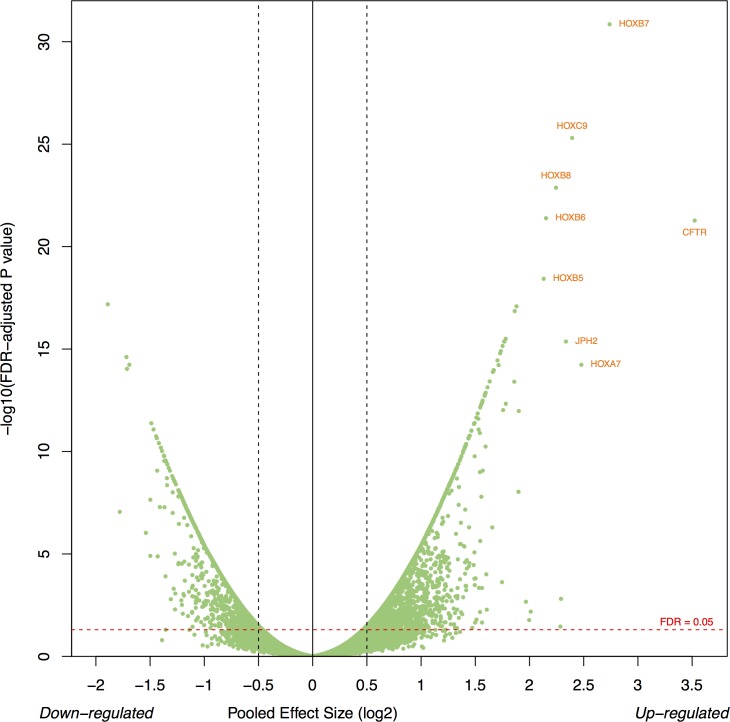

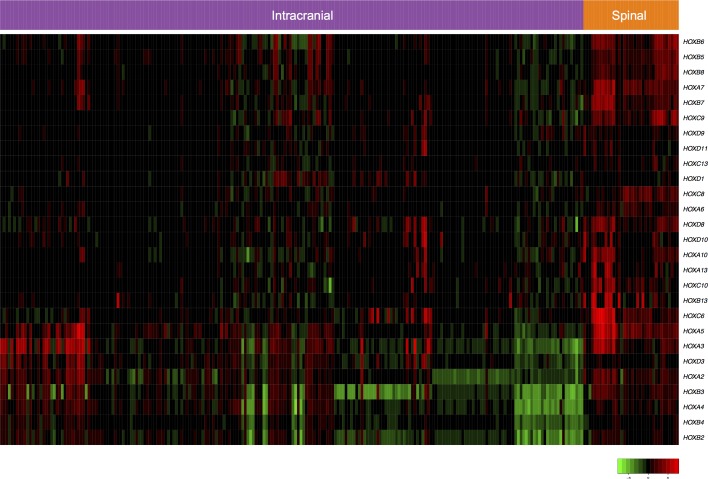
Gene expression profiles define unique molecular characteristics of SEPN **A**. Volcano plot showing the number of significantly differentially expressed genes in SEPN (FDR < 0.05). The x-axis represents the pooled effect sizes that are estimated from three independent microarray datasets by the meta-analysis of random-effects model and the y-axis represents –log10 of false discovery rate (FDR). **B**. Heatmap depicting expression profiles of HOX gene that are significantly enriched in SEPN. The normalized value of each gene is indicated by colour intensity, with red/green representing high/low expression.

Of the 3,182 differentially expressed genes between SEPN and intracranial ependymomas, 59% (1866) were consistently up-regulated in SEPN (Figure 1A). The most significantly up-regulated genes included *HOXB7* (pooled effect size on log2, ES = 2.74, FDR = 1.41 × 10^−31^), *CTFR* (ES = 3.52, FDR = 5.33 × 10^−22^), *HOXB5* (ES = 2.13, FDR = 3.72 × 10^−19^), and *CTTNBP2* (ES = 1.77, FDR = 4.29 × 10^−16^). Other interesting genes that have increased expression in SEPN included *EZH1, IDH3, NEFL*, and *NELL2*. Of the 27 HOX genes annotated in the microarrays, 22 were significantly up-regulated in the SEPN (Figure 1B). Gene ontology (GO) analysis of over-expressed genes showed significant overrepresentation of genes involved in anterior/posterior pattern specification (27 genes; FDR = 9.37 × 10^−10^), apoptotic process (72 genes; FDR = 6.70 × 10^−08^), cell cycle (52 genes; FDR = 1.68 × 10^−05^), cilium assembly (13 genes; FDR = 5.64 × 10^−05^), and cell proliferation (35 genes; FDR = 2.62 × 10^−03^), as well as genes involved protein folding, DNA replication, mitochondrial electron transport, NADH to ubiquinone, response to oxidative stress, and cell redox homeostasis (Supplementary Table 2). In addition, genes involved in DNA repair processes such as nucleotide-excision repair and double-strand break; genes in signaling pathways such as positive regulation of I-kappaB kinase/NF-kappaB cascade, positive regulation of MAPK cascade; and glial cell differentiation were significantly enriched in SEPN (Supplementary Table 2). Our pathway analysis identified Protein processing in endoplasmic reticulum as the top canonical pathway followed by pathways involved in DNA repair, cell adhesion, metabolism, and PPAR signaling (Table 1).

**Table 1:** Enrichment of KEGG pathways among significantly up-regulated genes in spinal ependymomas

Pathway	N	FDR
Protein processing in endoplasmic reticulum	28	5.44 × 10^−06^
Tight junction	21	4.06 × 10^−04^
Nucleotide excision repair	10	1.98 × 10^−03^
Cell adhesion molecules (CAMs)	19	2.01 × 10^−03^
Pyrimidine metabolism	15	3.25 × 10^−03^
Pyruvate metabolism	9	4.17 × 10^−03^
RNA transport	19	6.02 × 10^−03^
Purine metabolism	20	6.11 × 10^−03^
PPAR signaling pathway	11	1.69 × 10^−02^
Proteasome	8	1.70 × 10^−02^
Adherens junction	11	1.79 × 10^−02^
Oxidative phosphorylation	16	2.04 × 10^−02^
Focal adhesion	21	2.54 × 10^−02^

Enrichment P values for KEGG pathways were calculated using the Hyper-geometric test and corrected for multiple testing using Benjamini & Hoachberg method. The pathways with adjusted P values < 0.05 were selected. N: number of genes in the pathway; FDR: false discovery rate.

### Cellular senescence genes are over-expressed in SEPN

**Figure 2:**
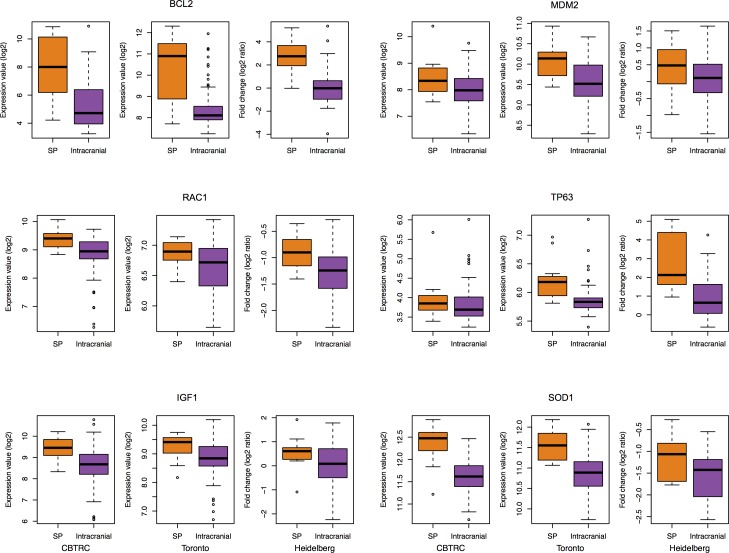
Increased expression of cellular senescence genes in SEPN Box plots showing log_2_ expression levels (y-axis) of six cellular senescence genes (*BCL2*, *MDM2*, *RAC1*, *TP63*, *IGF1,* and *SOD1*) from spinal (SP) and intracranial ependymomas. Expression values from three different studies: CBTRC, Toronto, and Heidelberg are displayed for each gene.

Cancer incidence and mortality increases with age and thus age is considered as a prime risk factor for several types of cancers, including gliomas [17, 18]. In addition, emerging evidences indicate that aging and cancer are closely related and mechanisms underlying the cellular senescence (CS) program may link these two processes [19]. To determine whether similar processes are shared between SEPN and CS, we compared the genes associated with CS with up-regulated genes in SEPN. We obtained a set of 261 candidate genes that are involved in CS from the published study [20]. The CS list is a manually curated set of candidate genes implicated by genetic or RNA interference (RNAi) interventions (gene knockout, partial or full loss-of-function mutations, RNAi-induced gene silencing, overexpression), which reportedly cause cells to induce, inhibit or reverse CS, and genes shown to be markers of CS. Among the up-regulated genes in SPEN there was a significant enrichment for the CS-associated genes (hypergeometric test, 34 genes; P = 5.5 × 10^−03^), which includes oncogenes (*BCL2, CDK6, MDM2*, and *NR4A3*), cytokines and growth factors (*AGT, CYR61, FGF1*, and *IGF1*), transcription factors (*MECP2, PCGF2, PER2*, and *TP63*), member of RAS superfamily of small GTP-binding proteins (*RAC1*), and genes involved in the oxidative stress pathway (*SOD1*) (Figure 2 and Supplementary Table 3).

### Chromosome 22 genes are under-expressed in SEPN

**Figure 3:**
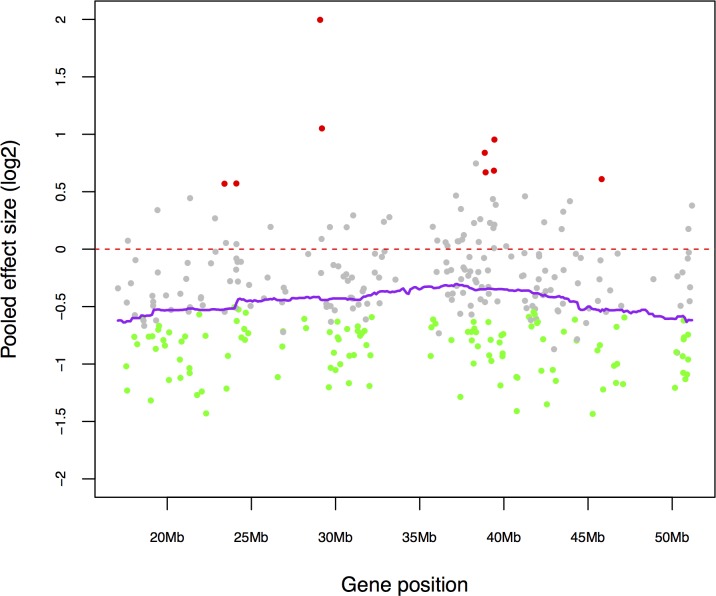

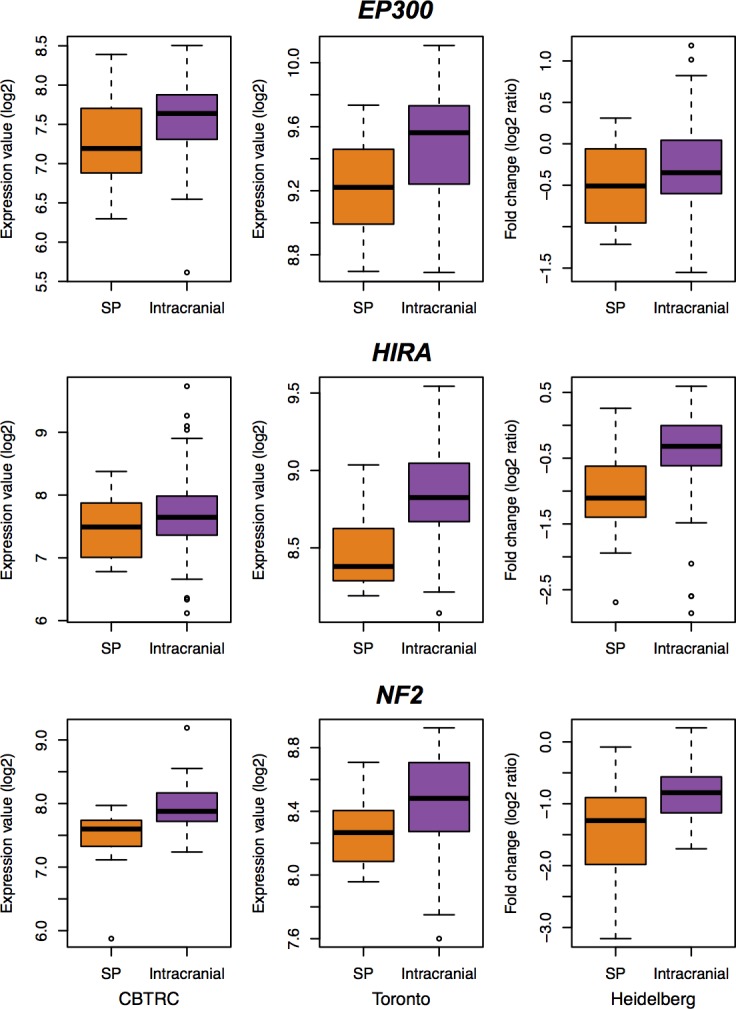
Down-regulated genes of SEPN contributed to the enrichment of chromosome 22 **A**. Expression levels (log_2_) of chromosome 22 genes that are detected in our study. Genes are sorted according to their position on the chromosome (x-axis) and coloured according to the statistical significance of their expression level (y-axis). Significantly down-regulated genes in SEPN (FDR < 0.05) are marked with green, up-regulated ones with red, and non-significant ones with grey. Normalized log_2_ expression values (dots) and kernel-smoothed expression values (purple line) are shown. **B**. Box plots showing log_2_ expression levels (y-axis) of key genes that are located on chromosome (*EP300, HIRA,* and *NF2*) from spinal (SP) and intracranial ependymomas. Expression values from three different studies: CBTRC, Toronto, and Heidelberg are displayed for each gene.

One of the most frequent genomic alterations detected in sporadic ependymomas was the loss of chromosome 22, with the frequency ranging from 26% to 71% [8, 9]. In addition, loss of heterozygosity (LOH) on chromosome 22q has been found more frequently in intramedullary SEPN and more often in adults than the pediatric ones [21, 22]. To investigate whether chromosome loss alter expression of endogenous genes, we identified differentially expressed genes that are significantly down regulated in SEPN (1316 genes at FDR < 0.05, Supplementary Table 1) and tested for the enrichment of chromosomal regions. The positional gene enrichment analysis revealed that many down-regulated genes in SEPN localized to specific chromosomal regions rather than genes randomly distributed in the genome. Most prominently, a high number of genes were localized to a 92.4 Mbp region (81 genes from chr22: 43325255 – 135720974; FDR = 1.77 × 10^−23^) and a 32.5 Mbp region (22 genes from chr22: 324739 – 32822302; FDR = 2.07 × 10^−09^) of the chromosome 22 (Supplementary Table 4). To confirm these observations, we also performed enrichment analysis of cytogenetic bands with the GSEA method. We observed that 51 genes located in chr22q13 (hypergeometric test, FDR = 1.41 × 10^−27^), 27 genes in chr22q12 (FDR = 5.88 × 10^−13^), and 40 genes in chr22q11 (FDR = 4.54 × 10^−11^) were highly enriched among genes that were significantly down-regulated in SEPN (Supplementary Table 4). Furthermore, genes located in cytogenetic bands chr19p13 (59 genes; FDR = 5.88 × 10^−13^), chr19q13 (59 genes; FDR = 1.34 × 10^−09^), and chr20q13 (25 genes; FDR = 2.01 × 10^−06^) were also enriched among down-regulated genes (Supplementary Table 4). Strikingly, 84% of genes located on chromosome 22 (271 out of 321 chr22 genes that were detected in the meta-analysis) were under expressed and 125 of them were significantly down-regulated in spinal when compared to intracranial ependymomas (Figure 3A). These included several cancer-associated genes such as *MKL1, EP300, NF2*, and *HIC2* and chromatin binding genes *BRD1*, *HIRA*, and *HDAC10* (Figure 3B).

### NF2-associated genes in ependymoma are located on chromosome 22

Our analysis of differential expression in relation to chromosome 22 identified a tumor suppressor gene, neurofibromin 2 (*NF2*), which is located at chromosome 22q12.2 and showed decreased expression in SEPN (ES = −1.05, FDR = 8.75 × 10^−04^). Higher incidence of spinal ependymomas in patients with neurofibromatosis type 2 and frequent of loss of the *NF2* gene in SEPN have been reported in numerous studies [23, 24]. This led us to further investigate the effect of *NF2* on the expression of other SEPN genes. We used three independent microarray datasets to identify potential co-expressed genes of *NF2* in an un-biased manner. The meta-analysis of correlations between *NF2* gene expression and other genes resulted in 260 genes (Z mean of correlations, r > 0.4 and FDR < 0.05) (Supplementary Table 5). The most highly correlated gene with *NF2*-expression, the gene encoding the mitochondrial membrane protein *MIEF1*, was significantly down-regulated in SPEN (ES = −1.17, FDR = 4.68 × 10^−08^) (Table 2). Strikingly, enrichment analysis for the cytogenetic band revealed the marked enrichments in the *NF2* co-expressed genes residing at 22q13, 22q12, 22q11, 22q, 14q23, 14q24, and 1q25 (Supplementary Table 4). From a total of 260 *NF2* co-expressed genes, 148 (57%) were significantly down-regulated in SEPN (Supplementary Table 4). Out of fifteen genes that are physically located close to *NF2* (flanking region of 500 Kb), fourteen were down-regulated in SEPN and nine of them were significantly correlated with *NF2* gene expression (Supplementary Table 5).

**Table 2:** Top 25 genes that are highly correlated with NF2 gene expression

Gene	Location	ES	SE	FDR
*MIEF1*	22q13	0.91	0.09	8.34 × 10^−19^
*NDUFA6*	22q13.2	0.73	0.12	4.54 × 10^−08^
*ASCC2*	22q12.1	0.73	0.20	1.78 × 10^−03^
*ADSL*	22q13.2	0.69	0.17	2.66 × 10^−04^
*PITPNB*	22q12.1	0.68	0.08	5.06 × 10^−14^
*RPS19BP1*	22q13.1	0.68	0.09	7.94 × 10^−12^
*UFD1L*	22q11.21	0.64	0.10	9.85 × 10^−09^
*MTMR3*	22q12.2	0.63	0.15	1.69 × 10^−04^
*TCF20*	22q13.3	0.61	0.06	1.58 × 10^−18^
*DRG1*	22q12.2	0.61	0.13	5.14 × 10^−05^
*DGCR2*	22q11.21	0.61	0.08	1.20 × 10^−11^
*EIF3D*	22q13.1	0.61	0.16	1.20 × 10^−03^
*SAMM50*	22q13.31	0.61	0.09	1.24 × 10^−09^
*SRRD*	22q12.1	0.61	0.19	5.65 × 10^−03^
*ITPK1*	14q31	0.60	0.06	6.35 × 10^−18^
*EIF4ENIF1*	22q11.2	0.60	0.18	3.37 × 10^−03^
*MTHFD1*	14q24	0.60	0.08	2.20 × 10^−10^
*TTC38*	22q13	0.60	0.06	1.46 × 10^−17^
*UBE2L3*	22q11.21	0.59	0.16	1.13 × 10^−03^
*THOC5*	22q12.2	0.58	0.17	1.96 × 10^−03^
*TTLL12*	22q13.31	0.58	0.06	6.14 × 10^−17^
*EIF3L*	22q	0.58	0.08	3.51 × 10^−10^
*ATXN10*	22q13.31	0.58	0.24	3.19 × 10^−02^
*FCHSD2*	11q13.4	0.57	0.09	4.51 × 10^−09^
*NOL12*	22q13.1	0.56	0.06	9.44 × 10^−16^

Pearson correlation coefficients (r) between each gene expression and *NF2* expression were calculated in each data set and combined using the DerSimonian-Laird random-effect meta-analytical approach by calculating Z-mean of r (ES) and its standard error (SE). Location: chromosome location; ES: Effect size; SE: standard error; FDR: false discovery rate.

### Prioritisation of chromosome 22 genes by integration of the interactome with NF2-associated genes

We next reasoned that altered expression of chromosome 22 genes could help to identify potential candidate genes of ependymoma in addition to a *NF2* gene. To systematically evaluate and prioritize genes located on chromosome 22 that are associated with SEPN, we used the integration of protein-protein interaction (PPI) data with gene expression data. We first constructed the PPI network using significantly down regulated genes in SEPN that are located on chromosome 22 as seed genes excluding *NF2*. We found there were 11 of 125 chromosome 22 genes participating in the direct network (Supplementary Figure 1). The changes in connectivity in the inferred network were calculated by comparing random networks of equal size and seed genes were ranked by P-value of increased connectivity. This analysis identified eight genes that were ranked by the network algorithm with a P value < 0.05 (Table 3). These eight candidate genes represent the most highly connected, and therefore, potentially most functionally important ones. In order to investigate the relevance of these genes with the known candidate gene, we combine the results of *NF2* gene co-expression analysis with the P-value ranking of the network analysis. All candidate genes except the *MN1* were significantly correlated with the expression profile of *NF2* (Table 3).

**Table 3:** Prioritization of Chromosome 22 genes by network and *NF2* gene co-expression analyses

				Correlation with NF2 gene expression		

Symbol	Gene name	Location	DAPPLE P value	ES	SE	FDR
*EP300*	E1A binding protein p300	22q13.2	0.002	0.45	0.19	0.039
*HIRA*	Histone cell cycle regulator	22q11.21	0.015	0.46	0.14	0.004
*MN1*	Meningioma (disrupted in balanced translocation) 1	22q12.1	0.017	0.10	0.08	0.223
*SGSM3*	Small G protein signaling modulator 3	22q13.1-q13.2	0.021	0.54	0.06	1.04 × 10^−14^
*SUSD2*	Sushi domain containing 2	22q11-q12	0.023	0.21	0.06	0.004
*SREBF2*	Sterol regulatory element binding transcription factor 2	22q13	0.025	0.52	0.06	1.20 × 10^−13^
*RASD2*	RASD family, member 2	22q13.1	0.033	0.26	0.07	5.56 × 10^−04^
*LZTR1*	Leucine-zipper-like transcription regulator 1	22q11.21	0.044	0.42	0.07	1.59 × 10^−07^

## DISCUSSION

Understanding distinct molecular characteristics exhibited by ependymomas according to their tumor location in the brain is becoming more important. Systematic analysis of molecular data from ependymomas have long been sought, however, there currently exists few studies that compared ependymomas arising in the spine to those intracranial. Here we demonstrate through meta-analysis approaches by combining multiple independent data sets that gene expression profiles of tumors from the spine display distinct patterns when compared with tumors from intracranial regions. To our knowledge, this is the first study that provides a comprehensive genome-wide gene expression profile and integrative analysis of SEPN. Most, but not all, biological processes involved in the hallmarks of cancer are enriched with over-expressed genes in SEPN. Genes related to cellular senescence are also highly enriched in SEPN. In addition, we find that the majority of the genes from chromosomes 22 relatively decreased expression levels in tumors from the spine. Overall, we provide a systematic analysis of comprehensive gene expression data for assessing specific biological processes of SEPN.

Our results showed that SEPN are characterized by diverse a set of known and novel biological processes and pathways. Previous work that used smaller study samples reported up-regulation of HOX genes in SEPN when compared with ependymomas from intracranial regions [13, 14]. In the current study, we identified the up-regulation of multiple homeobox family members that include ANTP class homeoboxes (HOX) not previously implicated in SEPN (*HOXA: A2, A3, A5*, and *A10*; *HOXB: B2, B3, B5*, and *B8*; *HOXC: C8* and *C13*; *HOXD: D8, D9*, and *D10*, see Figure 1). The HOX genes, encode a family of evolutionarily conserved transcription factors that have fundamental roles in specifying anterior-posterior body patterning and development of the spine [25]. As they are involved in cellular fate determination and stem cell renewal, several studies investigated their role in other tumor types [26]. Particularly, HOX genes group 10 - 13 play important roles in the normal development of the lumbosacral region [25]. Indeed, the most significant GO biological process detected in this study was the anterior/posterior pattern specification. The combined analysis of large samples in our study from heterogeneous ependymoma patients recruited from different clinical settings provides confirmatory evidence of the association of HOX genes in SEPN. Another important observation is the enrichment of genes involved in glial cell differentiation (*DNER, ERBB2, IGF1, METRN*, and *NFIB*) in SEPN. This observation is consistent with the emerging evidence that radial-glial cells are likely cells of origin for ependymomas [14]. The up-regulation of these glial cell differentiation genes and homeobox family of transcription factors may transform radial-glial cells into cancer stem cells in the development of SEPN. Our analysis also revealed novel biological processes such as antigen processing and presentation of antigen peptides via MHC class I, positive regulation of the IκB kinase/NF-κB cascade, positive regulation of the MAPK cascade, and proteolysis that have genes with relatively increased expression in SEPN (Supplementary Table 1). These results provide data-driven hypotheses for future work, although we expect that further experimental evaluation will be necessary to understand the potential role of these biological processes in SEPN.

Among the up-regulated genes in SEPN were those that regulate DNA repair, DNA damage response, signal transduction by p53, response to oxidative stress, cell cycle, cell division, cell redox homeostasis, and mitochondrial electron transport (NADH to ubiquinone) (Supplementary Table 1). These genetic systems collectively play important roles in aging [27]. Several lines of evidence indicate that CS is a common process that links cancer and aging [19]. Senescent cells accumulate in ageing tissues, which may be due to an increased senescence rate and/or decrease in the rate of clearance of senescent cells. The onset of CS in tumor cells can typically be activated by aberrant activation of oncogenes or loss-of-function of tumor suppressor genes, and also by several stressors, including DNA damage, oxidative stress, and signaling through either MAPK or IGF [19]. To determine a degree of CS convergence with SEPN, we compared the CS-associated genes with genes that are up-regulated in SEPN. Our analysis uncovered evidences of significant overlap between CS and SEPN at a molecular level, identifying core biological processes and genes they share. For example, oncogenes: *CDK6* and *MDM2*, tumor suppressor gene: *CHEK2*, oxidative stress genes: *SOD1* (encoding a member of the p53 family of transcription factors) and *TP63*, and IGF signaling genes: *IGF1, IGFBP5* and *IGFBP7*. Since the incidence of ependymomas from the spine increases with age, it is likely that the senescence pathway is involved in its development. Together, these findings add to the growing body of evidence that CS links cancer and ageing and that biological process in SEPN have a considerable degree of convergence with CS.

Loss of chromosome 22 and LOH have been frequently found in sporadic SEPN, and alteration of chromosome 22q is observed in 40% of SEPN [8, 13]. Analysis of gene expression profiles in the current study revealed the presence of many genes on chromosome 22 that were down-regulated in SEPN, indicating the functional consequences of chromosome 22 loss. The observed 92.4 and 32.5 Mb large domains of repressed transcription of chromosome 22 that include ‘hotspots’ regions 22q11, 22q12, and 22q13 is a result of a gene dosage effect due to unbalanced chromosomal alterations in SEPN. Potential tumor suppressor genes located within 22q12 include *NF2*, which exhibits markedly reduced mRNA expression in SEPN. The *NF2* gene encodes the FERM domain protein *Merlin*, which is co-ordinately regulated by intercellular adhesion and attachment to the extracellular matrix [28]. Increased incidence of CNS tumors including schwannoma, meningioma, and ependymoma has been reported in Neurofibromatosis type 2 patients who carry a single mutated *NF2* allele [23, 24]. The reduced expression of the tumor suppressor gene *NF2* in SEPN raises the question of the significance of expression alterations in other genes. From our meta-analysis of correlations with *NF2* gene expression, it is likely that similar expression alterations may occur in genes located in close physical proximity to *NF2*. Interestingly, genes located on 22q13, 22q12, 22q11 are significantly enriched among *NF2* co-expressed genes. Taken together these results further emphasize the loss of chromosome 22 in the transcriptional regulation of SEPN, and confirm the already reported importance of *NF2* transcriptional down-regulation.

The observed gene expression alterations over large regions of chromosome 22 suggest that these regions may harbour potential candidate genes commonly affected in ependymomas. To prioritize 125 significantly down-regulated chromosome 22 genes in SEPN, we used the protein-protein interactions (PPI) network analysis with the assumption that proteins associated with disease tend to directly interact with each other. We thus identified 19 directly interacting proteins and investigated whether these PPI could help identify candidate genes within any of the 125 chromosome 22 genes identified in the current study. Our analysis identified eight genes, *EP300, HIRA, MN1, SGSM3, SUSD2, SREBF2, RASD2*, and *LZTR1,* and all of them except *MN1* are significantly correlated with *NF2* gene expression. These convergent lines of evidence strongly suggest that the prioritized genes may have pivotal role in ependymomas susceptibility. The tumor suppressor gene, *EP300* ranked highest among all eight genes, encodes histone acetyltransferase, *p300* that have widespread genomic effects on chromatin structure and gene expression as well as non-genomic effects on protein function [29]. It participates in the regulation of a wide range of biological processes such as proliferation, cell cycle regulation, apoptosis, differentiation, and DNA damage response [29, 30]. The *p300* protein functions primarily as a transcription cofactor for a number of nuclear proteins including known oncoproteins and tumor-suppressor proteins [29, 30]. An increasing body of evidence indicates that *p300* may be important in cancer [31, 32]. Interestingly, *p300* directly interacts with a transcription factor *NF-κB* as well as with the p65 protein that encoded by a gene *RELA*. The detection of frequent *C11orf95-RELA* gene fusion in supratentorial ependymomas further supports the potential role of *EP300* in ependymomas. Further functional investigation of *EP300* and other genes will provide pivotal information on the pathophysiology of ependymomas and potential therapeutic targets.

In summary, this study provides a comprehensive and global view of genes altered in SEPN. Importantly, enrichment of anterior/posterior pattern specification, response to oxidative stress, and cellular senescence genes distinguish the SEPN from intracranial ependymomas. We prioritized all chromosome 22 genes altered in ependymoma by comprehensive integration of distinct lines of evidence from different sources. The identification of these candidate genes provides an evidence-based rationale for functional studies that will help to further interrogate the initiation and/or progression of ependymomas.

## MATERIALS AND METHODS

### Microarray data

Three independent microarray datasets comprising a total of 262 expression profiles from tumors of ependymoma patients were used in this study. The supplementary table 6 reports the sample size, mean age, age range, gender, and the GSE identifier of the dataset in the Gene Expression Omnibus (GEO) database. Dataset 1 (CBTRC) contains tumor samples from intracranial (n = 71) and spinal (n = 12) regions of ependymoma patients with mean age of 10.6 and 25.6 years [33]. The CBTRC data were generated using the Affymetrix Human Genome U133 Plus 2.0 arrays. Dataset 2 (Toronto) consists of 85 intracranial tumors from patients with a mean age of 8.1 and 19 spinal tumors from patients with a mean of age of 35.6 years [34]. The Toronto data were generated using the Affymetrix human Exon 1.0 ST arrays. Dataset 3 (Heidelberg) consists of tumor samples from intracranial (n = 65) and spinal cord (n =10) of patients with mean age of 18.7 and 35.3 years [34]. The Heidelberg data were generated using the Agilent microarrays.

### Microarray data analysis

#### Pre-processing and normalization

Expression intensity values were calculated at probeset level for the 83 Affymetrix U133 Plus 2.0 CEL files using the robust multi-array average (RMA) method [35]. Probesets that are ‘absent’ (present / absent call using MAS5) in all samples were filtered out from the analysis. Expression values were mapped from probeset to unique gene and the probeset with the highest mean expression value was selected when multiple probesets were mapped to the same gene. The final filtering step left a total of 18,166 genes. For the Affymetrix Exon 1.0 ST arrays, we used the Affymetrix Power Tools (APT) to generate gene-level (core meta-probeset) expression values from raw CEL files. Arrays were normalized using RMA, which included RMA background correction, quantile normalization, log transformation, and probeset summarization. Detection above background (DABG) was performed at both the probe and the probeset level using GC-matched background probes, and low variance probesets were excluded (17,001 genes). For the Heidelberg data set, the pre-processed data was directly obtained from the GEO database. Probes with more than 30% missing values were filtered out and the probeset with the highest average expression value was retained when removing duplicate probesets for a gene (18,913 genes).

#### Differential expression analysis

Two different approaches were applied to the normalized data to identify differentially expressed genes between intracranial and spinal ependymomas. The first approach uses a meta-analysis that combines effect sizes from each dataset into a pooled effect size to estimate the amount of change in expression across all datasets. In each data set, the effect size was calculated for each gene using Hedges' adjusted g. A random effects model was used to combine effect sizes to obtain the pooled effect size and its standard error [36]. The z- statistic was computed as a ratio of the pooled effect size to its standard error for each gene, and the result was compared to a standard normal distribution to obtain a nominal p-value. P- values were corrected for multiple hypotheses testing using Benjamini-Hochberg correction. The second approach uses a non-parametric meta-analysis that combines p-values from individual studies. A moderated t-statistic based on empirical Bayesian method was calculated for each gene in each study [37]. Fisher's sum of logs was used to combine the p-values obtained from each study and were corrected for multiple hypotheses using Benjamini-Hochberg correction. The corrected P value, False Discovery Rate (FDR) less than 5% was used to select the differentially expressed genes between intracranial and spinal ependymomas.

#### NF2 gene correlation analysis

The correlation between NF2 gene expression and other genes was calculated using the Pearson correlation method for each data set separately. DerSimonian-Laird random-effect meta-analytical approach implemented in the metacor R package was used to combine correlation coefficients obtained from each individual datasets [38]. The correlation coefficients were transformed to Fisher's z-scores. P values obtained from the meta-analysis were corrected with Benjamini-Hochberg method. We used the Z-mean of correlation coefficients > 0.4 and FDR < 0.05 to select significantly correlated genes with NF2 gene expression.

### Gene set enrichment and pathway analysis

The over-representation analyses for Gene Ontology (GO) terms, Kyoto Encyclopedia of Genes and Genomes (KEGG), and Panther pathways, were carried out with GeneCodis [39]. The REVIGO software was used to summarize and visualize significant GO terms [40]. The overlap between differentially expressed genes and chromosomal positions (C1: positional gene sets collection) was investigated using the molecular signature database (MSigDB) version 4.0 [41]. The CS gene set was obtained from the published study [20]. The significance of statistically enriched functional categories, pathways, and gene sets was estimated with hypergeometric test and the p-values were corrected for the multiple comparisons by estimating the FDR.

### Prioritization of down regulated genes located in chromosome 22

Network analysis of protein-protein interactions (PPI) was used to prioritize significantly down regulated genes in SEPN that are located in chromosome 22. The PPI networks (including direct and indirect) among these genes were extracted from InWeb, a well-characterized PPI database developed by Lage *et al* [42]. To evaluate whether down regulated genes located in chromosome 22 are significantly connected via PPIs, permutation test was used to assess the significance of networks built from PPI data. Disease Association Protein-Protein Link Evaluator (DAPPLE) was used to assess the significance of PPI network with 10,000 permutations [43].

## SUPPLEMENTARY FIGURE AND TABLES














