# Supplementary material for: Expression alterations define unique molecular characteristics of spinal ependymomas
Source: Oncotarget. 2015 Mar 30;6(23):19780–91. doi: 10.18632/oncotarget.3715 (PMC4637320; doi:10.18632/oncotarget.3715)
Supplement: Supplementary file 1 [file oncotarget-06-19780-s001.pdf]

Expression alterations define unique molecular characteristics of spinal ependymomas

Supplementary Material

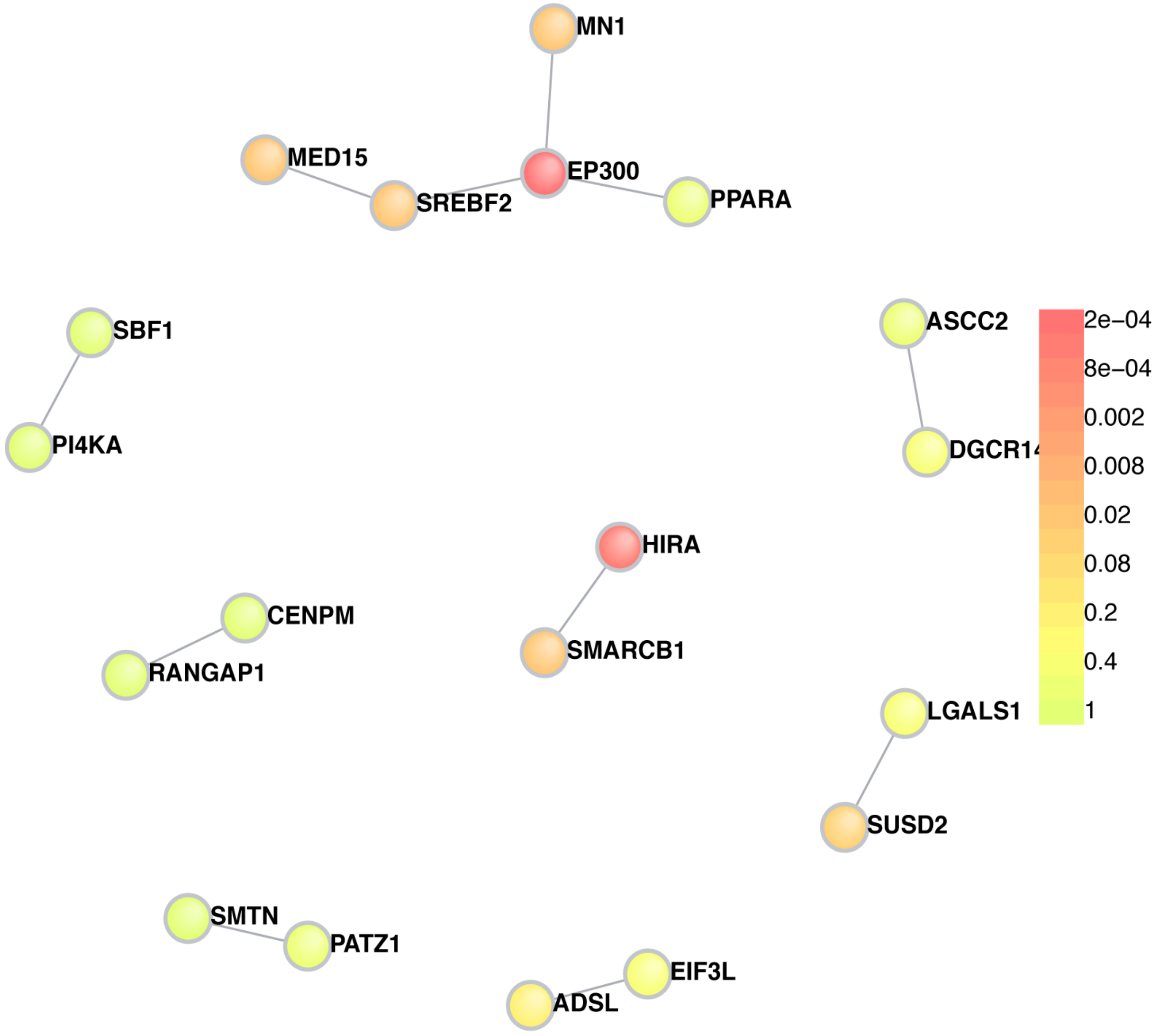

Supplementary Figure 1: Protein- protein interaction (PPI) network constructed with genes that were significantly down-regulated in SEPN and located on chromosome 22

Supplementary Table 1: Differentially expressed genes between spinal and intracranial ependymomas

Supplementary Table 2: Gene Ontology enrichment analysis

Supplementary Table 3: Enrichment of 34 cellular senescence genes among highly expressed genes in spinal ependymomas

Supplementary Table 4: Enrichment of chromosomal regions with significantly down-regulated genes by positional gene enrichment and gene set enrichment analysis methods.

Supplementary Table 5: Correlation of genes with NF2 gene expression

Supplementary Table 6: Description of gene expression microarray data sets used in the current analysis
