# Supplementary material for: Expression alterations define unique molecular characteristics of spinal ependymomas
Source: Oncotarget. 2015 Mar 30;6(23):19780–91. doi: 10.18632/oncotarget.3715 (PMC4637320; doi:10.18632/oncotarget.3715)
Supplement: Supplementary file 2 [file oncotarget-06-19780-s002.pdf]

Supplementary Table 1. **Differentially expressed genes between spinal and intracranial ependymomas**. Significantly differentially expressed genes identified by two meta-analysis approaches (i) by combining effect sizes (ES-based) and (ii) by combining P values (P values-based). Significant genes at FDR < 0.05 by both methods are considered as differentially expressed between spinal and intracranial ependymomas.

	Effect size (ES)-based meta-analysis			P values-based meta-analysis	
**Gene**	**ES**	**SE**	**FDR**	**S**	**FDR**
HOXB7	2.74	0.22	1.41E-31	183.27	0.00E+00
HOXC9	2.39	0.21	5.00E-26	152.65	0.00E+00
HOXB8	2.24	0.21	1.33E-23	137.87	0.00E+00
HOXB6	2.15	0.20	4.12E-22	130.37	0.00E+00
CFTR	3.52	0.34	5.33E-22	256.51	0.00E+00
HOXB5	2.13	0.22	3.72E-19	130.08	0.00E+00
ACSS1	-1.89	0.20	6.54E-18	108.78	0.00E+00
PLA2R1	1.88	0.20	8.18E-18	106.47	0.00E+00
MS4A2	1.86	0.20	1.41E-17	103.81	0.00E+00
TMOD1	1.78	0.20	3.13E-16	97.91	0.00E+00
CTTNBP2	1.77	0.20	4.29E-16	96.98	0.00E+00
HOXD8	1.77	0.20	4.29E-16	96.28	0.00E+00
JPH2	2.34	0.26	4.29E-16	146.37	0.00E+00
EID1	1.75	0.20	7.06E-16	92.29	0.00E+00
C1GALT1C1	1.74	0.20	1.28E-15	92.45	0.00E+00
C7orf31	1.73	0.20	1.61E-15	92.22	0.00E+00
ZIC2	-1.72	0.20	2.45E-15	93.01	0.00E+00
IGF1	1.70	0.20	3.56E-15	92.42	0.00E+00
HOXA7	2.48	0.29	5.84E-15	163.58	0.00E+00
APBA2	-1.69	0.20	5.84E-15	90.95	0.00E+00
KCNJ16	1.71	0.20	6.06E-15	94.40	0.00E+00
ZIC1	-1.71	0.20	9.23E-15	94.46	0.00E+00
CD47	1.67	0.20	1.07E-14	89.83	0.00E+00
INHBA	1.67	0.20	1.10E-14	89.43	0.00E+00
CADPS2	1.66	0.20	1.32E-14	88.45	3.67E-14
PDLIM1	1.63	0.19	3.80E-14	85.48	7.17E-14
HOXA5	1.86	0.22	3.92E-14	107.15	0.00E+00
RHBDD2	1.61	0.19	7.35E-14	82.63	2.67E-13
KCNMB1	1.60	0.19	1.35E-13	82.59	2.67E-13
MIR31HG	1.59	0.19	1.52E-13	82.94	2.57E-13
NR4A3	1.59	0.19	1.63E-13	82.59	2.67E-13
HPSE2	1.59	0.19	1.91E-13	81.97	3.71E-13
WBSCR17	1.57	0.19	3.27E-13	80.38	6.94E-13
KCNE1	1.56	0.19	3.93E-13	80.84	5.95E-13
SHROOM3	1.56	0.19	4.47E-13	80.35	6.94E-13
CPNE4	1.78	0.22	4.66E-13	100.10	0.00E+00
LRRC2	1.55	0.19	5.37E-13	79.55	1.01E-12
FAM122C	1.54	0.19	7.12E-13	79.45	1.04E-12
ANXA4	1.76	0.22	9.55E-13	97.68	0.00E+00
SALL1	1.90	0.24	1.06E-12	110.66	0.00E+00
EPSTI1	1.52	0.19	1.39E-12	76.47	3.49E-12
ATXN1	1.51	0.19	2.21E-12	75.70	4.86E-12
ME1	1.53	0.20	2.52E-12	79.34	1.08E-12
YPEL5	1.49	0.19	4.03E-12	74.12	9.64E-12
PDXK	-1.49	0.19	4.16E-12	73.09	1.48E-11
CLDN1	1.49	0.19	4.46E-12	74.31	9.01E-12
APOA1BP	1.49	0.19	4.53E-12	73.76	1.10E-11

NAV1	-1.47	0.19	8.40E-12	72.35	2.05E-11
SHC4	1.53	0.20	8.51E-12	78.89	1.23E-12
RAB9A	1.46	0.19	9.51E-12	70.99	3.60E-11
FXN	1.46	0.19	9.75E-12	71.63	2.82E-11
ARX	1.54	0.20	1.26E-11	78.83	1.23E-12
F8	1.45	0.19	1.67E-11	71.09	3.50E-11
GPR161	-1.44	0.19	1.81E-11	70.28	4.56E-11
FGF1	1.44	0.19	1.92E-11	70.89	3.62E-11
SCML4	1.44	0.19	2.16E-11	68.37	9.98E-11
NALCN	-1.44	0.19	2.24E-11	70.85	3.65E-11
HAS2	1.44	0.19	2.31E-11	70.94	3.61E-11
DACH1	-1.42	0.19	3.91E-11	69.26	6.93E-11
PRUNE2	1.42	0.19	4.30E-11	68.38	9.98E-11
SEPT11	1.41	0.19	4.68E-11	68.34	1.00E-10
CXCL12	1.41	0.19	5.41E-11	68.82	8.34E-11
DCDC2	1.59	0.22	5.76E-11	84.69	9.84E-14
MFSD6	1.41	0.19	5.94E-11	69.35	6.78E-11
PRICKLE2	1.40	0.19	5.94E-11	67.68	1.28E-10
RNF43	-1.40	0.19	6.49E-11	67.55	1.36E-10
GPD1	1.40	0.19	6.98E-11	68.32	1.01E-10
SNX31	1.39	0.19	9.24E-11	66.71	1.93E-10
FXYD6	-1.39	0.19	9.44E-11	67.70	1.28E-10
HCCS	1.38	0.19	1.15E-10	65.72	2.92E-10
GPM6A	-1.37	0.19	1.59E-10	65.50	3.23E-10
CNTFR	-1.37	0.19	1.70E-10	64.98	4.04E-10
IKZF2	1.49	0.21	1.72E-10	75.81	4.65E-12
TCTN3	1.37	0.19	1.72E-10	64.92	4.10E-10
LCLAT1	1.36	0.19	2.36E-10	64.00	6.15E-10
IFT20	1.36	0.19	2.76E-10	63.83	6.57E-10
BEGAIN	-1.36	0.19	2.76E-10	65.05	3.94E-10
CYP2R1	-1.37	0.19	2.88E-10	66.14	2.41E-10
TCF20	-1.35	0.19	3.14E-10	62.96	9.24E-10
RNLS	1.34	0.19	4.13E-10	62.35	1.18E-09
BEX1	1.34	0.19	4.14E-10	62.76	9.84E-10
SYNDIG1	-1.34	0.19	4.26E-10	62.85	9.61E-10
TGFB3	-1.34	0.19	4.26E-10	63.62	7.12E-10
PCSK1N	1.33	0.19	5.89E-10	62.80	9.74E-10
AKAP9	1.33	0.19	6.23E-10	62.90	9.47E-10
CPE	-1.33	0.19	6.41E-10	63.95	6.25E-10
RS1	1.32	0.19	7.68E-10	60.25	2.87E-09
HTR7P1	1.32	0.19	8.44E-10	59.86	3.37E-09
VEPH1	1.57	0.23	8.62E-10	82.65	2.67E-13
DGCR2	-1.32	0.19	8.62E-10	60.25	2.87E-09
PHF21B	-1.43	0.21	8.62E-10	70.49	4.24E-11
HSPE1	1.32	0.19	8.78E-10	60.29	2.84E-09
IFT57	1.54	0.22	1.01E-09	80.47	6.91E-13
IRAK1	1.31	0.19	1.19E-09	59.38	4.02E-09
ECI2	1.30	0.19	1.28E-09	59.98	3.21E-09
LEPREL1	1.30	0.19	1.32E-09	60.13	3.01E-09
USP13	-1.30	0.19	1.59E-09	58.87	4.96E-09
EPB41L4A	1.30	0.19	1.63E-09	59.55	3.78E-09
CPT1C	-1.34	0.20	2.00E-09	64.71	4.46E-10
MPST	-1.29	0.19	2.08E-09	57.65	8.17E-09
CTGF	1.33	0.20	2.09E-09	63.77	6.71E-10
LRCH2	1.29	0.19	2.11E-09	58.68	5.40E-09
DIMT1	1.28	0.19	2.15E-09	57.60	8.35E-09
TOM1L1	1.28	0.19	2.15E-09	58.07	6.93E-09
EPB41L3	1.28	0.19	2.22E-09	59.18	4.39E-09

HIATL1	1.28	0.19	2.62E-09	57.37	9.07E-09
CLIP1	1.28	0.19	2.66E-09	57.91	7.39E-09
KCNA2	-1.28	0.19	2.67E-09	59.70	3.57E-09
GYS1	-1.27	0.19	2.97E-09	57.41	9.03E-09
FAM110B	-1.27	0.19	2.99E-09	58.48	5.87E-09
ZDHHC4	1.27	0.19	3.25E-09	57.31	9.14E-09
RAB11A	1.27	0.19	3.37E-09	56.54	1.24E-08
JAM2	1.27	0.19	3.50E-09	57.37	9.07E-09
WBP5	1.26	0.19	3.88E-09	57.29	9.14E-09
NCAN	-1.26	0.19	3.97E-09	57.29	9.14E-09
DENND1A	1.26	0.19	4.02E-09	56.53	1.24E-08
STARD3NL	1.26	0.19	4.02E-09	55.36	1.97E-08
NLK	1.26	0.19	4.06E-09	57.48	8.79E-09
CCDC28A	1.26	0.19	4.20E-09	56.67	1.17E-08
MTHFR	-1.34	0.20	4.40E-09	63.09	8.81E-10
IFNE	1.25	0.19	4.66E-09	56.16	1.45E-08
ZNF462	1.26	0.19	4.66E-09	57.30	9.14E-09
POR	1.26	0.19	4.79E-09	55.46	1.90E-08
PHF17	1.26	0.19	4.82E-09	57.14	9.59E-09
CYGB	1.35	0.20	5.43E-09	64.70	4.46E-10
H2AFJ	1.24	0.19	6.55E-09	55.33	2.00E-08
IGFBP7	1.24	0.19	6.86E-09	55.78	1.66E-08
COL22A1	1.24	0.19	7.25E-09	55.14	2.13E-08
TBC1D16	-1.24	0.19	7.76E-09	54.52	2.70E-08
YPEL1	-1.24	0.19	7.76E-09	56.01	1.53E-08
SGCE	1.24	0.19	8.06E-09	55.16	2.12E-08
NTN1	1.28	0.20	8.12E-09	59.75	3.51E-09
VTN	1.90	0.29	9.22E-09	110.93	0.00E+00
CECR5	-1.23	0.19	9.22E-09	53.21	4.52E-08
CBS	-1.23	0.19	9.62E-09	55.49	1.88E-08
TDRD7	1.23	0.19	9.84E-09	54.70	2.54E-08
ATP8A1	-1.29	0.20	9.84E-09	60.70	2.41E-09
CAST	1.23	0.19	9.89E-09	55.41	1.94E-08
SLC35A2	1.23	0.19	1.05E-08	52.17	6.62E-08
PAMR1	1.23	0.19	1.06E-08	55.10	2.16E-08
MTUS1	1.23	0.19	1.10E-08	54.12	3.16E-08
SNCA	1.26	0.19	1.12E-08	58.58	5.63E-09
FBLN1	-1.22	0.19	1.20E-08	54.79	2.46E-08
RAB11FIP1	1.22	0.19	1.32E-08	53.41	4.20E-08
LINGO1	-1.22	0.19	1.37E-08	55.22	2.08E-08
UCHL1	1.22	0.19	1.41E-08	54.12	3.16E-08
BCR	-1.21	0.19	1.57E-08	54.66	2.56E-08
LPAR3	-1.21	0.19	1.57E-08	53.87	3.48E-08
SNED1	-1.24	0.19	1.57E-08	57.00	1.02E-08
SMPDL3A	1.56	0.24	1.61E-08	81.54	4.21E-13
TPM1	1.21	0.19	1.67E-08	53.77	3.60E-08
TSPAN31	1.21	0.19	1.67E-08	52.29	6.37E-08
ARHGEF17	-1.21	0.19	1.67E-08	52.04	7.00E-08
SLC27A6	1.21	0.19	1.70E-08	53.27	4.42E-08
NAE1	1.21	0.19	1.78E-08	53.26	4.43E-08
DOK6	-1.21	0.19	1.84E-08	53.83	3.53E-08
UXS1	1.20	0.19	2.01E-08	52.35	6.30E-08
EMID1	-1.20	0.19	2.04E-08	53.07	4.76E-08
CCND1	-1.20	0.19	2.09E-08	54.17	3.14E-08
APC2	-1.50	0.23	2.26E-08	75.96	4.39E-12
NIF3L1	1.20	0.19	2.36E-08	49.62	1.85E-07
TMEM139	1.19	0.19	2.65E-08	50.54	1.31E-07
KCNJ12	-1.19	0.19	2.68E-08	53.17	4.59E-08

FABP7	1.19	0.19	2.76E-08	52.66	5.58E-08
PISD	-1.19	0.19	2.77E-08	49.83	1.72E-07
EVL	-1.19	0.19	2.85E-08	52.03	7.00E-08
PWP2	-1.19	0.19	3.16E-08	48.88	2.50E-07
IMMP2L	1.19	0.19	3.40E-08	51.24	9.79E-08
KAZN	-1.18	0.19	3.78E-08	52.19	6.61E-08
RCAN1	1.18	0.19	3.90E-08	51.12	1.03E-07
SCN1A	1.35	0.21	4.00E-08	65.29	3.53E-10
LAYN	1.18	0.19	4.08E-08	51.39	9.24E-08
CERK	-1.17	0.19	4.36E-08	50.25	1.46E-07
CALB1	-1.17	0.19	4.42E-08	50.86	1.15E-07
DUSP14	1.18	0.19	4.43E-08	51.31	9.55E-08
PSD2	-1.17	0.19	4.48E-08	50.94	1.11E-07
NUP37	1.18	0.19	4.56E-08	51.36	9.36E-08
SMCR7L	-1.17	0.19	4.68E-08	50.24	1.46E-07
MPL	1.17	0.19	4.77E-08	50.56	1.30E-07
SGSM3	-1.41	0.23	5.17E-08	67.73	1.28E-10
HN1	-1.37	0.22	5.25E-08	66.51	2.07E-10
XPA	1.17	0.19	5.40E-08	50.03	1.60E-07
SEC14L2	-1.17	0.19	5.40E-08	50.29	1.44E-07
HTRA1	1.16	0.19	5.85E-08	49.75	1.77E-07
TMEM185A	1.17	0.19	5.85E-08	49.97	1.63E-07
AGFG1	1.16	0.19	5.98E-08	48.89	2.49E-07
TMEM187	1.16	0.19	6.01E-08	50.07	1.57E-07
TTC38	-1.16	0.19	6.09E-08	49.40	2.03E-07
TEX261	1.16	0.19	6.33E-08	49.53	1.93E-07
ANKRD6	1.41	0.23	6.88E-08	69.96	5.18E-11
CCDC53	1.16	0.19	6.88E-08	49.01	2.38E-07
SIL1	1.16	0.19	6.88E-08	48.60	2.80E-07
PYGB	-1.16	0.19	7.29E-08	48.98	2.41E-07
FAT4	-1.16	0.19	7.37E-08	50.21	1.47E-07
DOCK1	-1.15	0.19	8.07E-08	50.17	1.50E-07
EFHB	1.15	0.19	8.38E-08	50.47	1.35E-07
DNAJC15	1.15	0.19	8.46E-08	50.30	1.43E-07
SIRPA	-1.15	0.19	8.64E-08	49.14	2.26E-07
ABTB2	-1.78	0.29	8.84E-08	99.67	0.00E+00
A4GALT	-1.15	0.19	9.03E-08	48.03	3.52E-07
STAG3	1.15	0.19	9.08E-08	48.59	2.80E-07
RP2	1.15	0.19	9.27E-08	49.09	2.31E-07
CXXC4	1.15	0.19	9.80E-08	49.68	1.81E-07
WDR44	1.14	0.19	9.99E-08	48.02	3.52E-07
SEPT9	-1.29	0.21	9.99E-08	60.57	2.55E-09
ABCD4	-1.14	0.19	1.06E-07	47.51	4.32E-07
TRMT2A	-1.14	0.19	1.10E-07	45.97	7.76E-07
GCLC	1.14	0.19	1.11E-07	49.78	1.76E-07
CACNB4	1.14	0.19	1.13E-07	48.93	2.45E-07
GAL3ST3	1.14	0.19	1.26E-07	48.39	3.02E-07
PAQR4	-1.14	0.19	1.26E-07	48.68	2.71E-07
PPFIA1	-1.13	0.19	1.37E-07	47.86	3.76E-07
KLHL23	-1.13	0.19	1.38E-07	47.68	4.04E-07
RSBN1L	1.13	0.19	1.39E-07	46.94	5.40E-07
INPP5D	1.13	0.19	1.40E-07	48.64	2.75E-07
SRP14	1.25	0.21	1.40E-07	56.19	1.43E-08
PPP6R2	-1.13	0.19	1.44E-07	47.10	5.07E-07
HS1BP3	1.13	0.19	1.46E-07	46.31	6.82E-07
RTN4IP1	1.13	0.19	1.46E-07	47.86	3.76E-07
WWP1	1.13	0.19	1.46E-07	46.84	5.59E-07
GDF9	1.13	0.19	1.49E-07	46.53	6.30E-07

DTX1	-1.13	0.19	1.64E-07	47.79	3.88E-07
PRKAR2B	1.20	0.20	1.71E-07	54.01	3.31E-08
TAB1	-1.19	0.20	1.73E-07	50.50	1.34E-07
ZMIZ1	-1.12	0.19	1.79E-07	47.88	3.75E-07
DPYSL4	-1.12	0.19	1.89E-07	46.76	5.77E-07
EDA	-1.12	0.19	1.92E-07	47.28	4.73E-07
MKL1	-1.12	0.19	1.95E-07	45.49	9.33E-07
GLA	1.12	0.19	1.96E-07	46.13	7.35E-07
MEIS2	1.11	0.19	2.13E-07	46.72	5.84E-07
WDR6	-1.11	0.19	2.21E-07	45.38	9.79E-07
C9orf156	1.11	0.19	2.39E-07	46.98	5.32E-07
PDCD4	1.11	0.19	2.39E-07	47.48	4.38E-07
PLS3	1.11	0.19	2.39E-07	46.32	6.82E-07
PGRMC1	1.11	0.19	2.45E-07	47.61	4.14E-07
MECOM	-1.11	0.19	2.49E-07	47.35	4.60E-07
COL5A1	1.11	0.19	2.68E-07	47.01	5.27E-07
ATP8B4	1.20	0.20	2.70E-07	54.52	2.70E-08
CCNDBP1	1.11	0.19	2.70E-07	47.17	4.94E-07
TCEAL1	1.11	0.19	2.70E-07	46.40	6.59E-07
MORN2	1.11	0.19	2.74E-07	46.32	6.82E-07
SLC22A23	-1.10	0.19	2.81E-07	46.91	5.47E-07
ARSE	1.10	0.19	2.86E-07	45.79	8.34E-07
C8orf4	1.10	0.19	2.90E-07	47.87	3.75E-07
NFU1	1.10	0.19	2.98E-07	45.52	9.21E-07
RND2	1.10	0.19	2.98E-07	45.68	8.69E-07
DMBT1	1.10	0.19	3.01E-07	44.38	1.43E-06
PPP1R14C	1.37	0.23	3.02E-07	67.53	1.36E-10
MMP17	-1.10	0.19	3.28E-07	46.01	7.70E-07
CSPG4	-1.10	0.19	3.42E-07	45.30	1.00E-06
PCBP3	-1.23	0.21	3.42E-07	55.90	1.58E-08
RRAGA	1.19	0.20	3.44E-07	52.90	5.02E-08
HIF3A	-1.09	0.19	3.55E-07	45.77	8.39E-07
CD200	1.09	0.19	3.72E-07	45.86	8.12E-07
ECE1	-1.09	0.19	3.80E-07	45.67	8.70E-07
FAM175A	1.09	0.19	3.87E-07	45.00	1.13E-06
GLB1L	1.09	0.19	3.90E-07	45.99	7.75E-07
PAFAH1B3	-1.16	0.20	3.90E-07	49.91	1.66E-07
PLCL2	-1.09	0.19	3.90E-07	45.34	9.92E-07
AP3S1	1.09	0.19	3.93E-07	45.13	1.07E-06
CMTM4	1.09	0.19	3.93E-07	44.72	1.25E-06
SBF1	-1.09	0.19	3.93E-07	44.02	1.66E-06
C10orf118	1.09	0.19	4.05E-07	45.13	1.07E-06
HEG1	1.09	0.19	4.41E-07	44.83	1.21E-06
TMEM63A	1.08	0.19	4.68E-07	44.76	1.24E-06
LMCD1	1.08	0.19	4.75E-07	45.56	9.09E-07
ZBTB8A	-1.08	0.19	4.86E-07	44.66	1.28E-06
PROCR	1.44	0.25	5.02E-07	72.25	2.13E-11
C21orf62	1.66	0.29	5.07E-07	91.03	0.00E+00
TBC1D4	1.08	0.19	5.20E-07	45.96	7.80E-07
IDS	1.08	0.19	5.27E-07	45.11	1.08E-06
ST3GAL4	-1.08	0.19	5.27E-07	43.46	2.06E-06
ZBTB17	-1.08	0.19	5.36E-07	41.57	4.23E-06
MAPT	-1.08	0.19	5.51E-07	45.51	9.28E-07
TTC39B	1.08	0.19	5.75E-07	44.62	1.30E-06
BLVRA	1.07	0.19	5.82E-07	43.37	2.14E-06
ZDHHC22	-1.07	0.19	5.97E-07	43.92	1.72E-06
ARL1	1.07	0.19	6.08E-07	43.95	1.71E-06
SCAMP1	1.07	0.19	6.10E-07	44.39	1.42E-06

TLR4	1.07	0.19	6.16E-07	44.46	1.38E-06
HOXB3	1.07	0.19	6.22E-07	44.43	1.40E-06
FLNC	1.07	0.19	6.33E-07	44.78	1.23E-06
RCN2	1.07	0.19	6.39E-07	42.98	2.48E-06
ITPR2	1.07	0.19	7.63E-07	43.53	2.01E-06
ZFR	1.10	0.19	7.64E-07	46.28	6.90E-07
RAPGEF3	-1.06	0.19	7.99E-07	43.16	2.32E-06
DOCK5	1.06	0.19	8.27E-07	43.06	2.40E-06
STXBP4	1.06	0.19	8.60E-07	43.03	2.43E-06
COQ3	1.06	0.19	8.66E-07	41.60	4.20E-06
FAIM	1.06	0.19	8.66E-07	43.33	2.17E-06
HOXA2	1.06	0.19	8.66E-07	43.18	2.31E-06
ROBO1	1.06	0.19	8.73E-07	43.07	2.40E-06
SREBF2	-1.06	0.19	8.87E-07	42.95	2.50E-06
SNPH	-1.06	0.19	9.21E-07	42.38	3.12E-06
VSIG1	1.14	0.20	9.22E-07	48.30	3.12E-07
AES	1.06	0.19	9.23E-07	43.52	2.01E-06
KRIT1	1.06	0.19	9.29E-07	42.40	3.11E-06
CDH4	-1.54	0.27	9.31E-07	79.20	1.10E-12
PTN	-1.06	0.19	9.37E-07	43.24	2.25E-06
EZH2	-1.06	0.19	9.46E-07	43.08	2.39E-06
MAP1LC3A	1.06	0.19	9.92E-07	43.68	1.89E-06
AGAP1	1.32	0.23	1.04E-06	63.21	8.36E-10
CTNNA1	1.05	0.19	1.07E-06	42.04	3.56E-06
FAM124A	1.07	0.19	1.07E-06	45.23	1.03E-06
RRP7A	-1.05	0.19	1.07E-06	41.87	3.79E-06
FGFRL1	-1.05	0.19	1.09E-06	42.48	3.01E-06
ACSM1	1.15	0.20	1.11E-06	50.35	1.41E-07
SH3GL2	1.05	0.19	1.22E-06	42.83	2.62E-06
ANKRD13B	-1.05	0.19	1.30E-06	42.83	2.62E-06
ANKRD27	-1.04	0.19	1.37E-06	41.69	4.07E-06
KLHL22	-1.12	0.20	1.38E-06	47.61	4.14E-07
CHST1	-1.04	0.19	1.39E-06	41.60	4.20E-06
CLTA	1.04	0.19	1.48E-06	40.43	6.58E-06
TCTN2	1.15	0.21	1.51E-06	50.73	1.21E-07
SFRP4	1.04	0.19	1.56E-06	42.70	2.76E-06
AIFM3	-1.04	0.19	1.62E-06	43.18	2.31E-06
LSAMP	-1.04	0.19	1.62E-06	42.95	2.50E-06
COPS6	1.04	0.19	1.64E-06	40.83	5.63E-06
SMARCA1	1.04	0.19	1.65E-06	42.42	3.09E-06
NECAB2	1.13	0.20	1.67E-06	48.46	2.94E-07
RASL10A	-1.03	0.19	1.68E-06	41.55	4.25E-06
DBI	-1.03	0.19	1.71E-06	42.24	3.29E-06
PARD3B	1.03	0.19	1.72E-06	41.48	4.37E-06
NOX4	-1.03	0.19	1.73E-06	42.03	3.58E-06
LGALS3	-1.03	0.19	1.85E-06	41.80	3.89E-06
FBXO2	-1.03	0.19	1.87E-06	42.26	3.26E-06
ASAP1-IT1	-1.03	0.19	1.89E-06	41.61	4.18E-06
STAU1	1.03	0.19	1.95E-06	40.31	6.92E-06
MMP16	-1.03	0.19	1.95E-06	40.87	5.56E-06
FRS2	1.03	0.19	1.96E-06	41.79	3.91E-06
SLC12A4	1.03	0.19	2.02E-06	41.09	5.12E-06
LDB2	-1.03	0.19	2.03E-06	40.99	5.31E-06
EIF4E3	1.03	0.19	2.07E-06	41.65	4.14E-06
GCDH	-1.02	0.19	2.16E-06	40.93	5.44E-06
CCDC69	1.02	0.19	2.17E-06	40.71	5.90E-06
DDX39A	-1.02	0.19	2.25E-06	40.09	7.51E-06
DIRAS2	1.55	0.28	2.33E-06	81.11	5.23E-13

LETM1	-1.02	0.19	2.33E-06	39.19	1.07E-05
PSMD5	1.02	0.19	2.36E-06	39.82	8.38E-06
SLC25A22	-1.02	0.19	2.41E-06	39.73	8.64E-06
RHOBTB3	-1.02	0.19	2.50E-06	40.54	6.28E-06
DZIP3	1.02	0.19	2.51E-06	41.69	4.07E-06
MAD2L2	-1.02	0.19	2.51E-06	41.40	4.50E-06
ROBO3	-1.02	0.19	2.52E-06	40.79	5.70E-06
SUGP1	-1.02	0.19	2.54E-06	37.62	1.98E-05
UNC5B	-1.02	0.19	2.59E-06	40.15	7.35E-06
PPEF1	1.02	0.19	2.60E-06	40.68	5.96E-06
PPARA	-1.02	0.19	2.63E-06	40.93	5.44E-06
C20orf85	1.01	0.19	2.70E-06	40.16	7.32E-06
ZFYVE1	-1.02	0.19	2.70E-06	40.57	6.23E-06
BCO2	1.04	0.19	2.72E-06	43.53	2.01E-06
DNAH9	1.01	0.19	2.73E-06	41.20	4.91E-06
RAI14	1.01	0.19	2.91E-06	40.30	6.93E-06
CPEB1	1.01	0.19	3.03E-06	40.59	6.17E-06
MC4R	1.01	0.19	3.11E-06	39.80	8.41E-06
TESC	-1.01	0.19	3.17E-06	41.57	4.23E-06
FHL1	1.36	0.25	3.25E-06	67.23	1.55E-10
ELN	-1.01	0.19	3.25E-06	39.72	8.67E-06
ENPP5	1.01	0.19	3.31E-06	40.89	5.52E-06
SKAP2	1.36	0.25	3.31E-06	66.83	1.85E-10
GOT2	1.04	0.19	3.33E-06	43.41	2.11E-06
ST6GALNAC2	1.00	0.19	3.55E-06	39.96	7.89E-06
BMP4	-1.00	0.19	3.84E-06	40.99	5.31E-06
ARL10	-1.00	0.19	3.85E-06	38.69	1.31E-05
CRMP1	-1.00	0.19	4.04E-06	39.07	1.13E-05
ZXDA	1.00	0.19	4.08E-06	38.56	1.38E-05
THUMPD1	1.39	0.26	4.10E-06	67.80	1.26E-10
RBM24	1.40	0.26	4.33E-06	70.95	3.61E-11
H1F0	-1.00	0.19	4.33E-06	39.63	9.02E-06
KLF9	0.99	0.19	4.46E-06	39.36	1.00E-05
SLC8A1	0.99	0.19	4.46E-06	39.27	1.04E-05
STK16	0.99	0.19	4.46E-06	37.63	1.97E-05
TCEB2	0.99	0.19	4.57E-06	38.83	1.24E-05
DNASE1L1	0.99	0.19	4.64E-06	38.84	1.23E-05
ERP29	0.99	0.19	4.64E-06	38.17	1.60E-05
PEX1	0.99	0.19	4.65E-06	37.17	2.31E-05
TCEA3	0.99	0.19	4.65E-06	39.19	1.07E-05
TAF9B	0.99	0.19	4.68E-06	39.43	9.73E-06
PARP3	1.07	0.20	4.79E-06	44.03	1.65E-06
PNPO	0.99	0.19	4.82E-06	38.77	1.27E-05
ITGA7	-0.99	0.19	4.82E-06	39.91	8.07E-06
UHRF1	-0.99	0.19	4.98E-06	39.11	1.11E-05
CBX4	-0.99	0.19	5.17E-06	38.74	1.28E-05
LONRF2	1.14	0.21	5.18E-06	50.41	1.38E-07
FZD2	-1.10	0.21	5.22E-06	47.27	4.73E-07
CACNB1	-1.00	0.19	5.25E-06	40.95	5.40E-06
EYA4	0.99	0.19	5.37E-06	39.99	7.83E-06
C18orf25	0.99	0.19	5.39E-06	38.42	1.46E-05
KCTD9	0.98	0.19	5.46E-06	38.11	1.64E-05
CREB3	1.01	0.19	5.61E-06	40.90	5.48E-06
C12orf75	1.13	0.21	5.64E-06	49.95	1.63E-07
RGMA	0.98	0.19	5.69E-06	38.66	1.32E-05
BAG1	0.98	0.19	5.70E-06	37.38	2.15E-05
DCLK1	-0.98	0.19	5.74E-06	38.11	1.64E-05
RTN1	0.98	0.19	5.95E-06	39.44	9.71E-06

SLC6A12	-0.98	0.19	6.14E-06	37.91	1.78E-05
ACOT9	0.98	0.19	6.24E-06	37.72	1.91E-05
PSMD10	0.98	0.19	6.25E-06	38.29	1.53E-05
CNPY4	0.98	0.19	6.27E-06	37.55	2.03E-05
PAG1	-0.98	0.19	6.34E-06	38.26	1.54E-05
MYOF	0.98	0.19	6.48E-06	39.57	9.21E-06
BET1	0.98	0.19	6.54E-06	37.87	1.80E-05
NTM	-0.98	0.19	6.60E-06	38.35	1.50E-05
SLC19A1	-1.06	0.20	6.61E-06	43.67	1.90E-06
BMX	0.98	0.19	6.66E-06	36.54	2.91E-05
EYA2	-0.98	0.19	6.66E-06	38.40	1.46E-05
TMEM108	0.97	0.19	6.90E-06	38.36	1.49E-05
C11orf71	1.00	0.19	7.04E-06	40.64	6.04E-06
ERLEC1	1.02	0.19	7.06E-06	41.57	4.23E-06
MCOLN2	1.06	0.20	7.09E-06	44.75	1.24E-06
SULF1	0.97	0.19	7.20E-06	38.74	1.28E-05
CBX6	-0.97	0.19	7.36E-06	38.89	1.21E-05
MORC4	0.97	0.19	7.44E-06	37.18	2.31E-05
UPRT	1.21	0.23	7.49E-06	54.12	3.16E-08
ANXA2	0.97	0.19	7.50E-06	38.22	1.57E-05
SPATA18	0.97	0.19	7.61E-06	37.82	1.84E-05
STIM1	1.07	0.20	7.66E-06	44.82	1.21E-06
HTR2A	-0.97	0.19	7.68E-06	37.59	2.00E-05
COL4A3	0.97	0.19	7.69E-06	37.46	2.09E-05
NTN4	0.97	0.19	7.83E-06	37.68	1.94E-05
SLC18A2	0.97	0.19	7.99E-06	38.34	1.50E-05
C10orf67	0.97	0.19	8.04E-06	37.34	2.19E-05
C1orf94	1.20	0.23	8.05E-06	53.39	4.23E-08
PCP4	-0.98	0.19	8.20E-06	40.26	7.03E-06
KIAA0020	0.97	0.19	8.28E-06	36.83	2.62E-05
FANK1	0.97	0.19	8.32E-06	38.78	1.26E-05
NAT2	1.20	0.23	8.32E-06	54.86	2.40E-08
PORCN	0.97	0.19	8.32E-06	35.90	3.70E-05
PDE7A	1.49	0.29	8.39E-06	78.17	1.65E-12
MYO3A	-0.97	0.19	8.39E-06	38.54	1.39E-05
SUFU	-0.97	0.19	8.52E-06	37.15	2.33E-05
TCEAL4	0.97	0.19	8.56E-06	36.80	2.65E-05
RHPN2	0.96	0.19	8.72E-06	37.22	2.28E-05
GLI2	-0.96	0.19	8.72E-06	37.40	2.14E-05
PAQR6	-0.96	0.19	8.91E-06	37.17	2.31E-05
STH	-0.97	0.19	8.91E-06	38.98	1.17E-05
ZNF74	-0.96	0.19	9.10E-06	35.13	4.98E-05
RAB40C	-0.96	0.19	9.14E-06	37.25	2.26E-05
NYNRIN	-0.96	0.19	9.20E-06	37.27	2.25E-05
ALG13	0.96	0.19	9.26E-06	36.73	2.73E-05
ITPKC	-0.96	0.19	9.26E-06	37.05	2.41E-05
NDUFB2	1.10	0.21	9.27E-06	46.90	5.49E-07
C9orf78	1.03	0.20	9.29E-06	41.69	4.07E-06
NDFIP1	0.96	0.19	9.32E-06	37.22	2.28E-05
EBAG9	0.96	0.19	9.34E-06	37.05	2.41E-05
IQCG	0.96	0.19	9.34E-06	38.00	1.71E-05
CLSTN2	0.96	0.19	9.46E-06	36.92	2.53E-05
NCAPH2	-0.96	0.19	9.48E-06	35.86	3.76E-05
CHCHD6	1.07	0.21	9.65E-06	44.55	1.34E-06
FLRT1	-1.27	0.25	9.65E-06	59.08	4.58E-09
EPDR1	0.96	0.19	9.67E-06	36.75	2.71E-05
CNKSR3	0.96	0.19	9.94E-06	37.40	2.14E-05
ZNF688	1.09	0.21	9.94E-06	46.43	6.57E-07

ADARB2	-0.96	0.19	1.01E-05	37.13	2.34E-05
CHDH	-1.08	0.21	1.01E-05	45.99	7.75E-07
SAT1	0.96	0.19	1.04E-05	37.01	2.45E-05
USP11	0.96	0.19	1.04E-05	37.37	2.16E-05
C16orf71	0.96	0.19	1.05E-05	37.24	2.27E-05
GCLM	0.96	0.19	1.05E-05	37.62	1.98E-05
RELN	-0.95	0.19	1.07E-05	37.72	1.91E-05
APOBEC3G	0.95	0.19	1.08E-05	36.47	2.99E-05
MAGOH	0.96	0.19	1.09E-05	37.84	1.83E-05
GNAQ	1.20	0.23	1.12E-05	54.00	3.31E-08
THAP5	0.95	0.19	1.12E-05	37.24	2.27E-05
ITGB8	0.96	0.19	1.13E-05	38.26	1.54E-05
EDNRA	-0.95	0.18	1.18E-05	36.32	3.15E-05
SRI	0.95	0.19	1.19E-05	36.91	2.54E-05
TOR1A	1.16	0.23	1.22E-05	51.25	9.76E-08
ANXA1	0.95	0.18	1.23E-05	36.09	3.44E-05
F3	-0.95	0.19	1.24E-05	37.32	2.20E-05
TCFL5	-0.95	0.18	1.24E-05	36.07	3.46E-05
RAB39B	0.95	0.19	1.25E-05	37.18	2.31E-05
ZNF423	-1.50	0.29	1.25E-05	77.50	2.24E-12
EFS	-0.95	0.18	1.27E-05	36.24	3.25E-05
FOXN3	-0.95	0.18	1.28E-05	36.08	3.44E-05
JARID2	-1.06	0.21	1.29E-05	44.74	1.24E-06
LRFN5	-0.95	0.18	1.29E-05	36.45	3.01E-05
REC8	-0.95	0.19	1.29E-05	36.64	2.82E-05
MRPL19	0.95	0.19	1.30E-05	36.62	2.84E-05
PBX4	-0.95	0.19	1.30E-05	36.09	3.44E-05
GNAI1	0.94	0.18	1.32E-05	35.84	3.79E-05
SLC12A8	1.20	0.24	1.32E-05	55.00	2.25E-08
ZMAT1	0.95	0.19	1.32E-05	36.47	2.99E-05
TOP3B	-1.43	0.28	1.32E-05	70.07	5.00E-11
TOX	-0.94	0.18	1.33E-05	36.76	2.69E-05
CD58	0.94	0.19	1.35E-05	36.59	2.87E-05
HDHD1	0.94	0.18	1.35E-05	35.33	4.61E-05
SLC25A14	1.23	0.24	1.35E-05	56.66	1.17E-08
GLIPR2	0.94	0.19	1.37E-05	37.43	2.12E-05
RWDD2A	0.94	0.18	1.37E-05	35.94	3.64E-05
CDK1	-0.94	0.18	1.37E-05	35.88	3.73E-05
KIF21B	-0.94	0.18	1.37E-05	36.36	3.12E-05
NKAIN4	-0.94	0.19	1.38E-05	37.09	2.37E-05
TRIM56	0.94	0.18	1.41E-05	35.16	4.92E-05
VAV2	-0.94	0.19	1.44E-05	36.63	2.84E-05
MOB3B	0.94	0.19	1.47E-05	36.97	2.47E-05
PCYOX1	0.94	0.18	1.47E-05	34.97	5.31E-05
ABCB9	-0.94	0.18	1.47E-05	35.20	4.85E-05
LRRFIP1	0.95	0.19	1.48E-05	37.39	2.14E-05
MYCBPAP	0.94	0.19	1.48E-05	36.51	2.95E-05
TTC30A	1.15	0.23	1.48E-05	51.72	8.00E-08
CAMK2B	-1.06	0.21	1.48E-05	45.22	1.03E-06
NAPEPLD	-1.10	0.22	1.48E-05	46.72	5.84E-07
DFNA5	0.98	0.19	1.50E-05	39.80	8.41E-06
GSPT2	1.17	0.23	1.51E-05	52.45	6.04E-08
GFRA2	-0.94	0.18	1.53E-05	35.63	4.13E-05
RRAS2	1.25	0.25	1.54E-05	57.21	9.37E-09
NEFL	0.94	0.18	1.58E-05	36.47	2.99E-05
CIR1	0.93	0.18	1.62E-05	34.61	6.07E-05
CDYL	0.94	0.19	1.68E-05	36.68	2.78E-05
CCND2	0.93	0.18	1.69E-05	36.15	3.37E-05

SULF2	0.93	0.18	1.69E-05	35.36	4.56E-05
EIF2B5	-0.93	0.18	1.70E-05	33.78	8.26E-05
ROBO4	-0.93	0.18	1.71E-05	34.33	6.71E-05
TTC1	0.93	0.19	1.72E-05	35.06	5.11E-05
ATF4	-0.93	0.18	1.72E-05	34.87	5.51E-05
TMCO4	0.93	0.19	1.73E-05	35.60	4.17E-05
HKDC1	0.93	0.18	1.80E-05	35.53	4.28E-05
RHBDD1	1.09	0.22	1.83E-05	46.71	5.86E-07
SYBU	1.34	0.27	1.83E-05	66.21	2.36E-10
HMGXB4	-0.93	0.18	1.83E-05	34.68	5.90E-05
CYP4F12	-0.93	0.18	1.87E-05	35.27	4.72E-05
FBXW4P1	-0.93	0.19	1.90E-05	34.79	5.68E-05
CHST14	0.93	0.18	1.91E-05	34.32	6.73E-05
NDUFAB1	1.22	0.24	1.91E-05	54.77	2.47E-08
MRPL33	0.93	0.18	1.92E-05	33.91	7.89E-05
MTFP1	-0.93	0.19	1.94E-05	35.33	4.61E-05
KLC1	-0.93	0.18	1.95E-05	34.43	6.47E-05
DNAH1	0.93	0.18	1.96E-05	35.99	3.57E-05
ITGAV	0.93	0.18	1.96E-05	35.43	4.44E-05
RPS27L	0.93	0.18	1.96E-05	34.94	5.37E-05
SYAP1	1.23	0.25	1.96E-05	57.30	9.14E-09
TAF6	0.93	0.18	1.96E-05	34.33	6.71E-05
UBE2A	1.00	0.20	1.97E-05	40.28	7.01E-06
ASTN2	0.92	0.18	1.98E-05	34.76	5.74E-05
SLC25A43	0.93	0.18	1.98E-05	34.21	7.00E-05
KLF12	-0.93	0.18	1.98E-05	35.35	4.59E-05
GBAS	1.42	0.28	2.00E-05	71.52	2.91E-11
SUN2	-0.92	0.18	2.00E-05	34.15	7.17E-05
ZNF512B	-0.93	0.18	2.00E-05	34.23	6.96E-05
TMEM120B	0.93	0.18	2.01E-05	35.49	4.35E-05
DYNLL1	0.92	0.18	2.03E-05	34.56	6.16E-05
SNAI1	-0.92	0.18	2.03E-05	34.37	6.61E-05
TMEM51	-0.92	0.18	2.04E-05	35.27	4.72E-05
IFIT5	0.92	0.18	2.05E-05	33.98	7.67E-05
PRR14L	-0.92	0.18	2.05E-05	33.99	7.65E-05
XBP1	1.05	0.21	2.06E-05	44.52	1.35E-06
PEX3	0.95	0.19	2.08E-05	37.01	2.44E-05
AP1G2	-0.92	0.18	2.09E-05	33.94	7.79E-05
OSBP2	-0.92	0.18	2.09E-05	34.39	6.56E-05
ACSM5	0.92	0.18	2.15E-05	34.24	6.94E-05
RRP1	-0.92	0.18	2.15E-05	32.77	1.21E-04
TTC13	-0.92	0.18	2.15E-05	34.67	5.94E-05
UBE2C	-1.08	0.22	2.15E-05	45.57	9.05E-07
GYG2	0.92	0.18	2.18E-05	34.59	6.10E-05
LRRC28	0.92	0.18	2.20E-05	34.46	6.38E-05
TMEM17	1.21	0.24	2.20E-05	56.09	1.48E-08
HIP1R	-0.92	0.18	2.22E-05	34.79	5.68E-05
GIMAP2	0.92	0.18	2.26E-05	35.78	3.87E-05
TP53TG1	0.92	0.18	2.27E-05	34.48	6.37E-05
PSMA2	0.92	0.18	2.30E-05	33.97	7.69E-05
SPTAN1	0.92	0.18	2.30E-05	34.69	5.90E-05
HFE	0.92	0.18	2.32E-05	34.86	5.53E-05
SUMF2	0.92	0.18	2.36E-05	34.27	6.85E-05
ADRB2	-1.18	0.24	2.36E-05	53.06	4.76E-08
ZNF133	-0.92	0.18	2.38E-05	33.43	9.35E-05
SELL	1.18	0.24	2.39E-05	54.13	3.16E-08
MRPL32	0.92	0.18	2.41E-05	34.61	6.06E-05
GRIA1	0.92	0.18	2.42E-05	35.68	4.04E-05

PPAT	-0.92	0.18	2.42E-05	34.58	6.13E-05
ZNF581	-0.92	0.18	2.42E-05	32.94	1.13E-04
FANCA	-0.92	0.18	2.43E-05	34.75	5.76E-05
PON3	1.19	0.24	2.44E-05	55.23	2.08E-08
HHIPL1	-0.91	0.18	2.48E-05	35.06	5.12E-05
DYRK4	0.91	0.18	2.49E-05	33.11	1.06E-04
TMTC4	-0.91	0.18	2.52E-05	35.69	4.02E-05
STARD8	-0.91	0.18	2.57E-05	33.79	8.23E-05
FAM71F1	0.91	0.18	2.58E-05	33.61	8.79E-05
PCBD1	1.41	0.28	2.58E-05	70.90	3.62E-11
WDR54	0.91	0.18	2.58E-05	34.47	6.38E-05
IL17RA	-1.02	0.21	2.58E-05	42.21	3.33E-06
OSBPL9	1.13	0.23	2.59E-05	49.74	1.78E-07
RAD54L2	-0.91	0.18	2.59E-05	33.21	1.02E-04
C9orf9	1.22	0.25	2.62E-05	56.96	1.04E-08
ABCD2	0.91	0.18	2.64E-05	34.50	6.32E-05
MPP7	0.91	0.18	2.69E-05	34.90	5.45E-05
RORB	-0.91	0.18	2.69E-05	34.90	5.45E-05
ULK2	0.91	0.18	2.70E-05	33.54	8.99E-05
AXIN2	-1.24	0.25	2.73E-05	57.27	9.17E-09
CCDC136	0.93	0.19	2.80E-05	37.00	2.45E-05
CITED2	0.91	0.18	2.83E-05	33.83	8.12E-05
FRMD3	1.45	0.29	2.83E-05	74.17	9.53E-12
FAM155B	-1.03	0.21	2.88E-05	42.89	2.57E-06
WNT16	1.26	0.26	2.91E-05	59.00	4.71E-09
ANTXR1	1.02	0.21	3.00E-05	42.48	3.01E-06
ARNTL2	0.91	0.18	3.00E-05	33.76	8.32E-05
FLNB	0.90	0.18	3.00E-05	33.50	9.14E-05
BRD1	-1.21	0.25	3.00E-05	52.91	5.02E-08
KLHL25	-0.91	0.18	3.00E-05	33.58	8.87E-05
SLC24A3	-0.91	0.18	3.02E-05	34.23	6.95E-05
GOLPH3	0.96	0.20	3.04E-05	37.32	2.20E-05
GPR37L1	-0.90	0.18	3.06E-05	33.58	8.87E-05
PDZD2	-0.90	0.18	3.07E-05	33.63	8.72E-05
ITGB5	0.90	0.18	3.08E-05	34.08	7.37E-05
LPAR2	-0.90	0.18	3.11E-05	32.99	1.11E-04
CIB1	0.90	0.18	3.14E-05	32.44	1.36E-04
PCCA	-1.24	0.25	3.14E-05	57.29	9.14E-09
C1orf198	-0.92	0.19	3.19E-05	36.42	3.03E-05
CA12	-0.90	0.18	3.19E-05	34.24	6.94E-05
CA2	-0.90	0.18	3.25E-05	33.36	9.62E-05
TFE3	0.90	0.18	3.27E-05	33.82	8.16E-05
ATP13A4	-0.90	0.18	3.31E-05	34.09	7.34E-05
FSTL4	-0.90	0.18	3.31E-05	34.35	6.67E-05
ITPK1	-1.05	0.21	3.31E-05	44.11	1.60E-06
MKNK2	-1.14	0.23	3.31E-05	49.68	1.81E-07
PCK2	-0.90	0.18	3.31E-05	33.03	1.10E-04
SYMPK	-0.92	0.19	3.31E-05	34.88	5.51E-05
FRY	0.91	0.19	3.32E-05	35.19	4.86E-05
PSMC2	0.90	0.18	3.33E-05	32.00	1.59E-04
TUFM	1.12	0.23	3.35E-05	47.29	4.71E-07
PARN	1.13	0.23	3.45E-05	48.85	2.51E-07
VMP1	0.90	0.18	3.45E-05	35.15	4.94E-05
INTS7	-0.90	0.18	3.45E-05	33.33	9.71E-05
DIRAS3	1.48	0.30	3.48E-05	79.07	1.15E-12
TSC22D2	-0.90	0.18	3.50E-05	33.81	8.18E-05
KATNB1	0.90	0.18	3.54E-05	34.15	7.18E-05
MCF2L	-0.90	0.18	3.58E-05	34.40	6.54E-05

SMEK2	0.90	0.18	3.61E-05	33.62	8.75E-05
UBL4A	0.90	0.18	3.61E-05	31.67	1.80E-04
STX17	1.18	0.24	3.66E-05	52.74	5.36E-08
SLC22A3	-0.90	0.18	3.68E-05	33.62	8.74E-05
IL18	0.89	0.18	3.75E-05	32.91	1.14E-04
ADIPOR1	0.89	0.18	3.81E-05	33.36	9.59E-05
CETN2	1.04	0.21	3.81E-05	44.13	1.58E-06
PHAX	0.94	0.19	3.81E-05	36.34	3.13E-05
STK40	-0.89	0.18	3.81E-05	32.14	1.52E-04
SF3B14	0.90	0.18	3.84E-05	33.47	9.22E-05
NAB1	1.01	0.21	3.91E-05	41.49	4.35E-06
MAP3K11	-0.92	0.19	3.91E-05	35.22	4.82E-05
KCNJ13	1.44	0.30	4.09E-05	75.10	6.36E-12
PHF8	0.89	0.18	4.14E-05	31.79	1.72E-04
RSPO2	-0.89	0.18	4.15E-05	34.43	6.47E-05
CCDC135	0.89	0.18	4.16E-05	33.79	8.22E-05
TRDMT1	0.89	0.18	4.23E-05	32.79	1.20E-04
PKD1L2	-0.89	0.18	4.26E-05	32.25	1.46E-04
RTP4	0.89	0.18	4.27E-05	33.75	8.34E-05
PARP16	1.24	0.26	4.30E-05	57.91	7.39E-09
LAMA2	-0.89	0.18	4.31E-05	32.58	1.30E-04
CD99L2	0.89	0.18	4.32E-05	32.34	1.41E-04
PKIA	-1.08	0.22	4.32E-05	46.35	6.76E-07
DDX5	0.89	0.18	4.33E-05	31.86	1.68E-04
TMEM65	0.94	0.19	4.33E-05	37.08	2.38E-05
INADL	1.18	0.25	4.34E-05	54.51	2.70E-08
AP4B1	-1.03	0.21	4.50E-05	39.77	8.49E-06
CTXN1	0.89	0.18	4.51E-05	32.50	1.33E-04
ARF5	0.89	0.18	4.63E-05	32.18	1.50E-04
MYLK	-0.88	0.18	4.66E-05	32.52	1.33E-04
MRVI1	-0.88	0.18	4.71E-05	33.06	1.08E-04
VPS13D	-0.88	0.18	4.71E-05	32.04	1.57E-04
WNT11	-0.88	0.18	4.75E-05	32.45	1.35E-04
HOXA3	0.88	0.18	4.79E-05	32.65	1.26E-04
KRCC1	0.88	0.18	4.82E-05	32.89	1.15E-04
PFKL	-0.88	0.18	4.85E-05	33.16	1.04E-04
DERL1	0.88	0.18	5.02E-05	32.12	1.53E-04
NDUFB6	1.24	0.26	5.02E-05	58.47	5.87E-09
BOC	-0.88	0.18	5.02E-05	32.17	1.50E-04
SVIL	1.09	0.23	5.06E-05	46.89	5.49E-07
MPDZ	1.42	0.30	5.14E-05	73.12	1.48E-11
DNAAF1	0.94	0.20	5.15E-05	37.82	1.84E-05
BCL7B	1.17	0.24	5.21E-05	52.23	6.52E-08
PIM2	0.88	0.18	5.45E-05	31.39	1.99E-04
TCF7L2	0.96	0.20	5.45E-05	38.60	1.35E-05
BHLHE41	0.88	0.18	5.57E-05	32.92	1.14E-04
IGBP1	0.88	0.18	5.60E-05	32.05	1.56E-04
B4GALT4	0.88	0.18	5.62E-05	31.39	1.99E-04
HAPLN1	-0.88	0.18	5.67E-05	33.00	1.11E-04
YIPF6	0.88	0.18	5.75E-05	32.03	1.58E-04
ANK2	0.88	0.18	5.77E-05	32.12	1.53E-04
PTH1R	-0.88	0.18	5.78E-05	33.48	9.19E-05
GPKOW	0.91	0.19	5.83E-05	35.07	5.11E-05
SCRIB	-0.88	0.18	5.83E-05	32.72	1.23E-04
BIRC5	-0.87	0.18	5.91E-05	31.63	1.82E-04
LYG1	-0.87	0.18	5.91E-05	30.74	2.53E-04
CHST9	0.98	0.21	6.02E-05	40.18	7.25E-06
KCTD20	-0.87	0.18	6.15E-05	31.67	1.79E-04

ADORA2B	-0.87	0.18	6.23E-05	31.92	1.64E-04
KIAA1430	0.90	0.19	6.25E-05	34.62	6.05E-05
ANG	0.87	0.18	6.26E-05	31.60	1.84E-04
PYCR2	0.87	0.18	6.31E-05	31.11	2.21E-04
HELLS	-0.87	0.18	6.37E-05	31.83	1.70E-04
PPA2	0.91	0.19	6.50E-05	35.33	4.61E-05
C5	0.87	0.18	6.55E-05	32.13	1.53E-04
SOCS2	1.26	0.27	6.57E-05	60.46	2.67E-09
NIPSNAP3B	1.00	0.21	6.58E-05	41.20	4.91E-06
FBLN5	0.87	0.18	6.60E-05	31.43	1.96E-04
PACRG	0.87	0.18	6.80E-05	32.50	1.33E-04
ATAD2	-0.87	0.18	6.80E-05	32.96	1.13E-04
VAMP5	0.87	0.18	6.88E-05	32.06	1.56E-04
SLC9A6	0.87	0.18	6.91E-05	31.53	1.89E-04
FNBP4	-0.87	0.18	6.91E-05	31.16	2.17E-04
PACSIN3	-0.87	0.18	7.03E-05	31.42	1.97E-04
CSNK2A2	0.87	0.18	7.07E-05	30.48	2.77E-04
DCK	0.87	0.18	7.14E-05	32.20	1.49E-04
FAM160B1	0.86	0.18	7.14E-05	30.10	3.17E-04
GAS8	0.87	0.18	7.14E-05	32.16	1.51E-04
POLR2J	0.87	0.18	7.14E-05	30.96	2.33E-04
UXT	0.87	0.18	7.14E-05	32.06	1.56E-04
CCDC114	0.87	0.18	7.20E-05	32.46	1.35E-04
TNFAIP1	0.86	0.18	7.20E-05	30.38	2.87E-04
RECQL4	-0.86	0.18	7.24E-05	30.56	2.68E-04
STARD3	-0.86	0.18	7.33E-05	30.70	2.57E-04
SAMD9L	0.86	0.18	7.35E-05	31.01	2.30E-04
MSX2	1.04	0.22	7.41E-05	44.15	1.57E-06
RIMKLA	0.89	0.19	7.43E-05	33.80	8.18E-05
DCUN1D2	-0.98	0.21	7.45E-05	39.39	9.89E-06
DPCD	1.02	0.22	7.46E-05	43.01	2.45E-06
GLG1	0.86	0.18	7.55E-05	30.89	2.40E-04
SPARCL1	0.86	0.18	7.55E-05	31.95	1.62E-04
HSPA1L	0.93	0.20	7.64E-05	36.39	3.07E-05
BTBD11	-0.86	0.18	7.64E-05	32.09	1.54E-04
ZNF692	-0.86	0.18	7.64E-05	31.11	2.21E-04
MDFIC	1.18	0.25	7.66E-05	54.54	2.69E-08
ALG2	0.86	0.18	7.76E-05	31.32	2.04E-04
EZR	1.13	0.24	7.78E-05	50.87	1.14E-07
PRDX6	1.05	0.23	7.82E-05	44.60	1.31E-06
CDCA4	-0.86	0.18	7.82E-05	30.46	2.78E-04
SLU7	0.86	0.18	7.84E-05	30.81	2.47E-04
TUFT1	0.86	0.18	7.84E-05	31.69	1.79E-04
CRIP1	1.03	0.22	8.15E-05	43.94	1.71E-06
MGLL	-0.86	0.18	8.20E-05	31.42	1.97E-04
BNC2	0.86	0.18	8.33E-05	31.82	1.70E-04
ODF3L1	0.86	0.18	8.33E-05	30.57	2.68E-04
INPP4A	0.86	0.18	8.34E-05	30.67	2.59E-04
NUDT2	0.86	0.18	8.37E-05	30.59	2.67E-04
TMEM231	0.86	0.18	8.41E-05	32.02	1.58E-04
TSPAN2	0.86	0.18	8.41E-05	31.93	1.63E-04
KIAA1217	0.86	0.18	8.44E-05	30.99	2.31E-04
PDLIM3	0.86	0.18	8.51E-05	32.20	1.49E-04
PSMD6	0.86	0.18	8.51E-05	30.45	2.79E-04
JMJD8	0.86	0.18	8.53E-05	31.28	2.07E-04
STAR	0.86	0.18	8.65E-05	31.23	2.11E-04
NCAPG	-0.85	0.18	8.72E-05	30.72	2.55E-04
GOLGA1	0.88	0.19	8.81E-05	32.78	1.20E-04

TNFSF4	0.85	0.18	8.86E-05	30.85	2.43E-04
TIFA	-0.85	0.18	8.86E-05	30.60	2.66E-04
B3GNT5	0.92	0.20	8.94E-05	36.21	3.29E-05
FAM83D	-1.02	0.22	8.94E-05	42.95	2.50E-06
ENPP4	0.85	0.18	8.98E-05	31.22	2.12E-04
ARMCX3	0.85	0.18	9.03E-05	30.93	2.36E-04
C10orf107	0.96	0.21	9.06E-05	39.44	9.71E-06
C6orf57	0.85	0.18	9.10E-05	30.57	2.68E-04
KLHDC9	0.85	0.18	9.15E-05	31.23	2.11E-04
C16orf89	-0.85	0.18	9.15E-05	30.92	2.37E-04
GALK2	1.41	0.30	9.18E-05	67.75	1.27E-10
LGI4	-0.85	0.18	9.19E-05	30.57	2.68E-04
NUSAP1	-0.85	0.18	9.19E-05	30.51	2.74E-04
GPSM1	-0.85	0.18	9.25E-05	32.20	1.49E-04
MAMDC2	0.85	0.18	9.34E-05	30.74	2.54E-04
TTC16	0.85	0.18	9.39E-05	30.41	2.83E-04
ATP6AP2	0.85	0.18	9.42E-05	31.67	1.79E-04
GATAD1	0.85	0.18	9.42E-05	29.04	4.67E-04
FGF9	-0.85	0.18	9.52E-05	32.05	1.56E-04
TTC23L	0.99	0.21	9.71E-05	40.80	5.69E-06
PPIL3	0.85	0.18	9.72E-05	29.59	3.85E-04
SERTAD4	0.85	0.18	9.72E-05	31.78	1.72E-04
PHKA1	0.85	0.18	9.82E-05	30.18	3.07E-04
SYTL2	1.60	0.35	9.83E-05	84.84	9.84E-14
CLCN2	-0.85	0.18	9.85E-05	29.17	4.47E-04
PLTP	0.85	0.18	1.01E-04	30.61	2.65E-04
NUDT10	0.85	0.18	1.01E-04	30.23	3.03E-04
DNAJC19	0.85	0.18	1.02E-04	30.83	2.45E-04
HSPA12A	0.85	0.18	1.02E-04	31.71	1.77E-04
CLPX	0.85	0.18	1.03E-04	30.78	2.49E-04
MORF4L2	0.85	0.18	1.05E-04	31.29	2.07E-04
ARHGEF2	-1.01	0.22	1.05E-04	42.15	3.39E-06
SPARC	0.85	0.18	1.06E-04	31.72	1.76E-04
ATOX1	0.85	0.18	1.06E-04	30.15	3.12E-04
DNAI1	0.85	0.18	1.07E-04	30.67	2.59E-04
BMPR1B	-1.11	0.24	1.07E-04	48.87	2.50E-07
ATP1A2	-0.84	0.18	1.07E-04	30.20	3.07E-04
ACOT13	1.04	0.23	1.08E-04	43.31	2.19E-06
SLC6A13	-0.84	0.18	1.08E-04	30.18	3.07E-04
HHAT	0.84	0.18	1.09E-04	29.79	3.57E-04
TAC3	-0.84	0.18	1.09E-04	30.20	3.07E-04
TMEM138	0.84	0.18	1.09E-04	30.87	2.41E-04
RAP2B	-0.84	0.18	1.10E-04	29.64	3.77E-04
RGS11	-0.84	0.18	1.11E-04	30.67	2.59E-04
RNGTT	1.01	0.22	1.11E-04	42.49	3.01E-06
NDUFB3	1.09	0.24	1.12E-04	45.92	7.92E-07
VAMP3	0.96	0.21	1.12E-04	38.89	1.21E-05
ESPL1	-0.84	0.18	1.13E-04	29.15	4.51E-04
VIPR2	-1.03	0.23	1.17E-04	43.72	1.86E-06
PANK4	-0.84	0.18	1.17E-04	30.23	3.03E-04
SLC1A5	-0.84	0.18	1.19E-04	30.68	2.59E-04
NUP210	-0.84	0.18	1.19E-04	30.49	2.76E-04
NIT2	0.84	0.18	1.21E-04	29.70	3.70E-04
KDELR3	0.84	0.18	1.21E-04	30.61	2.65E-04
XK	1.25	0.27	1.23E-04	59.51	3.84E-09
TC2N	0.90	0.20	1.24E-04	35.29	4.69E-05
PXMP2	-1.36	0.30	1.24E-04	64.93	4.10E-10
RCE1	-0.84	0.18	1.24E-04	28.31	6.08E-04

ARHGEF25	-0.89	0.19	1.26E-04	33.81	8.18E-05
PTPN3	0.84	0.18	1.28E-04	31.05	2.26E-04
LYPD1	0.84	0.18	1.29E-04	30.86	2.43E-04
TIMP4	0.84	0.18	1.29E-04	29.92	3.38E-04
C12orf60	0.98	0.21	1.31E-04	39.48	9.57E-06
SLC44A1	0.84	0.18	1.31E-04	30.62	2.65E-04
CPPED1	0.83	0.18	1.35E-04	30.73	2.55E-04
CHFR	-0.83	0.18	1.36E-04	28.92	4.89E-04
CYLD	0.83	0.18	1.37E-04	30.36	2.88E-04
ERP44	0.83	0.18	1.39E-04	29.35	4.19E-04
PLXNA1	0.83	0.18	1.39E-04	30.06	3.22E-04
FKBP14	-0.83	0.18	1.39E-04	29.73	3.65E-04
NDC80	-0.83	0.18	1.40E-04	29.48	4.00E-04
FAM174A	1.07	0.24	1.41E-04	46.56	6.21E-07
DDIT4	-1.07	0.24	1.41E-04	46.41	6.59E-07
PPP1R32	0.90	0.20	1.42E-04	35.48	4.36E-05
MTFMT	1.18	0.26	1.43E-04	54.07	3.23E-08
ZNF276	-0.83	0.18	1.43E-04	28.90	4.91E-04
DIRAS1	-0.83	0.18	1.46E-04	29.23	4.39E-04
CCDC125	0.83	0.18	1.47E-04	30.62	2.64E-04
CCDC34	1.07	0.24	1.47E-04	46.81	5.64E-07
ANKRD29	1.04	0.23	1.47E-04	44.93	1.16E-06
KIAA1244	-0.83	0.18	1.49E-04	30.33	2.91E-04
BUB1B	-0.83	0.18	1.51E-04	29.15	4.50E-04
WWC1	0.83	0.18	1.53E-04	29.81	3.55E-04
ZNF335	-0.83	0.18	1.53E-04	27.42	8.46E-04
PAPD5	1.50	0.33	1.55E-04	79.24	1.09E-12
URB1	0.83	0.18	1.55E-04	29.18	4.46E-04
ATP2B2	-1.02	0.23	1.56E-04	43.27	2.23E-06
GRK5	-0.83	0.18	1.56E-04	29.05	4.67E-04
SACS	-0.83	0.18	1.56E-04	29.69	3.71E-04
CREBL2	1.06	0.24	1.57E-04	45.99	7.75E-07
BID	-0.83	0.18	1.57E-04	30.48	2.77E-04
DPF1	-0.83	0.18	1.57E-04	28.44	5.80E-04
SLC47A1	-0.83	0.18	1.57E-04	29.14	4.52E-04
HDAC3	0.83	0.18	1.61E-04	29.05	4.65E-04
NT5E	0.82	0.18	1.61E-04	28.93	4.87E-04
LRRFIP2	0.83	0.18	1.61E-04	30.33	2.91E-04
DRD1	1.49	0.33	1.62E-04	79.26	1.09E-12
TCEAL8	0.86	0.19	1.62E-04	32.14	1.52E-04
GSTM3	-0.82	0.18	1.62E-04	29.19	4.45E-04
STAMBPL1	0.82	0.18	1.62E-04	28.88	4.95E-04
ITFG1	0.82	0.18	1.63E-04	28.90	4.92E-04
RBBP7	0.82	0.18	1.63E-04	28.94	4.86E-04
TXNRD2	-0.84	0.19	1.64E-04	29.96	3.34E-04
JAM3	1.08	0.24	1.64E-04	45.69	8.67E-07
DERA	0.82	0.18	1.66E-04	29.73	3.65E-04
CLDN10	-0.82	0.18	1.66E-04	29.12	4.55E-04
H2AFY2	-0.82	0.18	1.67E-04	28.81	5.08E-04
DOK5	-0.82	0.18	1.68E-04	29.92	3.38E-04
C1orf101	1.07	0.24	1.69E-04	46.88	5.49E-07
KCNJ10	-0.82	0.18	1.69E-04	29.20	4.44E-04
ZC4H2	0.82	0.18	1.70E-04	29.25	4.34E-04
CD24	1.17	0.26	1.70E-04	54.17	3.14E-08
OLFML2A	0.82	0.18	1.70E-04	30.03	3.26E-04
CALM3	-0.82	0.18	1.71E-04	29.31	4.26E-04
SEPT8	-0.82	0.18	1.71E-04	29.97	3.34E-04
KLHDC8A	1.36	0.30	1.72E-04	69.28	6.93E-11

SEMA3A	1.15	0.26	1.73E-04	52.18	6.62E-08
KAT2A	-0.82	0.18	1.76E-04	28.12	6.53E-04
GNAL	-0.82	0.18	1.77E-04	29.27	4.33E-04
ALPK1	-0.82	0.18	1.77E-04	29.28	4.30E-04
FIS1	0.82	0.18	1.77E-04	29.42	4.09E-04
B4GALNT1	-0.82	0.18	1.77E-04	30.08	3.19E-04
RAB14	0.82	0.18	1.78E-04	29.42	4.09E-04
RFK	1.20	0.27	1.78E-04	53.54	3.98E-08
DAGLA	-0.82	0.18	1.78E-04	28.49	5.70E-04
ZWINT	-0.82	0.18	1.78E-04	28.94	4.85E-04
IRS1	0.82	0.18	1.81E-04	29.06	4.64E-04
PRDM11	-0.82	0.18	1.84E-04	28.49	5.69E-04
IFLTD1	0.82	0.18	1.86E-04	29.51	3.96E-04
ABCG1	-0.82	0.18	1.86E-04	28.50	5.67E-04
TEKT1	0.89	0.20	1.87E-04	34.47	6.38E-05
GRAMD1B	-0.82	0.18	1.87E-04	29.58	3.86E-04
ZSCAN22	-1.06	0.24	1.90E-04	43.16	2.32E-06
TFB1M	0.82	0.18	1.91E-04	28.20	6.36E-04
TOP2A	-0.82	0.18	1.91E-04	28.98	4.79E-04
UNC93B1	0.82	0.18	1.91E-04	29.01	4.73E-04
THEM4	0.82	0.18	1.92E-04	28.34	6.02E-04
UBE2H	0.81	0.18	1.95E-04	28.55	5.58E-04
EFHC1	0.82	0.19	1.96E-04	30.45	2.79E-04
CDC20	-0.81	0.18	1.97E-04	28.29	6.12E-04
SLC26A10	-0.81	0.18	1.97E-04	28.90	4.91E-04
CDC34	-0.91	0.21	2.00E-04	35.10	5.05E-05
PIK3R3	-1.18	0.27	2.01E-04	53.90	3.44E-08
IARS2	0.81	0.18	2.01E-04	28.58	5.55E-04
NKAP	0.98	0.22	2.02E-04	39.99	7.83E-06
EPHB2	-0.81	0.18	2.03E-04	28.78	5.14E-04
MTSS1	-0.81	0.18	2.07E-04	28.84	5.03E-04
TNFRSF11B	-0.81	0.18	2.08E-04	28.90	4.91E-04
CENPQ	0.81	0.18	2.09E-04	28.14	6.50E-04
SYNGR1	-0.81	0.18	2.09E-04	28.80	5.12E-04
IP6K3	1.08	0.25	2.11E-04	47.38	4.54E-07
SNTB1	0.84	0.19	2.11E-04	31.45	1.94E-04
NME9	0.81	0.18	2.14E-04	28.72	5.25E-04
EHD1	-0.96	0.22	2.14E-04	38.33	1.50E-05
BBOX1	0.81	0.18	2.15E-04	29.40	4.11E-04
YPEL2	0.81	0.18	2.15E-04	28.38	5.95E-04
NRF1	0.81	0.18	2.16E-04	27.34	8.69E-04
FUNDC1	0.81	0.18	2.16E-04	27.40	8.51E-04
PDGFC	0.81	0.18	2.16E-04	29.77	3.60E-04
PSD	-0.81	0.18	2.16E-04	27.72	7.59E-04
C20orf96	0.81	0.18	2.17E-04	29.50	3.97E-04
LIPG	0.81	0.18	2.17E-04	29.91	3.40E-04
MKI67	-0.81	0.18	2.17E-04	29.56	3.89E-04
PPP2CB	0.81	0.18	2.18E-04	27.98	6.89E-04
ASXL2	1.07	0.24	2.18E-04	46.41	6.59E-07
CDKL1	0.97	0.22	2.19E-04	39.00	1.16E-05
ISCU	1.15	0.26	2.19E-04	52.33	6.31E-08
NDUFS6	0.81	0.18	2.19E-04	27.33	8.73E-04
SLC29A4	-0.89	0.20	2.19E-04	34.07	7.39E-05
DOCK7	0.81	0.18	2.20E-04	27.88	7.14E-04
WDR52	0.81	0.18	2.21E-04	28.50	5.68E-04
TMTC2	0.81	0.18	2.22E-04	29.12	4.55E-04
GPC4	0.81	0.18	2.22E-04	28.71	5.27E-04
ANO10	-0.81	0.18	2.24E-04	28.85	5.02E-04

MANSC1	0.86	0.20	2.29E-04	32.66	1.26E-04
TMEM14C	0.84	0.19	2.32E-04	31.06	2.25E-04
SS18L1	-0.81	0.18	2.33E-04	27.26	8.91E-04
MYL3	-0.81	0.18	2.34E-04	28.69	5.32E-04
C5orf55	0.81	0.18	2.38E-04	27.83	7.29E-04
LINC00311	-0.80	0.18	2.39E-04	26.25	1.27E-03
SGPP2	1.75	0.40	2.39E-04	102.86	0.00E+00
ENTPD5	-0.81	0.18	2.39E-04	27.94	6.98E-04
ANKRD7	1.10	0.25	2.40E-04	49.00	2.39E-07
MYO5C	0.93	0.21	2.40E-04	37.22	2.28E-05
TMEM106B	0.80	0.18	2.42E-04	27.45	8.36E-04
INSR	-0.80	0.18	2.44E-04	27.77	7.46E-04
CCDC85B	-0.80	0.18	2.45E-04	28.74	5.23E-04
TSR2	0.80	0.18	2.45E-04	27.70	7.63E-04
TTYH2	-0.80	0.18	2.45E-04	28.53	5.62E-04
MED15	-0.80	0.18	2.46E-04	28.22	6.29E-04
PPM1B	0.80	0.18	2.47E-04	27.49	8.23E-04
NGFRAP1	0.80	0.18	2.48E-04	27.78	7.42E-04
DUSP1	0.80	0.18	2.50E-04	29.65	3.77E-04
PTP4A3	-0.80	0.18	2.50E-04	28.14	6.50E-04
SPAG8	0.80	0.18	2.52E-04	27.99	6.86E-04
DOLK	0.80	0.18	2.52E-04	27.33	8.73E-04
COQ4	0.98	0.23	2.53E-04	40.07	7.55E-06
IK	1.06	0.24	2.53E-04	46.19	7.14E-07
ACSS3	0.80	0.18	2.53E-04	28.51	5.66E-04
NRXN2	-0.80	0.18	2.53E-04	27.58	7.97E-04
HP1BP3	-0.80	0.18	2.55E-04	27.71	7.60E-04
GPR39	0.80	0.18	2.56E-04	28.26	6.20E-04
AK1	0.80	0.18	2.57E-04	28.88	4.95E-04
SPAG6	0.80	0.18	2.57E-04	28.93	4.87E-04
KIAA0319	0.80	0.18	2.60E-04	28.35	5.99E-04
ADAMTSL3	-0.80	0.18	2.62E-04	27.74	7.54E-04
CYB5R1	0.80	0.18	2.65E-04	27.65	7.76E-04
INHA	0.80	0.18	2.68E-04	27.28	8.87E-04
CCDC25	0.80	0.18	2.69E-04	27.50	8.20E-04
PDK4	0.80	0.18	2.69E-04	29.14	4.52E-04
SERPINB6	1.17	0.27	2.71E-04	53.38	4.23E-08
MYLK2	-0.80	0.18	2.72E-04	28.29	6.12E-04
MOV10	-0.80	0.18	2.72E-04	27.11	9.41E-04
SPCS1	0.80	0.18	2.73E-04	27.44	8.39E-04
RPS6KA3	0.80	0.19	2.75E-04	29.31	4.25E-04
TCF4	-0.90	0.21	2.77E-04	35.22	4.82E-05
CDC7	-0.80	0.18	2.77E-04	27.99	6.85E-04
LINC00260	-0.83	0.19	2.78E-04	30.38	2.86E-04
NELL2	0.82	0.19	2.79E-04	30.57	2.68E-04
SPTBN4	-0.80	0.18	2.80E-04	27.07	9.53E-04
TTC18	0.80	0.18	2.81E-04	28.74	5.22E-04
NSRP1	0.80	0.18	2.84E-04	28.29	6.13E-04
BUD31	1.01	0.23	2.84E-04	41.90	3.75E-06
ACN9	0.88	0.20	2.85E-04	33.83	8.12E-05
NUMBL	-0.80	0.18	2.87E-04	26.70	1.08E-03
PNPLA3	-0.80	0.18	2.88E-04	28.68	5.34E-04
NSUN7	0.89	0.21	2.89E-04	34.83	5.59E-05
GADD45G	-0.79	0.18	2.94E-04	28.07	6.67E-04
YIPF1	0.79	0.18	2.95E-04	26.70	1.08E-03
RALGDS	-0.79	0.18	2.95E-04	28.31	6.08E-04
IVNS1ABP	-0.79	0.18	2.95E-04	28.27	6.16E-04
MRPS33	1.22	0.28	2.97E-04	56.10	1.48E-08

SCPEP1	0.79	0.18	2.99E-04	27.17	9.22E-04
TCF3	-0.79	0.18	2.99E-04	26.48	1.17E-03
EMP2	0.79	0.18	3.02E-04	28.82	5.07E-04
PSMB1	0.79	0.18	3.05E-04	26.80	1.05E-03
MCM3AP-AS1	-0.79	0.18	3.05E-04	27.11	9.40E-04
SHISA4	-0.79	0.18	3.08E-04	27.94	6.98E-04
SSR2	-0.92	0.21	3.11E-04	36.57	2.89E-05
SYTL4	0.79	0.18	3.13E-04	27.31	8.75E-04
LPAR6	0.79	0.18	3.14E-04	26.98	9.81E-04
RPUSD4	-0.83	0.19	3.16E-04	29.80	3.56E-04
PPP1CB	0.79	0.18	3.16E-04	26.73	1.07E-03
STRA13	-0.79	0.18	3.17E-04	26.71	1.08E-03
LMNB1	-0.79	0.18	3.18E-04	27.22	9.03E-04
SLC22A4	1.01	0.24	3.20E-04	43.40	2.11E-06
GPR144	0.79	0.18	3.20E-04	27.16	9.24E-04
DLL4	-0.93	0.22	3.20E-04	36.53	2.93E-05
ZNF114	-0.79	0.18	3.20E-04	27.02	9.72E-04
GGT5	-0.79	0.18	3.21E-04	27.71	7.60E-04
NPTXR	-0.79	0.18	3.22E-04	27.96	6.93E-04
KCNAB3	-0.79	0.18	3.24E-04	27.68	7.67E-04
ITPR1	0.79	0.18	3.25E-04	27.01	9.73E-04
ANKRD37	0.82	0.19	3.28E-04	30.20	3.07E-04
SNX14	0.79	0.18	3.30E-04	27.34	8.68E-04
ZNF483	1.49	0.35	3.31E-04	78.77	1.26E-12
TRO	-0.79	0.18	3.33E-04	27.13	9.35E-04
TMEM107	1.22	0.28	3.33E-04	57.71	8.03E-09
CAPN1	-0.79	0.18	3.33E-04	27.12	9.37E-04
C1QBP	0.79	0.18	3.34E-04	26.35	1.23E-03
IL5RA	0.92	0.21	3.34E-04	36.85	2.61E-05
MGC10814	-0.79	0.18	3.34E-04	27.59	7.94E-04
KIF9	0.79	0.18	3.36E-04	28.40	5.89E-04
MFSD2A	0.79	0.18	3.37E-04	28.37	5.96E-04
UNC5C	-0.79	0.18	3.37E-04	27.81	7.35E-04
CCDC67	1.11	0.26	3.38E-04	49.97	1.63E-07
ASCC2	-0.79	0.18	3.38E-04	25.91	1.43E-03
GLYR1	0.79	0.18	3.39E-04	26.56	1.14E-03
ATP1B2	-0.79	0.18	3.40E-04	26.92	1.01E-03
DNAH7	0.86	0.20	3.46E-04	32.68	1.25E-04
TMEM116	0.99	0.23	3.47E-04	40.68	5.97E-06
CSRP2	0.78	0.18	3.47E-04	26.93	1.00E-03
SPEF1	0.90	0.21	3.49E-04	35.57	4.21E-05
CDCA7	-0.78	0.18	3.49E-04	27.04	9.63E-04
CENPM	-0.78	0.18	3.49E-04	26.30	1.25E-03
VPS35	1.16	0.27	3.49E-04	52.62	5.64E-08
MED31	0.78	0.18	3.49E-04	26.42	1.20E-03
WNT5B	-0.79	0.18	3.49E-04	27.66	7.75E-04
HDAC10	-0.79	0.18	3.50E-04	26.69	1.09E-03
ARMC6	-0.78	0.18	3.50E-04	25.64	1.57E-03
BPGM	0.78	0.18	3.50E-04	26.16	1.31E-03
SLC13A3	-0.78	0.18	3.52E-04	27.56	8.03E-04
TRIP10	-0.78	0.18	3.52E-04	27.37	8.59E-04
LRIG3	1.13	0.26	3.52E-04	51.69	8.12E-08
STYXL1	0.85	0.20	3.53E-04	31.61	1.84E-04
KCNJ3	-1.11	0.26	3.54E-04	48.17	3.30E-07
MAP6	0.78	0.18	3.54E-04	27.49	8.24E-04
CDH1	1.41	0.33	3.61E-04	71.07	3.50E-11
AKR1B1	0.78	0.18	3.61E-04	26.62	1.12E-03
BABAM1	-0.78	0.18	3.62E-04	26.91	1.01E-03

RHOJ	-0.78	0.18	3.63E-04	27.18	9.20E-04
TBC1D7	-1.00	0.23	3.63E-04	41.75	3.98E-06
SOD1	1.42	0.33	3.64E-04	71.18	3.39E-11
PIAS4	-0.78	0.18	3.65E-04	26.36	1.23E-03
ABI3BP	0.78	0.18	3.67E-04	26.73	1.07E-03
DSTN	1.06	0.25	3.72E-04	45.98	7.75E-07
IDH3A	1.15	0.27	3.72E-04	52.07	6.91E-08
CDC37L1	0.84	0.20	3.76E-04	31.35	2.02E-04
IQUB	1.23	0.29	3.76E-04	59.40	4.00E-09
RHOBTB2	-0.80	0.19	3.76E-04	29.31	4.25E-04
SPHK1	-0.78	0.18	3.76E-04	28.03	6.77E-04
GALC	0.81	0.19	3.79E-04	29.69	3.71E-04
CDH6	0.78	0.18	3.79E-04	28.55	5.58E-04
MAK	0.90	0.21	3.82E-04	35.50	4.33E-05
RNF13	0.78	0.18	3.83E-04	26.13	1.32E-03
ASAP1	-0.78	0.18	3.87E-04	26.13	1.32E-03
NRG1	0.86	0.20	3.88E-04	33.20	1.02E-04
PRUNE	1.12	0.26	3.88E-04	50.40	1.38E-07
HCAR1	-0.78	0.18	3.89E-04	26.50	1.17E-03
PAPSS2	-0.78	0.18	3.96E-04	27.02	9.71E-04
TRAPPC1	0.78	0.18	3.97E-04	26.99	9.80E-04
DCXR	-0.78	0.18	4.00E-04	27.90	7.10E-04
DDX49	-0.78	0.18	4.00E-04	26.26	1.27E-03
MED16	-0.78	0.18	4.00E-04	25.67	1.55E-03
DLEC1	0.98	0.23	4.01E-04	41.12	5.06E-06
ANO2	-0.78	0.18	4.03E-04	26.58	1.13E-03
PFKP	-0.78	0.18	4.05E-04	26.91	1.01E-03
TPX2	-0.78	0.18	4.05E-04	26.87	1.02E-03
GPR56	-0.78	0.18	4.07E-04	27.20	9.12E-04
ARHGAP11A	-0.78	0.18	4.10E-04	26.28	1.26E-03
HAPLN3	-0.78	0.18	4.17E-04	27.19	9.14E-04
COLQ	1.14	0.27	4.19E-04	52.23	6.52E-08
SELO	-0.88	0.21	4.19E-04	32.51	1.33E-04
SAMD14	-0.77	0.18	4.24E-04	26.78	1.05E-03
SPIN1	1.00	0.24	4.25E-04	41.90	3.75E-06
EFNB3	0.84	0.20	4.26E-04	31.79	1.72E-04
CHM	0.77	0.18	4.27E-04	25.95	1.41E-03
FASN	-0.77	0.18	4.31E-04	27.03	9.68E-04
MBOAT1	1.11	0.26	4.34E-04	48.53	2.87E-07
CD36	0.77	0.18	4.34E-04	27.04	9.63E-04
ALKBH5	0.77	0.18	4.40E-04	26.99	9.80E-04
SDC2	0.77	0.18	4.40E-04	27.72	7.59E-04
MAF	0.85	0.20	4.42E-04	32.01	1.58E-04
TWISTNB	0.96	0.23	4.47E-04	39.43	9.74E-06
GABRA2	-0.77	0.18	4.47E-04	26.65	1.10E-03
RAC1	0.77	0.18	4.48E-04	25.98	1.39E-03
ZNF23	0.98	0.23	4.48E-04	40.41	6.64E-06
PLAGL1	-0.77	0.18	4.49E-04	26.83	1.03E-03
SRRM4	-0.77	0.18	4.49E-04	26.33	1.24E-03
SEZ6L	-1.11	0.26	4.53E-04	48.78	2.59E-07
ANXA3	1.53	0.36	4.54E-04	78.95	1.20E-12
FAM104B	1.34	0.32	4.60E-04	67.03	1.70E-10
SLITRK1	-0.77	0.18	4.62E-04	26.42	1.20E-03
FOXA1	1.56	0.37	4.62E-04	86.44	3.67E-14
ANKRD45	0.80	0.19	4.63E-04	29.17	4.47E-04
FNBP1	-0.77	0.18	4.63E-04	27.24	8.99E-04
STON2	-0.77	0.18	4.63E-04	26.12	1.33E-03
CABIN1	-0.77	0.18	4.65E-04	25.74	1.51E-03

TAB2	0.77	0.18	4.66E-04	27.20	9.12E-04
DKK3	0.77	0.18	4.66E-04	26.04	1.37E-03
PRDM2	-0.77	0.18	4.66E-04	26.68	1.09E-03
ARMC2	0.77	0.18	4.69E-04	27.08	9.51E-04
MAPK11	-0.77	0.18	4.70E-04	25.33	1.75E-03
ZNF710	-0.77	0.18	4.70E-04	25.54	1.63E-03
ZNF267	0.77	0.18	4.73E-04	25.81	1.48E-03
HSPA9	0.77	0.18	4.74E-04	26.37	1.22E-03
SCP2	0.83	0.20	4.83E-04	30.98	2.32E-04
CKAP2	-0.77	0.18	4.83E-04	26.47	1.18E-03
BRSK1	-0.87	0.21	4.84E-04	32.81	1.19E-04
PCBP4	-0.77	0.18	4.85E-04	26.26	1.27E-03
ETF1	0.77	0.18	4.87E-04	26.18	1.30E-03
PLIN1	-0.77	0.18	4.88E-04	26.87	1.02E-03
CKLF	0.77	0.18	4.90E-04	26.06	1.35E-03
KRI1	-0.77	0.18	4.91E-04	26.34	1.23E-03
AK8	1.03	0.25	4.97E-04	44.78	1.23E-06
CATSPER3	0.77	0.18	4.97E-04	25.15	1.87E-03
PACS2	-0.77	0.18	4.98E-04	25.91	1.43E-03
METTL20	0.77	0.18	4.99E-04	26.24	1.28E-03
SLC7A5	-1.28	0.31	5.00E-04	61.90	1.41E-09
EXOC3L1	-0.77	0.18	5.00E-04	25.14	1.88E-03
LCA5	1.03	0.25	5.01E-04	44.58	1.32E-06
ALDH1L1	-0.77	0.18	5.01E-04	27.18	9.18E-04
HCFC2	0.77	0.18	5.02E-04	25.69	1.54E-03
TGM3	0.98	0.24	5.03E-04	41.08	5.13E-06
PIGK	0.77	0.18	5.04E-04	26.61	1.12E-03
PSMD7	0.76	0.18	5.12E-04	24.97	2.00E-03
PITPNA	1.04	0.25	5.13E-04	44.96	1.15E-06
SLC25A18	-0.76	0.18	5.15E-04	26.06	1.36E-03
ZFAND3	0.88	0.21	5.18E-04	33.48	9.19E-05
NMBR	1.01	0.24	5.20E-04	42.20	3.33E-06
CAMKK1	-0.76	0.18	5.22E-04	25.24	1.81E-03
DPYSL3	1.05	0.25	5.25E-04	44.88	1.18E-06
TAGLN3	0.79	0.19	5.25E-04	28.94	4.85E-04
HSPB1	-0.76	0.18	5.26E-04	26.28	1.26E-03
LTBP3	-0.76	0.18	5.28E-04	25.49	1.66E-03
MDH2	0.76	0.18	5.32E-04	26.19	1.30E-03
VPS29	0.76	0.18	5.32E-04	26.12	1.33E-03
SUV420H2	-0.76	0.18	5.32E-04	24.65	2.23E-03
PRODH	-0.76	0.18	5.35E-04	26.00	1.38E-03
MYO5B	0.76	0.18	5.37E-04	26.45	1.19E-03
LRRC43	0.91	0.22	5.39E-04	36.32	3.16E-05
C2orf40	0.76	0.18	5.39E-04	26.82	1.04E-03
FXYD1	-0.76	0.18	5.43E-04	25.79	1.49E-03
ANKMY1	0.76	0.18	5.47E-04	25.70	1.54E-03
LRRC27	0.76	0.18	5.50E-04	25.40	1.71E-03
PHF13	-0.76	0.18	5.51E-04	25.53	1.63E-03
CNTN2	-0.76	0.18	5.57E-04	25.84	1.47E-03
TRMT1	-0.76	0.18	5.57E-04	24.89	2.06E-03
WDR62	-0.76	0.18	5.67E-04	24.53	2.33E-03
DGCR14	-0.76	0.18	5.67E-04	24.71	2.19E-03
GMEB2	-0.76	0.18	5.67E-04	24.56	2.30E-03
PRKAB2	-0.76	0.18	5.68E-04	25.46	1.67E-03
RUNDC1	0.76	0.18	5.69E-04	24.77	2.14E-03
CTBS	1.22	0.29	5.69E-04	58.35	6.17E-09
RELT	-1.08	0.26	5.77E-04	45.70	8.66E-07
IL17D	-0.76	0.18	5.77E-04	25.53	1.63E-03

CD109	0.76	0.18	5.78E-04	26.29	1.26E-03
KLF2	0.76	0.18	5.81E-04	26.03	1.37E-03
ARL2BP	1.42	0.34	5.87E-04	71.59	2.85E-11
SORL1	0.76	0.18	5.90E-04	25.95	1.41E-03
TXLNB	1.28	0.31	5.90E-04	62.98	9.23E-10
GRIN2C	-0.87	0.21	5.93E-04	33.77	8.29E-05
CASP6	-0.76	0.18	5.94E-04	25.52	1.64E-03
RAI2	0.76	0.18	5.95E-04	25.88	1.44E-03
CRB1	-0.76	0.18	6.03E-04	25.89	1.44E-03
RABEPK	0.91	0.22	6.03E-04	34.62	6.05E-05
DRAM1	0.81	0.20	6.08E-04	29.33	4.23E-04
PELI2	0.76	0.18	6.08E-04	25.61	1.59E-03
SLC38A5	-0.92	0.22	6.08E-04	37.05	2.41E-05
CAPS	0.75	0.18	6.13E-04	25.40	1.71E-03
TCN1	0.75	0.18	6.20E-04	24.17	2.63E-03
RIOK2	0.93	0.23	6.20E-04	36.74	2.71E-05
SMTN	-0.75	0.18	6.23E-04	25.21	1.83E-03
SUPT7L	0.75	0.18	6.24E-04	25.72	1.53E-03
UCN3	-0.75	0.18	6.26E-04	24.65	2.23E-03
CCDC96	0.76	0.18	6.30E-04	26.80	1.05E-03
CHEK1	-0.78	0.19	6.30E-04	28.52	5.64E-04
TAF7	0.75	0.18	6.31E-04	26.08	1.35E-03
CCDC142	-0.93	0.23	6.31E-04	35.75	3.92E-05
TNFRSF19	0.80	0.19	6.34E-04	29.10	4.58E-04
TCAP	-0.75	0.18	6.34E-04	25.60	1.59E-03
WIF1	-0.77	0.19	6.34E-04	28.18	6.40E-04
ZNF592	0.83	0.20	6.35E-04	30.44	2.81E-04
POLA1	0.75	0.18	6.36E-04	25.31	1.77E-03
VASN	-0.75	0.18	6.37E-04	25.17	1.86E-03
NXPH3	-0.75	0.18	6.39E-04	24.84	2.09E-03
SEMA6A	-0.75	0.18	6.41E-04	25.66	1.56E-03
SEMA6B	-0.75	0.18	6.49E-04	25.97	1.40E-03
SMOX	-0.75	0.18	6.50E-04	24.87	2.07E-03
B4GALNT4	-0.92	0.22	6.51E-04	36.53	2.93E-05
NR1H3	-0.75	0.18	6.51E-04	25.73	1.52E-03
NARG2	0.81	0.20	6.51E-04	29.65	3.76E-04
IFITM1	-0.75	0.18	6.56E-04	25.01	1.97E-03
G6PC3	0.75	0.18	6.57E-04	25.26	1.80E-03
NAA20	0.75	0.18	6.57E-04	25.92	1.42E-03
APLNR	-0.75	0.18	6.62E-04	25.47	1.67E-03
ACP2	-0.75	0.18	6.64E-04	25.27	1.79E-03
ZDHHC1	0.96	0.23	6.64E-04	38.91	1.20E-05
YIPF4	0.75	0.18	6.65E-04	24.58	2.29E-03
SNCG	-0.75	0.18	6.66E-04	24.63	2.25E-03
VWA5A	0.75	0.18	6.82E-04	24.87	2.07E-03
ZNF706	0.75	0.18	6.90E-04	26.07	1.35E-03
PTGR1	0.75	0.18	6.94E-04	26.26	1.27E-03
CHTF8	0.79	0.19	7.01E-04	27.87	7.17E-04
DLEU2	-0.75	0.18	7.01E-04	24.42	2.42E-03
PDGFRB	-1.14	0.28	7.01E-04	52.97	4.93E-08
NES	-0.75	0.18	7.02E-04	24.93	2.03E-03
ANGPTL7	0.75	0.18	7.02E-04	25.40	1.71E-03
PPFIA3	-0.95	0.23	7.03E-04	36.81	2.64E-05
AKAP13	0.75	0.18	7.09E-04	25.77	1.50E-03
PBK	-0.75	0.18	7.13E-04	25.07	1.93E-03
ZMAT2	0.77	0.19	7.14E-04	27.27	8.88E-04
CACNG7	-0.75	0.18	7.14E-04	24.31	2.52E-03
ZBED4	-0.90	0.22	7.14E-04	34.38	6.60E-05

NFIB	0.74	0.18	7.29E-04	24.84	2.09E-03
BCAN	-0.74	0.18	7.31E-04	25.19	1.85E-03
CMTM3	0.75	0.18	7.32E-04	25.63	1.58E-03
C12orf66	0.74	0.18	7.35E-04	24.82	2.11E-03
SPAG9	0.93	0.23	7.35E-04	37.14	2.34E-05
LRRC46	0.81	0.20	7.38E-04	29.95	3.35E-04
LMF2	-0.74	0.18	7.41E-04	24.16	2.64E-03
ALCAM	0.81	0.20	7.42E-04	30.17	3.09E-04
OPRL1	-0.74	0.18	7.42E-04	23.72	3.08E-03
SLC25A23	-0.74	0.18	7.42E-04	24.77	2.14E-03
CCDC104	1.15	0.28	7.42E-04	53.09	4.72E-08
RFTN1	1.16	0.29	7.43E-04	52.96	4.93E-08
ARSD	0.74	0.18	7.46E-04	25.86	1.45E-03
SLC4A7	-0.78	0.19	7.47E-04	28.30	6.11E-04
SOX6	0.74	0.18	7.53E-04	24.90	2.05E-03
AJAP1	-0.74	0.18	7.54E-04	24.91	2.05E-03
RPE65	0.88	0.22	7.56E-04	34.71	5.85E-05
PKN3	-0.75	0.19	7.56E-04	25.72	1.53E-03
CBX8	-0.74	0.18	7.57E-04	24.55	2.31E-03
ANTXR2	0.81	0.20	7.67E-04	30.16	3.10E-04
LIPE	-0.74	0.18	7.74E-04	26.22	1.28E-03
PLEKHM2	-0.93	0.23	7.75E-04	36.58	2.88E-05
PRDX4	0.74	0.18	7.77E-04	24.41	2.43E-03
ZNF800	1.04	0.26	7.84E-04	44.46	1.38E-06
DMD	1.03	0.25	7.86E-04	45.21	1.04E-06
ZNF324	-0.74	0.18	7.86E-04	24.10	2.70E-03
AKNA	0.74	0.18	7.93E-04	24.98	1.99E-03
TRMT61B	0.80	0.20	7.93E-04	28.95	4.84E-04
ZNHIT1	0.74	0.18	7.93E-04	23.48	3.33E-03
CDHR1	-0.74	0.18	7.93E-04	26.40	1.21E-03
ARMCX1	1.06	0.26	7.96E-04	47.02	5.26E-07
MAP4K2	-0.74	0.18	7.98E-04	24.72	2.17E-03
CRELD1	-0.74	0.18	7.99E-04	23.61	3.19E-03
GFOD1	-0.98	0.24	8.02E-04	39.45	9.69E-06
MDM2	0.74	0.18	8.05E-04	25.20	1.84E-03
DOCK6	-0.74	0.18	8.08E-04	25.25	1.81E-03
KBTBD12	-0.74	0.18	8.09E-04	24.79	2.13E-03
LSR	-0.74	0.18	8.11E-04	25.96	1.40E-03
SIGMAR1	0.74	0.18	8.14E-04	25.14	1.88E-03
CACNA1I	-0.74	0.18	8.14E-04	25.24	1.82E-03
KCMF1	0.74	0.18	8.18E-04	23.96	2.83E-03
RRM2B	0.74	0.18	8.20E-04	24.48	2.37E-03
VEZF1	0.90	0.22	8.22E-04	35.38	4.54E-05
GFM2	0.87	0.22	8.24E-04	33.87	7.99E-05
PDE5A	-0.88	0.22	8.24E-04	34.80	5.67E-05
C5orf22	0.87	0.21	8.26E-04	32.92	1.14E-04
UNG	0.93	0.23	8.31E-04	37.42	2.13E-05
RTN2	-0.74	0.18	8.42E-04	24.12	2.68E-03
TSEN54	-0.74	0.18	8.52E-04	24.64	2.24E-03
ELAC2	0.74	0.18	8.53E-04	23.56	3.24E-03
MTA1	-1.27	0.31	8.55E-04	57.15	9.59E-09
IFT172	0.74	0.18	8.56E-04	24.46	2.38E-03
KRT5	-0.74	0.18	8.56E-04	24.27	2.55E-03
ZNF589	-0.74	0.18	8.56E-04	26.15	1.32E-03
ZNF503	0.74	0.18	8.64E-04	24.19	2.62E-03
HCN1	-0.74	0.18	8.66E-04	24.26	2.56E-03
ARMC3	0.74	0.18	8.69E-04	25.48	1.66E-03
CCNB2	-0.73	0.18	8.75E-04	24.42	2.42E-03

NF2	-1.05	0.26	8.75E-04	42.92	2.53E-06
GABRQ	-0.73	0.18	8.76E-04	24.23	2.58E-03
TROAP	-0.73	0.18	8.83E-04	24.24	2.58E-03
ZNF573	-0.73	0.18	8.90E-04	24.23	2.59E-03
SEMA5B	-0.73	0.18	9.00E-04	24.23	2.58E-03
PIGQ	0.73	0.18	9.00E-04	23.85	2.95E-03
CRABP2	-0.73	0.18	9.01E-04	24.18	2.63E-03
RNF185	-0.73	0.18	9.02E-04	24.13	2.66E-03
DYNLT3	0.73	0.18	9.03E-04	24.44	2.40E-03
SNRNP27	0.73	0.18	9.03E-04	23.75	3.04E-03
PREX1	-1.19	0.30	9.09E-04	55.51	1.87E-08
FAS	0.73	0.18	9.10E-04	25.14	1.88E-03
ACAD9	-0.73	0.18	9.15E-04	24.18	2.63E-03
GRIA4	-0.73	0.18	9.16E-04	25.06	1.93E-03
PDZRN3	-0.73	0.18	9.16E-04	24.57	2.30E-03
MDFI	-0.73	0.18	9.17E-04	24.49	2.36E-03
CLCF1	0.73	0.18	9.19E-04	24.83	2.10E-03
RGS20	-0.73	0.18	9.24E-04	25.71	1.53E-03
LEPRE1	-0.73	0.18	9.26E-04	25.01	1.97E-03
LHFPL1	0.73	0.18	9.29E-04	24.90	2.05E-03
MPP5	0.79	0.20	9.38E-04	28.62	5.46E-04
ADORA2A	-0.73	0.18	9.42E-04	24.40	2.43E-03
SEPT4	-0.73	0.18	9.47E-04	25.60	1.59E-03
FARS2	0.90	0.22	9.54E-04	34.75	5.76E-05
HHLA3	0.73	0.18	9.56E-04	23.75	3.04E-03
OCEL1	0.73	0.18	9.56E-04	23.89	2.90E-03
ABLIM1	0.73	0.18	9.58E-04	23.56	3.25E-03
ARID3B	-0.73	0.18	9.63E-04	24.07	2.72E-03
DACT3	-0.73	0.18	9.73E-04	25.24	1.81E-03
RANBP2	0.73	0.18	9.74E-04	23.51	3.30E-03
SPRY2	-0.73	0.18	9.76E-04	24.76	2.14E-03
FBXL19	-0.73	0.18	9.81E-04	23.07	3.83E-03
TMEM54	1.24	0.31	9.82E-04	60.74	2.37E-09
OBSCN	-0.73	0.18	9.86E-04	24.61	2.26E-03
CORO6	-0.73	0.18	9.89E-04	25.09	1.91E-03
DIO3	-0.73	0.18	9.89E-04	25.99	1.39E-03
RPS19BP1	-0.91	0.23	9.89E-04	34.52	6.26E-05
HAPLN4	-0.73	0.18	9.91E-04	25.16	1.87E-03
FEZ1	-0.73	0.18	9.95E-04	23.83	2.96E-03
CDK6	0.73	0.18	9.97E-04	24.22	2.59E-03
DHTKD1	-0.73	0.18	1.00E-03	24.11	2.68E-03
DNASE2	0.73	0.18	1.01E-03	23.64	3.16E-03
ASPM	-0.73	0.18	1.01E-03	24.16	2.64E-03
LZTS2	-0.73	0.18	1.02E-03	24.26	2.56E-03
HINFP	-0.97	0.24	1.02E-03	37.19	2.30E-05
HAGH	0.73	0.18	1.03E-03	23.62	3.18E-03
EIF1AX	0.73	0.18	1.03E-03	24.27	2.55E-03
SLX4	-0.73	0.18	1.03E-03	23.14	3.74E-03
VLDLR	0.73	0.18	1.04E-03	24.24	2.58E-03
RGR	-0.73	0.18	1.04E-03	23.49	3.32E-03
PWP1	0.73	0.18	1.04E-03	24.63	2.25E-03
NPHP1	0.93	0.23	1.04E-03	37.91	1.78E-05
PLD2	-0.72	0.18	1.04E-03	23.33	3.50E-03
GALE	-0.73	0.18	1.05E-03	23.83	2.96E-03
TGM5	0.73	0.18	1.05E-03	24.48	2.37E-03
SEL1L3	-0.72	0.18	1.06E-03	24.31	2.51E-03
CDCA5	-0.72	0.18	1.06E-03	24.39	2.44E-03
MGAT5B	-0.72	0.18	1.06E-03	24.30	2.52E-03

BAHCC1	-0.72	0.18	1.06E-03	24.38	2.45E-03
ZDHHC8	-0.72	0.18	1.07E-03	24.82	2.11E-03
PLBD1	0.72	0.18	1.07E-03	23.57	3.23E-03
RNF24	-1.19	0.30	1.08E-03	56.19	1.43E-08
COX18	0.72	0.18	1.08E-03	23.45	3.36E-03
LRRC23	0.88	0.22	1.08E-03	34.70	5.87E-05
TMF1	0.72	0.18	1.08E-03	24.25	2.57E-03
RIMS4	-0.72	0.18	1.09E-03	24.32	2.51E-03
RHBDD3	-0.72	0.18	1.09E-03	23.40	3.42E-03
SNRNP25	0.72	0.18	1.09E-03	23.57	3.23E-03
POLR2F	-0.72	0.18	1.09E-03	24.15	2.65E-03
PNMT	-0.72	0.18	1.10E-03	24.24	2.57E-03
COL4A2	-0.84	0.21	1.10E-03	32.51	1.33E-04
DPM2	0.93	0.23	1.11E-03	35.81	3.83E-05
WDYHV1	-0.72	0.18	1.11E-03	23.66	3.14E-03
CCDC113	1.05	0.27	1.11E-03	46.88	5.49E-07
CDS1	1.34	0.34	1.11E-03	69.08	7.50E-11
MDM1	1.25	0.32	1.12E-03	61.86	1.43E-09
MGAT3	-0.75	0.19	1.12E-03	26.57	1.14E-03
CACNA1G	-0.72	0.18	1.13E-03	23.58	3.23E-03
TTC31	-0.80	0.20	1.13E-03	28.38	5.94E-04
ATG5	0.72	0.18	1.14E-03	24.80	2.12E-03
UBE3B	0.72	0.18	1.15E-03	22.36	4.89E-03
PTPRM	0.72	0.18	1.15E-03	23.12	3.77E-03
CDC42BPB	-0.72	0.18	1.15E-03	23.14	3.74E-03
CIC	-1.11	0.28	1.15E-03	47.27	4.73E-07
RNPC3	-0.72	0.18	1.16E-03	23.29	3.55E-03
KLHDC8B	-0.73	0.19	1.17E-03	25.50	1.65E-03
VEGFA	-0.72	0.18	1.17E-03	24.58	2.29E-03
PGM5	1.05	0.27	1.17E-03	46.63	6.05E-07
C3orf18	0.72	0.18	1.18E-03	22.34	4.92E-03
MAPKAPK3	-0.72	0.18	1.18E-03	23.39	3.44E-03
TRAPPC2	0.72	0.18	1.18E-03	24.17	2.64E-03
SYNPO	1.32	0.34	1.18E-03	63.36	7.90E-10
AARS2	-0.72	0.18	1.18E-03	23.84	2.95E-03
LRTOMT	0.91	0.23	1.19E-03	36.62	2.84E-05
PSMD9	0.72	0.18	1.20E-03	23.25	3.61E-03
INTS10	0.72	0.18	1.20E-03	24.05	2.74E-03
CDC42EP4	-0.86	0.22	1.20E-03	33.19	1.03E-04
NDUFA5	0.94	0.24	1.21E-03	38.68	1.31E-05
TACC3	-0.72	0.18	1.22E-03	23.48	3.33E-03
SLC6A16	1.02	0.26	1.22E-03	43.77	1.82E-06
BBS5	1.01	0.26	1.22E-03	43.50	2.03E-06
HOXD9	0.72	0.18	1.22E-03	23.52	3.30E-03
CCDC87	0.72	0.18	1.22E-03	23.30	3.54E-03
FTO	0.72	0.18	1.22E-03	23.79	2.99E-03
HSD17B4	0.72	0.18	1.22E-03	23.54	3.27E-03
AMZ2	0.72	0.18	1.22E-03	24.12	2.68E-03
NGRN	0.72	0.18	1.23E-03	23.84	2.95E-03
RIC8B	0.81	0.21	1.23E-03	30.25	3.01E-04
GPR125	-1.09	0.28	1.23E-03	47.68	4.04E-07
SLCO4A1	-0.72	0.18	1.23E-03	23.77	3.02E-03
TMEM66	0.76	0.19	1.23E-03	27.01	9.74E-04
PLOD3	0.75	0.19	1.23E-03	25.82	1.48E-03
DNAH5	0.78	0.20	1.23E-03	28.57	5.55E-04
C6orf118	0.72	0.18	1.24E-03	23.73	3.06E-03
CYP2U1	0.73	0.19	1.24E-03	25.20	1.84E-03
RBL2	0.72	0.18	1.24E-03	23.04	3.87E-03

FSD1	-0.71	0.18	1.24E-03	23.02	3.90E-03
ADK	0.72	0.18	1.24E-03	24.00	2.79E-03
BOK	-0.71	0.18	1.24E-03	23.57	3.24E-03
EIF4G3	0.71	0.18	1.25E-03	23.11	3.78E-03
PICK1	-0.85	0.22	1.25E-03	31.33	2.03E-04
RNF20	1.14	0.29	1.25E-03	52.33	6.31E-08
ZNF75D	0.96	0.25	1.26E-03	39.79	8.44E-06
B3GNT2	0.87	0.22	1.27E-03	34.21	7.00E-05
HNF1B	1.21	0.31	1.27E-03	56.26	1.40E-08
GXYLT2	0.71	0.18	1.28E-03	23.08	3.82E-03
OCRL	1.26	0.32	1.28E-03	62.19	1.26E-09
BEND6	0.71	0.18	1.28E-03	24.78	2.14E-03
SLC43A2	-0.71	0.18	1.28E-03	23.23	3.63E-03
ESYT1	0.71	0.18	1.29E-03	24.26	2.56E-03
TRAF3IP2	0.71	0.18	1.30E-03	23.82	2.97E-03
CRTAC1	-0.71	0.18	1.30E-03	23.45	3.36E-03
CYB561	0.71	0.18	1.30E-03	23.03	3.88E-03
CSMD1	-0.71	0.18	1.30E-03	23.98	2.81E-03
PPP2R5C	-0.71	0.18	1.30E-03	23.92	2.88E-03
RAB26	-0.71	0.18	1.30E-03	24.63	2.25E-03
VSTM4	0.82	0.21	1.30E-03	30.97	2.32E-04
WT1-AS	1.39	0.36	1.30E-03	68.48	9.61E-11
TRPC4	0.71	0.18	1.30E-03	23.83	2.96E-03
SLCO1C1	-0.71	0.18	1.30E-03	23.02	3.90E-03
ALS2	0.71	0.18	1.31E-03	24.42	2.42E-03
LEAP2	0.71	0.18	1.31E-03	23.12	3.77E-03
MORC2-AS1	-0.71	0.18	1.31E-03	23.50	3.31E-03
PATZ1	-0.71	0.18	1.31E-03	22.54	4.60E-03
PTOV1	-0.71	0.18	1.31E-03	22.44	4.75E-03
ADCY4	-0.71	0.18	1.31E-03	23.25	3.61E-03
BCL2	0.71	0.18	1.33E-03	23.86	2.93E-03
CLCN4	0.71	0.18	1.33E-03	23.00	3.93E-03
PNPLA2	-0.75	0.19	1.33E-03	26.57	1.14E-03
COG7	0.71	0.18	1.34E-03	22.83	4.16E-03
DNAJC3	0.71	0.18	1.34E-03	22.84	4.14E-03
TNS3	0.71	0.18	1.34E-03	23.52	3.29E-03
ANKLE1	-0.71	0.18	1.34E-03	23.21	3.65E-03
SLC9A3R1	-0.71	0.18	1.34E-03	23.72	3.08E-03
RANGRF	0.77	0.20	1.34E-03	26.86	1.02E-03
MRPS17	0.90	0.23	1.35E-03	35.15	4.94E-05
IFFO1	-0.71	0.18	1.35E-03	22.78	4.23E-03
HOXB2	0.71	0.18	1.36E-03	24.14	2.66E-03
MLLT6	0.86	0.22	1.37E-03	31.38	1.99E-04
ASPA	-0.71	0.18	1.37E-03	25.34	1.75E-03
CUBN	0.71	0.18	1.37E-03	23.97	2.83E-03
SOX30	1.05	0.27	1.37E-03	45.32	9.94E-07
NEXN	0.71	0.18	1.37E-03	23.03	3.89E-03
C19orf25	-0.71	0.18	1.38E-03	23.91	2.88E-03
CSRP1	-0.71	0.18	1.38E-03	22.92	4.03E-03
FKBP10	-1.07	0.28	1.38E-03	47.40	4.50E-07
C2CD2	-0.71	0.18	1.38E-03	22.82	4.17E-03
SMARCA4	-0.71	0.18	1.39E-03	23.86	2.93E-03
TBCA	0.71	0.18	1.39E-03	22.39	4.84E-03
C16orf80	1.12	0.29	1.39E-03	51.90	7.42E-08
WDR27	0.71	0.18	1.40E-03	22.48	4.68E-03
BCAP31	0.71	0.18	1.40E-03	23.18	3.69E-03
EPAS1	-0.71	0.18	1.40E-03	22.89	4.08E-03
MCTP1	0.71	0.18	1.41E-03	23.08	3.83E-03

MIF4GD	0.85	0.22	1.42E-03	32.41	1.37E-04
SLN	1.16	0.30	1.43E-03	54.66	2.56E-08
C7orf10	0.71	0.18	1.43E-03	23.43	3.38E-03
ENHO	-0.71	0.18	1.44E-03	23.34	3.49E-03
S100A11	0.71	0.18	1.44E-03	24.49	2.36E-03
LAS1L	0.79	0.20	1.45E-03	27.91	7.08E-04
FAHD2A	0.71	0.18	1.46E-03	23.13	3.76E-03
NCS1	0.71	0.18	1.46E-03	24.09	2.71E-03
MAP4K4	-0.80	0.21	1.46E-03	29.31	4.25E-04
ANKFN1	0.86	0.22	1.46E-03	33.74	8.36E-05
ATXN7L3B	0.71	0.18	1.47E-03	24.17	2.63E-03
PLD5	1.33	0.35	1.47E-03	68.52	9.52E-11
AIF1L	-0.70	0.18	1.47E-03	23.04	3.87E-03
S100A14	-0.70	0.18	1.47E-03	22.50	4.64E-03
COG1	0.71	0.18	1.47E-03	22.93	4.03E-03
RBMS1	0.71	0.18	1.48E-03	24.00	2.80E-03
TRPM4	-0.86	0.22	1.48E-03	32.16	1.51E-04
WDR13	0.70	0.18	1.49E-03	22.85	4.13E-03
HERPUD2	0.70	0.18	1.49E-03	22.74	4.29E-03
TRIM4	0.70	0.18	1.50E-03	23.78	3.01E-03
NFKBIE	-0.70	0.18	1.51E-03	22.52	4.63E-03
ZCCHC9	0.70	0.18	1.51E-03	21.94	5.63E-03
FUNDC2	1.18	0.31	1.51E-03	53.02	4.84E-08
RAB11FIP4	-0.70	0.18	1.52E-03	24.26	2.56E-03
PNO1	0.76	0.20	1.52E-03	26.84	1.03E-03
P4HA2	0.70	0.18	1.52E-03	23.37	3.46E-03
SHMT2	-0.84	0.22	1.52E-03	32.59	1.29E-04
APH1B	0.99	0.26	1.52E-03	42.41	3.09E-06
IPO8	0.70	0.18	1.53E-03	21.37	6.81E-03
USP39	0.70	0.18	1.53E-03	22.09	5.36E-03
DISP2	-0.70	0.18	1.54E-03	23.07	3.83E-03
C15orf40	0.84	0.22	1.55E-03	31.60	1.84E-04
HOXC6	2.29	0.60	1.55E-03	153.77	0.00E+00
KIF1A	-0.82	0.21	1.56E-03	30.84	2.44E-04
ERBB2	0.70	0.18	1.56E-03	22.71	4.32E-03
EGF	0.70	0.18	1.56E-03	22.85	4.12E-03
GAB1	-0.90	0.23	1.57E-03	36.35	3.12E-05
DNAH2	0.79	0.21	1.57E-03	29.26	4.34E-04
CDC45	-0.70	0.18	1.58E-03	23.15	3.74E-03
MID1IP1	0.84	0.22	1.58E-03	31.85	1.69E-04
PPP2R2A	0.70	0.18	1.59E-03	23.64	3.16E-03
SLC35B4	0.82	0.21	1.59E-03	30.59	2.67E-04
RIOK1	0.70	0.18	1.59E-03	22.47	4.70E-03
OPCML	-0.70	0.18	1.59E-03	23.29	3.55E-03
LINC00094	0.76	0.20	1.60E-03	27.15	9.26E-04
RGS13	0.70	0.18	1.60E-03	22.16	5.24E-03
ENDOU	-0.70	0.18	1.61E-03	22.64	4.43E-03
GDPD2	0.70	0.18	1.62E-03	23.87	2.93E-03
CAPN10	-1.31	0.34	1.62E-03	59.74	3.52E-09
IKZF5	0.70	0.18	1.63E-03	23.10	3.80E-03
SPATS2	-0.70	0.18	1.63E-03	23.39	3.43E-03
RFXANK	-0.70	0.18	1.63E-03	21.95	5.62E-03
KIF6	1.14	0.30	1.63E-03	53.74	3.64E-08
SUSD1	-0.70	0.18	1.64E-03	22.92	4.04E-03
HPS4	-0.85	0.22	1.65E-03	31.68	1.79E-04
ZDHHC13	0.77	0.20	1.68E-03	27.87	7.15E-04
CHSY1	-0.70	0.18	1.68E-03	23.62	3.18E-03
KPTN	-0.89	0.23	1.69E-03	33.65	8.65E-05

LRRC6	0.88	0.23	1.69E-03	35.23	4.80E-05
FBXO6	0.70	0.18	1.69E-03	23.25	3.61E-03
CCDC126	1.01	0.27	1.69E-03	43.78	1.82E-06
FAM43A	-0.70	0.18	1.69E-03	22.47	4.70E-03
RPL28	-0.70	0.18	1.69E-03	21.83	5.83E-03
TFF3	0.77	0.20	1.69E-03	28.13	6.52E-04
ZDHHC14	1.01	0.26	1.70E-03	43.17	2.32E-06
PDCD11	-0.70	0.18	1.70E-03	21.89	5.71E-03
TAPBPL	0.70	0.18	1.70E-03	22.06	5.42E-03
MMEL1	0.70	0.18	1.71E-03	22.93	4.03E-03
LGR5	-0.70	0.18	1.71E-03	22.53	4.61E-03
STXBP1	0.70	0.18	1.73E-03	22.65	4.43E-03
MTG1	-0.70	0.18	1.74E-03	23.48	3.33E-03
TBC1D10A	-0.70	0.18	1.74E-03	22.00	5.53E-03
GAD1	-0.69	0.18	1.75E-03	23.02	3.89E-03
SUSD2	-0.69	0.18	1.75E-03	22.52	4.63E-03
NEFM	0.69	0.18	1.75E-03	23.10	3.80E-03
PSTK	0.69	0.18	1.75E-03	22.15	5.24E-03
AURKB	-0.69	0.18	1.78E-03	23.18	3.70E-03
SLC4A8	-0.69	0.18	1.78E-03	23.06	3.85E-03
PKP4	0.69	0.18	1.80E-03	22.87	4.11E-03
ATP6V1G1	1.14	0.30	1.81E-03	52.35	6.30E-08
DICER1	-0.69	0.18	1.81E-03	21.80	5.89E-03
MEN1	-0.77	0.20	1.82E-03	27.06	9.59E-04
LEPROTL1	0.69	0.18	1.82E-03	23.48	3.33E-03
ESAM	-0.69	0.18	1.82E-03	22.40	4.82E-03
GSDMC	0.69	0.18	1.83E-03	21.98	5.57E-03
EPM2A	1.03	0.27	1.83E-03	44.65	1.28E-06
SLC9A5	-0.76	0.20	1.83E-03	26.67	1.09E-03
DENND1B	0.69	0.18	1.84E-03	23.37	3.46E-03
MKRN3	-0.69	0.18	1.84E-03	21.75	6.00E-03
OPLAH	-1.21	0.32	1.84E-03	54.66	2.56E-08
RASSF3	-0.69	0.18	1.85E-03	22.92	4.04E-03
PROM1	0.69	0.18	1.86E-03	23.53	3.28E-03
ZNF165	1.02	0.27	1.86E-03	43.49	2.04E-06
CDK20	0.80	0.21	1.87E-03	29.49	3.99E-04
FAM86C1	0.75	0.20	1.87E-03	26.20	1.29E-03
LRP3	-0.69	0.18	1.88E-03	22.46	4.71E-03
IL17RB	-1.05	0.28	1.88E-03	44.66	1.28E-06
PTPN21	0.79	0.21	1.89E-03	29.41	4.11E-04
RIMKLB	0.69	0.18	1.89E-03	21.69	6.13E-03
ZNF394	0.82	0.22	1.89E-03	29.08	4.61E-04
KIRREL2	-0.70	0.19	1.89E-03	23.95	2.84E-03
MT3	-0.70	0.19	1.89E-03	24.45	2.39E-03
TSPYL4	0.69	0.18	1.91E-03	22.86	4.12E-03
MAGI3	-0.72	0.19	1.91E-03	25.07	1.93E-03
AR	0.69	0.18	1.92E-03	22.15	5.24E-03
CCDC148	1.20	0.32	1.92E-03	57.76	7.88E-09
THNSL2	0.69	0.18	1.94E-03	23.15	3.74E-03
CAMK2D	-0.99	0.26	1.94E-03	42.21	3.33E-06
UFC1	0.69	0.18	1.95E-03	22.87	4.11E-03
GSN	-0.69	0.18	1.95E-03	23.43	3.39E-03
PARD6B	0.92	0.24	1.95E-03	37.43	2.12E-05
CDCA3	-0.69	0.18	1.96E-03	21.67	6.17E-03
ZNRF1	-0.69	0.18	1.96E-03	22.40	4.83E-03
KIF7	-0.69	0.18	1.96E-03	21.81	5.87E-03
CLYBL	-0.69	0.18	1.96E-03	23.16	3.72E-03
RASSF1	-0.69	0.18	1.96E-03	21.71	6.08E-03

TUBGCP6	-1.08	0.29	1.96E-03	46.65	6.01E-07
PFKFB4	-0.69	0.18	1.97E-03	21.75	6.01E-03
MRPS2	0.88	0.23	1.97E-03	34.29	6.81E-05
FOXA2	0.93	0.25	1.98E-03	37.50	2.07E-05
AQP1	0.74	0.20	1.99E-03	26.35	1.23E-03
GPR133	-0.69	0.18	1.99E-03	22.73	4.29E-03
EML3	-0.90	0.24	1.99E-03	36.02	3.53E-05
RTTN	-0.69	0.18	2.01E-03	21.95	5.62E-03
MID2	1.28	0.34	2.01E-03	64.87	4.16E-10
KLF15	-0.69	0.18	2.01E-03	22.59	4.51E-03
NMB	1.24	0.33	2.01E-03	58.97	4.74E-09
MOSPD1	0.97	0.26	2.01E-03	41.02	5.26E-06
LRRN2	-0.97	0.26	2.02E-03	39.59	9.16E-06
TAF1C	0.79	0.21	2.02E-03	28.41	5.87E-04
SYT1	-0.69	0.18	2.03E-03	21.71	6.08E-03
PPP2R3C	-0.69	0.18	2.04E-03	22.37	4.87E-03
SCARA3	-0.85	0.23	2.05E-03	33.04	1.09E-04
MUM1	-0.69	0.18	2.05E-03	22.29	5.01E-03
ZNF526	-0.70	0.19	2.06E-03	22.65	4.43E-03
CCNH	1.05	0.28	2.07E-03	43.89	1.73E-06
PLCB1	-0.80	0.21	2.07E-03	30.10	3.17E-04
TP53I13	-0.79	0.21	2.08E-03	28.67	5.36E-04
SUMF1	0.68	0.18	2.12E-03	21.58	6.35E-03
APOOL	0.77	0.20	2.13E-03	27.58	7.98E-04
FAM84B	0.68	0.18	2.13E-03	21.90	5.69E-03
PNMA2	1.04	0.28	2.13E-03	46.09	7.46E-07
PPAP2C	-0.68	0.18	2.13E-03	22.10	5.34E-03
BAP1	-0.68	0.18	2.13E-03	21.93	5.65E-03
PLEKHA3	1.03	0.28	2.14E-03	43.92	1.72E-06
ITIH5	-0.68	0.18	2.15E-03	22.61	4.49E-03
CILP	1.97	0.53	2.15E-03	114.52	0.00E+00
PLEKHG2	-0.92	0.25	2.15E-03	37.73	1.90E-05
XXYLT1	-0.68	0.18	2.16E-03	22.51	4.63E-03
FKTN	0.68	0.18	2.16E-03	22.71	4.33E-03
OXSR1	-0.68	0.18	2.17E-03	21.73	6.04E-03
WDR20	-0.68	0.18	2.17E-03	21.89	5.72E-03
DHX34	-0.83	0.22	2.18E-03	30.64	2.62E-04
STX8	0.68	0.18	2.19E-03	21.28	7.01E-03
CEP170	-0.68	0.18	2.20E-03	22.28	5.03E-03
SLC24A1	0.68	0.18	2.20E-03	21.03	7.66E-03
SRSF10	-0.77	0.21	2.20E-03	26.70	1.08E-03
PDE2A	-0.68	0.18	2.20E-03	21.73	6.04E-03
PPP1R21	1.04	0.28	2.20E-03	45.18	1.05E-06
KIF26A	-0.68	0.18	2.21E-03	21.60	6.29E-03
ZDHHC23	1.08	0.29	2.21E-03	47.75	3.94E-07
PVRL1	-0.68	0.18	2.21E-03	21.27	7.04E-03
ABLIM2	0.73	0.20	2.21E-03	25.91	1.43E-03
RASAL1	-0.68	0.18	2.22E-03	21.08	7.53E-03
CMBL	0.68	0.18	2.23E-03	22.76	4.26E-03
CTDSPL	-0.68	0.18	2.24E-03	21.23	7.14E-03
PLEKHJ1	-0.68	0.18	2.24E-03	21.61	6.28E-03
PLIN3	-0.68	0.18	2.24E-03	21.23	7.14E-03
FRMD5	0.71	0.19	2.25E-03	24.85	2.08E-03
SFRP2	-0.68	0.18	2.26E-03	21.95	5.62E-03
TOM1	-0.68	0.18	2.26E-03	21.87	5.75E-03
BBS10	0.80	0.22	2.26E-03	30.10	3.17E-04
NPAS2	0.73	0.20	2.27E-03	26.05	1.36E-03
MAST1	-0.68	0.18	2.30E-03	21.44	6.66E-03

BAIAP2	-0.68	0.18	2.30E-03	23.03	3.89E-03
SH3BP2	-0.68	0.18	2.31E-03	21.62	6.27E-03
VPS4B	0.68	0.18	2.31E-03	21.53	6.43E-03
MUS81	-0.84	0.23	2.31E-03	29.23	4.38E-04
SLC7A11	0.69	0.19	2.32E-03	23.65	3.15E-03
SLC4A1AP	0.68	0.18	2.33E-03	20.54	9.03E-03
WFDC1	0.87	0.24	2.33E-03	34.17	7.12E-05
CALHM2	-0.68	0.18	2.34E-03	21.33	6.90E-03
SRBD1	0.68	0.18	2.35E-03	20.93	7.92E-03
KIF23	-0.68	0.18	2.35E-03	22.21	5.15E-03
RAB30	-0.68	0.18	2.35E-03	21.94	5.63E-03
CDK9	0.87	0.23	2.35E-03	33.52	9.06E-05
RAB10	0.68	0.18	2.36E-03	21.76	5.98E-03
GPR124	-0.68	0.18	2.37E-03	21.61	6.28E-03
CDK7	0.89	0.24	2.37E-03	34.83	5.59E-05
PAQR3	0.68	0.18	2.38E-03	22.61	4.49E-03
NEK11	0.68	0.18	2.38E-03	22.65	4.43E-03
DYNC1LI2	0.89	0.24	2.40E-03	35.59	4.17E-05
SPRYD3	0.68	0.18	2.40E-03	22.14	5.27E-03
ABCA8	-1.01	0.27	2.40E-03	41.91	3.74E-06
CMAS	0.68	0.18	2.41E-03	22.41	4.79E-03
STOML3	0.93	0.25	2.41E-03	38.97	1.17E-05
GULP1	0.72	0.19	2.41E-03	25.27	1.79E-03
B9D1	0.75	0.20	2.43E-03	26.85	1.03E-03
SLC25A41	-0.68	0.18	2.43E-03	21.66	6.19E-03
FIGN	-0.68	0.18	2.43E-03	22.18	5.19E-03
DMKN	1.07	0.29	2.46E-03	48.44	2.97E-07
BAI1	-0.68	0.18	2.46E-03	22.03	5.48E-03
LZTR1	-1.08	0.29	2.46E-03	48.31	3.12E-07
ASNSD1	0.68	0.18	2.47E-03	22.20	5.17E-03
AIFM1	0.68	0.18	2.47E-03	22.59	4.52E-03
NDNL2	0.87	0.24	2.50E-03	33.93	7.80E-05
VOPP1	0.81	0.22	2.51E-03	30.58	2.67E-04
GSTK1	0.67	0.18	2.51E-03	21.58	6.33E-03
CYSLTR1	0.69	0.19	2.51E-03	23.22	3.65E-03
ABLIM3	-0.67	0.18	2.52E-03	21.99	5.55E-03
EDEM2	0.67	0.18	2.53E-03	22.39	4.83E-03
CDKN3	-0.67	0.18	2.53E-03	21.38	6.80E-03
ENO3	-0.67	0.18	2.53E-03	20.84	8.16E-03
C5orf15	0.67	0.18	2.53E-03	22.17	5.22E-03
IFIT1	0.67	0.18	2.53E-03	21.53	6.43E-03
B9D2	0.67	0.18	2.54E-03	22.25	5.09E-03
RANGAP1	-0.67	0.18	2.54E-03	21.51	6.48E-03
RAB18	0.67	0.18	2.54E-03	21.93	5.64E-03
LGR6	0.93	0.25	2.57E-03	38.27	1.54E-05
TOMM5	0.67	0.18	2.57E-03	22.52	4.62E-03
CDS2	0.83	0.23	2.59E-03	31.05	2.26E-04
POLR2I	0.71	0.19	2.59E-03	24.61	2.27E-03
RERG	0.80	0.22	2.59E-03	30.06	3.21E-04
ADCK2	0.82	0.22	2.61E-03	29.99	3.30E-04
FAM199X	0.67	0.18	2.61E-03	21.40	6.75E-03
MARK4	-0.67	0.18	2.61E-03	20.16	1.02E-02
MORC2	-0.67	0.18	2.61E-03	21.66	6.19E-03
KLF10	0.67	0.18	2.61E-03	21.50	6.52E-03
MOGS	-0.92	0.25	2.61E-03	36.28	3.20E-05
MIS18A	0.67	0.18	2.61E-03	21.92	5.66E-03
TRMT11	0.67	0.18	2.62E-03	20.74	8.44E-03
C10orf32	0.70	0.19	2.62E-03	23.85	2.95E-03

IFITM2	-0.67	0.18	2.62E-03	21.15	7.35E-03
VWF	-0.67	0.18	2.62E-03	22.26	5.07E-03
GLTSCR2	-0.67	0.18	2.62E-03	21.72	6.05E-03
CEP63	0.86	0.23	2.62E-03	33.56	8.93E-05
TATDN2	-0.67	0.18	2.64E-03	21.56	6.38E-03
CLIC2	0.67	0.18	2.66E-03	22.13	5.28E-03
TMEM62	0.67	0.18	2.66E-03	20.99	7.76E-03
USP7	0.67	0.18	2.67E-03	21.42	6.69E-03
RAB3B	1.15	0.31	2.67E-03	52.03	7.00E-08
NCOR1	0.67	0.18	2.67E-03	21.13	7.40E-03
NAAA	0.67	0.18	2.71E-03	22.17	5.22E-03
CMTM5	-0.67	0.18	2.71E-03	21.91	5.69E-03
ITGBL1	-0.67	0.18	2.71E-03	22.26	5.07E-03
LPHN3	-0.89	0.24	2.72E-03	35.98	3.58E-05
WSCD2	-0.67	0.18	2.73E-03	21.96	5.60E-03
PRRT3	-0.67	0.18	2.73E-03	21.81	5.88E-03
DMC1	0.67	0.18	2.76E-03	20.97	7.82E-03
SYPL1	0.80	0.22	2.76E-03	29.68	3.72E-04
C8orf31	-0.67	0.18	2.76E-03	20.64	8.75E-03
CLDN5	-0.67	0.18	2.79E-03	21.60	6.30E-03
MED11	0.67	0.18	2.79E-03	21.10	7.47E-03
FBXL2	0.67	0.18	2.80E-03	21.49	6.53E-03
WASF1	-0.67	0.18	2.80E-03	21.31	6.94E-03
C2orf42	0.81	0.22	2.83E-03	30.61	2.65E-04
LYRM2	0.67	0.18	2.83E-03	21.37	6.81E-03
CYP4F3	-0.67	0.18	2.84E-03	20.79	8.31E-03
LBH	1.13	0.31	2.84E-03	52.32	6.31E-08
GPR137B	-0.67	0.18	2.84E-03	21.72	6.05E-03
LAPTM4A	0.67	0.18	2.85E-03	21.01	7.71E-03
HIGD1B	-0.67	0.18	2.85E-03	21.37	6.81E-03
MLF1IP	-0.67	0.18	2.85E-03	20.69	8.60E-03
MTRR	0.67	0.18	2.85E-03	21.79	5.92E-03
SNX3	0.67	0.18	2.86E-03	20.36	9.61E-03
UBXN7	-0.71	0.19	2.86E-03	24.51	2.35E-03
RAP1GAP	-0.67	0.18	2.87E-03	21.76	5.98E-03
C20orf26	0.83	0.23	2.88E-03	32.40	1.38E-04
MED14	0.67	0.18	2.88E-03	21.64	6.22E-03
VAPA	0.76	0.21	2.88E-03	27.35	8.68E-04
BRCC3	0.66	0.18	2.92E-03	20.26	9.90E-03
PARVA	0.66	0.18	2.92E-03	21.16	7.33E-03
PTMS	-0.99	0.27	2.92E-03	41.97	3.65E-06
NME5	0.89	0.25	2.92E-03	36.54	2.91E-05
NXNL2	0.68	0.19	2.93E-03	22.75	4.27E-03
DHX58	-0.66	0.18	2.94E-03	20.52	9.08E-03
PIGX	0.66	0.18	2.97E-03	20.86	8.12E-03
DCX	-0.66	0.18	2.97E-03	21.74	6.03E-03
PKIG	0.66	0.18	2.98E-03	21.53	6.44E-03
SP140L	0.73	0.20	3.02E-03	25.87	1.45E-03
PLCG1	-0.94	0.26	3.02E-03	37.99	1.72E-05
DPY30	0.66	0.18	3.03E-03	21.96	5.61E-03
PLEKHA1	0.66	0.18	3.03E-03	20.56	8.97E-03
UBLCP1	0.66	0.18	3.04E-03	20.63	8.79E-03
TTC26	1.08	0.30	3.06E-03	49.41	2.03E-07
DPF3	-1.21	0.33	3.06E-03	55.92	1.57E-08
RPA3	1.13	0.31	3.06E-03	52.57	5.77E-08
MOSPD2	0.66	0.18	3.07E-03	21.13	7.40E-03
PCM1	0.66	0.18	3.07E-03	21.14	7.36E-03
CGREF1	-0.66	0.18	3.09E-03	20.92	7.94E-03

ARHGAP6	0.66	0.18	3.09E-03	20.65	8.73E-03
MTIF3	0.66	0.18	3.09E-03	21.29	7.00E-03
KRT10	0.68	0.19	3.13E-03	22.32	4.95E-03
EIF4ENIF1	-0.84	0.23	3.14E-03	30.71	2.56E-04
LMNB2	-0.66	0.18	3.14E-03	20.49	9.21E-03
FEZ2	0.66	0.18	3.14E-03	20.14	1.03E-02
SPR	0.66	0.18	3.14E-03	21.31	6.93E-03
ARHGEF37	0.66	0.18	3.16E-03	20.60	8.87E-03
LRP2BP	0.71	0.19	3.16E-03	24.59	2.28E-03
SLC25A10	-0.66	0.18	3.16E-03	20.41	9.48E-03
SATB2	0.66	0.18	3.17E-03	20.59	8.91E-03
DGKB	-0.66	0.18	3.18E-03	20.96	7.84E-03
MTHFD1	-0.66	0.18	3.18E-03	21.90	5.69E-03
TPCN2	-0.88	0.24	3.19E-03	34.72	5.83E-05
OFD1	0.66	0.18	3.20E-03	21.64	6.23E-03
BORA	-0.66	0.18	3.20E-03	20.78	8.34E-03
SELT	0.66	0.18	3.20E-03	21.03	7.66E-03
CASC5	-0.66	0.18	3.20E-03	20.91	7.96E-03
MFNG	-0.72	0.20	3.20E-03	25.23	1.82E-03
SLC25A15	-0.82	0.23	3.21E-03	30.55	2.69E-04
TNFRSF11A	-0.66	0.18	3.21E-03	20.48	9.22E-03
HINT2	1.07	0.30	3.21E-03	46.29	6.88E-07
ARHGEF40	-0.66	0.18	3.21E-03	20.46	9.29E-03
ADAM22	0.68	0.19	3.21E-03	23.49	3.33E-03
HAGHL	0.66	0.18	3.23E-03	20.19	1.02E-02
CAMKV	-0.66	0.18	3.23E-03	20.31	9.76E-03
CENPJ	-0.66	0.18	3.23E-03	20.13	1.03E-02
CHRM1	-0.67	0.18	3.23E-03	21.93	5.64E-03
TRMU	-1.00	0.28	3.24E-03	42.40	3.10E-06
MPP3	0.82	0.23	3.25E-03	30.41	2.83E-04
TSPAN6	1.10	0.30	3.26E-03	49.08	2.32E-07
COPZ1	0.66	0.18	3.27E-03	20.71	8.55E-03
MORN5	0.71	0.20	3.27E-03	25.22	1.83E-03
MELK	-0.66	0.18	3.27E-03	21.06	7.58E-03
FZD6	0.66	0.18	3.29E-03	21.40	6.76E-03
ADCK3	-0.66	0.18	3.29E-03	20.05	1.06E-02
NAB2	-0.86	0.24	3.29E-03	33.43	9.35E-05
MECP2	1.07	0.30	3.30E-03	48.48	2.93E-07
PPIL4	0.66	0.18	3.30E-03	21.29	6.99E-03
TSPAN3	0.66	0.18	3.33E-03	20.91	7.96E-03
NTS	0.66	0.18	3.33E-03	21.35	6.85E-03
ABCA5	0.66	0.18	3.34E-03	21.65	6.21E-03
CA8	-0.66	0.18	3.36E-03	20.39	9.54E-03
DEPDC1B	-0.66	0.18	3.36E-03	20.53	9.08E-03
KLHL7	1.20	0.33	3.38E-03	54.70	2.54E-08
TASP1	1.15	0.32	3.38E-03	53.78	3.60E-08
ECHDC1	0.66	0.18	3.39E-03	20.59	8.90E-03
IWS1	0.88	0.24	3.39E-03	34.67	5.92E-05
RBPMS	0.65	0.18	3.41E-03	20.45	9.33E-03
ACSL5	-0.65	0.18	3.43E-03	20.69	8.60E-03
SMPD3	-0.65	0.18	3.44E-03	21.38	6.80E-03
TMX4	0.95	0.27	3.44E-03	40.26	7.03E-06
THSD4	-0.65	0.18	3.44E-03	20.65	8.73E-03
MNS1	1.01	0.28	3.44E-03	44.96	1.15E-06
CCDC23	-0.65	0.18	3.44E-03	20.78	8.34E-03
NLGN4X	0.65	0.18	3.45E-03	21.23	7.13E-03
NTNG2	-0.65	0.18	3.48E-03	21.74	6.01E-03
COL11A1	-0.65	0.18	3.50E-03	21.36	6.84E-03

RPL39L	-0.65	0.18	3.51E-03	20.14	1.03E-02
VDAC3	0.76	0.21	3.51E-03	27.56	8.03E-04
ASF1A	0.65	0.18	3.51E-03	21.25	7.10E-03
RUFY3	-0.65	0.18	3.51E-03	20.11	1.04E-02
KDM4A	-0.72	0.20	3.52E-03	24.87	2.07E-03
MLLT3	0.65	0.18	3.52E-03	21.13	7.40E-03
KLHL32	0.65	0.18	3.53E-03	20.77	8.36E-03
PPP1R12C	-0.65	0.18	3.53E-03	19.36	1.34E-02
KAT2B	0.65	0.18	3.54E-03	21.55	6.40E-03
ZBTB48	-0.65	0.18	3.54E-03	20.21	1.01E-02
WLS	0.65	0.18	3.55E-03	20.62	8.82E-03
MRPL51	0.65	0.18	3.55E-03	20.63	8.79E-03
ADAM33	-0.65	0.18	3.56E-03	20.31	9.78E-03
HDAC4	-0.65	0.18	3.56E-03	20.90	7.99E-03
SYS1	0.90	0.25	3.57E-03	35.60	4.16E-05
DISC1	-0.65	0.18	3.58E-03	20.43	9.40E-03
FAM184A	1.04	0.29	3.58E-03	45.61	8.91E-07
CCDC78	0.65	0.18	3.61E-03	20.28	9.85E-03
FMNL3	-0.82	0.23	3.62E-03	31.60	1.84E-04
B3GNT1	0.65	0.18	3.62E-03	20.40	9.50E-03
BZW2	-0.79	0.22	3.62E-03	29.61	3.82E-04
FAT1	0.65	0.18	3.63E-03	20.52	9.08E-03
TG	-0.65	0.18	3.66E-03	20.80	8.29E-03
FAM60A	0.65	0.18	3.66E-03	19.95	1.10E-02
GPN1	0.88	0.25	3.66E-03	31.54	1.88E-04
RNF149	0.90	0.25	3.66E-03	34.48	6.36E-05
SLC4A4	0.65	0.18	3.66E-03	20.22	1.01E-02
BSN	-0.65	0.18	3.66E-03	20.12	1.04E-02
ENKUR	0.69	0.19	3.68E-03	23.88	2.92E-03
C19orf48	-0.65	0.18	3.70E-03	20.37	9.59E-03
MAPK15	0.65	0.18	3.71E-03	20.30	9.80E-03
ELOVL2	-0.65	0.18	3.72E-03	20.03	1.07E-02
OSTM1	0.65	0.18	3.72E-03	20.28	9.85E-03
TTLL12	-0.72	0.20	3.72E-03	24.76	2.15E-03
MYH7	-0.65	0.18	3.73E-03	20.32	9.74E-03
CD82	-1.01	0.28	3.73E-03	42.73	2.73E-06
CNN3	0.65	0.18	3.74E-03	19.83	1.15E-02
SCN9A	1.16	0.33	3.74E-03	55.93	1.57E-08
SCG3	-0.81	0.23	3.75E-03	30.28	2.97E-04
TARSL2	0.65	0.18	3.76E-03	19.57	1.25E-02
RASD2	-0.65	0.18	3.78E-03	20.56	8.98E-03
FCER1A	0.69	0.19	3.81E-03	23.94	2.86E-03
CCDC110	1.15	0.32	3.81E-03	54.98	2.28E-08
GREB1	-0.73	0.21	3.81E-03	26.19	1.30E-03
OTUB2	-0.65	0.18	3.81E-03	20.39	9.52E-03
EEF1A2	-0.65	0.18	3.82E-03	21.63	6.25E-03
SPTBN5	-0.65	0.18	3.82E-03	19.96	1.10E-02
BIN1	-1.02	0.29	3.83E-03	43.39	2.12E-06
MRPL44	0.65	0.18	3.85E-03	20.69	8.59E-03
TXNDC9	0.65	0.18	3.86E-03	20.78	8.34E-03
CD226	0.65	0.18	3.86E-03	20.19	1.01E-02
CYFIP2	-0.65	0.18	3.86E-03	20.00	1.08E-02
TMEM175	-0.65	0.18	3.87E-03	19.55	1.26E-02
ERI1	0.65	0.18	3.88E-03	19.86	1.14E-02
C7orf63	0.98	0.28	3.91E-03	42.99	2.47E-06
EXOC2	0.65	0.18	3.91E-03	21.08	7.53E-03
GALNT3	0.65	0.18	3.91E-03	20.32	9.74E-03
MFAP2	-0.65	0.18	3.91E-03	20.13	1.03E-02

PPIF	-0.65	0.18	3.93E-03	21.28	7.02E-03
OIP5	-0.65	0.18	3.93E-03	20.56	8.99E-03
ANK3	0.65	0.18	3.95E-03	21.16	7.33E-03
STS	0.65	0.18	3.95E-03	20.78	8.34E-03
NDN	0.84	0.24	3.98E-03	32.60	1.29E-04
RABL5	1.17	0.33	3.98E-03	56.31	1.37E-08
ZNF3	0.65	0.18	3.99E-03	20.68	8.64E-03
KCNQ2	-0.65	0.18	3.99E-03	21.05	7.61E-03
EIF4G1	-0.64	0.18	3.99E-03	19.52	1.27E-02
LRRC34	0.81	0.23	3.99E-03	31.40	1.98E-04
DDAH1	0.65	0.18	4.00E-03	21.01	7.69E-03
PTPRS	-0.69	0.20	4.00E-03	23.75	3.04E-03
EPB41L2	0.64	0.18	4.01E-03	19.73	1.18E-02
COL23A1	-0.67	0.19	4.03E-03	22.68	4.38E-03
RFC5	0.64	0.18	4.06E-03	20.54	9.02E-03
KCTD18	0.64	0.18	4.07E-03	20.34	9.70E-03
SS18	0.64	0.18	4.07E-03	20.91	7.97E-03
ARAP3	-0.64	0.18	4.09E-03	19.93	1.11E-02
NDUFA12	0.64	0.18	4.11E-03	20.43	9.39E-03
LAMA4	-0.72	0.20	4.11E-03	25.78	1.49E-03
NUDCD2	0.69	0.20	4.12E-03	23.52	3.29E-03
CCNO	0.64	0.18	4.13E-03	19.69	1.20E-02
TTC29	0.82	0.23	4.14E-03	31.66	1.80E-04
FBXW2	0.64	0.18	4.14E-03	19.63	1.22E-02
PLCB3	-0.70	0.20	4.16E-03	24.39	2.44E-03
PTPRO	0.64	0.18	4.17E-03	21.46	6.61E-03
AJUBA	0.64	0.18	4.17E-03	19.72	1.19E-02
CD248	-0.64	0.18	4.17E-03	20.12	1.04E-02
CRADD	0.99	0.28	4.17E-03	40.54	6.30E-06
CHCHD1	0.64	0.18	4.18E-03	19.27	1.38E-02
SNX7	0.82	0.23	4.18E-03	31.79	1.72E-04
AP3D1	-0.64	0.18	4.18E-03	19.70	1.19E-02
HDAC9	-0.64	0.18	4.19E-03	20.30	9.80E-03
F2RL1	0.64	0.18	4.21E-03	20.25	9.93E-03
TK1	-0.64	0.18	4.23E-03	20.10	1.04E-02
GATM	0.64	0.18	4.23E-03	20.12	1.04E-02
NOL8	0.64	0.18	4.25E-03	19.73	1.18E-02
POP4	0.90	0.25	4.25E-03	35.60	4.16E-05
CHD3	-1.08	0.31	4.26E-03	49.74	1.78E-07
ITM2A	-0.64	0.18	4.26E-03	21.65	6.20E-03
VASH1	-0.64	0.18	4.26E-03	20.31	9.79E-03
GOLGA2P5	0.64	0.18	4.30E-03	19.95	1.10E-02
RQCD1	0.64	0.18	4.30E-03	20.86	8.10E-03
N6AMT2	0.64	0.18	4.31E-03	20.35	9.63E-03
MLH3	-0.92	0.26	4.31E-03	35.65	4.08E-05
PLXDC1	-0.77	0.22	4.31E-03	28.72	5.26E-04
TBKBP1	-0.71	0.20	4.31E-03	23.95	2.84E-03
COPZ2	0.66	0.19	4.33E-03	22.55	4.58E-03
IPO11	0.96	0.27	4.34E-03	40.26	7.03E-06
LRRC48	0.86	0.25	4.34E-03	34.85	5.55E-05
IL11RA	-0.64	0.18	4.34E-03	20.20	1.01E-02
PARP9	0.64	0.18	4.36E-03	19.30	1.37E-02
IRX5	0.64	0.18	4.37E-03	19.57	1.25E-02
RALB	0.93	0.27	4.37E-03	37.81	1.84E-05
HTATSF1	0.72	0.21	4.38E-03	25.46	1.67E-03
DSCAML1	-0.64	0.18	4.38E-03	19.90	1.12E-02
SORCS3	0.64	0.18	4.38E-03	20.57	8.96E-03
ZC3H6	1.16	0.33	4.40E-03	56.00	1.53E-08

AIMP1	0.64	0.18	4.42E-03	19.58	1.24E-02
GLIS3	0.64	0.18	4.42E-03	20.20	1.01E-02
EML2	-0.64	0.18	4.42E-03	20.77	8.37E-03
LHX2	-0.64	0.18	4.42E-03	20.13	1.03E-02
WNK3	0.64	0.18	4.43E-03	19.34	1.35E-02
PELP1	-0.64	0.18	4.45E-03	19.75	1.18E-02
RNF175	0.64	0.18	4.46E-03	20.00	1.08E-02
GEMIN6	0.64	0.18	4.46E-03	19.79	1.16E-02
C4orf22	0.83	0.24	4.46E-03	32.51	1.33E-04
ABCA2	-0.64	0.18	4.48E-03	19.34	1.35E-02
SDSL	-0.64	0.18	4.49E-03	19.91	1.11E-02
DFNB31	1.04	0.30	4.49E-03	44.75	1.24E-06
FAM13C	0.64	0.18	4.51E-03	19.88	1.13E-02
EFEMP1	0.64	0.18	4.52E-03	19.63	1.22E-02
GKAP1	0.85	0.24	4.53E-03	33.34	9.67E-05
NEDD1	1.19	0.34	4.54E-03	58.15	6.76E-09
ANGPTL4	-0.64	0.18	4.54E-03	19.82	1.15E-02
TIMM23	0.64	0.18	4.56E-03	19.53	1.26E-02
SRRM3	-0.64	0.18	4.56E-03	20.59	8.92E-03
SYCP3	0.64	0.18	4.59E-03	20.17	1.02E-02
VWA3B	0.89	0.25	4.61E-03	36.28	3.20E-05
KCNN2	0.64	0.18	4.62E-03	20.01	1.08E-02
RWDD4	0.83	0.24	4.62E-03	31.99	1.60E-04
LOXL4	1.30	0.37	4.62E-03	63.32	8.00E-10
CLDN16	1.22	0.35	4.63E-03	57.24	9.28E-09
CYP4V2	0.67	0.19	4.63E-03	22.45	4.73E-03
GOLPH3L	0.82	0.23	4.64E-03	31.47	1.94E-04
INTS2	0.64	0.18	4.64E-03	19.33	1.35E-02
GPC1	-0.64	0.18	4.66E-03	19.73	1.18E-02
LMAN2L	0.63	0.18	4.67E-03	19.04	1.49E-02
TMED4	0.84	0.24	4.67E-03	32.30	1.44E-04
FUCA1	0.64	0.18	4.67E-03	19.72	1.19E-02
FAH	-0.63	0.18	4.68E-03	19.01	1.51E-02
PTPLAD1	0.66	0.19	4.69E-03	22.30	4.99E-03
BLCAP	0.64	0.18	4.69E-03	19.65	1.21E-02
CARD8	-0.84	0.24	4.70E-03	32.88	1.16E-04
TGIF1	0.63	0.18	4.75E-03	19.74	1.18E-02
C8orf56	0.63	0.18	4.79E-03	20.38	9.55E-03
EIF3J	0.64	0.18	4.79E-03	20.56	8.97E-03
ABCC8	-0.63	0.18	4.79E-03	19.07	1.48E-02
MREG	0.63	0.18	4.79E-03	19.38	1.33E-02
MEGF10	0.63	0.18	4.80E-03	19.67	1.20E-02
HOXA13	0.99	0.29	4.81E-03	41.74	3.99E-06
C17orf70	-0.63	0.18	4.84E-03	19.15	1.44E-02
XRCC6	-0.63	0.18	4.86E-03	20.06	1.06E-02
SIAE	1.14	0.33	4.86E-03	52.56	5.77E-08
GPT	-0.83	0.24	4.88E-03	30.45	2.79E-04
GCAT	-0.63	0.18	4.89E-03	20.12	1.04E-02
RAB33A	-0.63	0.18	4.91E-03	20.27	9.88E-03
BRWD1	-0.63	0.18	4.91E-03	19.42	1.31E-02
PLEKHH2	-0.63	0.18	4.94E-03	19.23	1.40E-02
MAEL	1.13	0.33	4.96E-03	53.16	4.59E-08
ZNF410	-0.63	0.18	4.98E-03	18.93	1.55E-02
CABP7	-0.63	0.18	4.99E-03	19.97	1.09E-02
PRDM5	0.80	0.23	5.00E-03	30.58	2.68E-04
C5AR1	0.63	0.18	5.01E-03	20.33	9.72E-03
SCUBE2	0.82	0.24	5.01E-03	31.49	1.92E-04
TSHZ2	0.63	0.18	5.02E-03	20.50	9.15E-03

YWHAZ	0.63	0.18	5.03E-03	19.36	1.34E-02
HNRNPF	0.63	0.18	5.05E-03	18.97	1.53E-02
ING2	0.90	0.26	5.06E-03	37.17	2.31E-05
CHRNA10	-0.63	0.18	5.06E-03	18.35	1.86E-02
RNPEP	0.72	0.21	5.07E-03	25.35	1.74E-03
HEBP2	1.13	0.33	5.08E-03	51.58	8.51E-08
HOMER2	-0.63	0.18	5.10E-03	19.39	1.33E-02
FBXO15	0.85	0.24	5.10E-03	33.77	8.29E-05
SLC24A2	0.63	0.18	5.11E-03	20.61	8.85E-03
SLC25A4	0.89	0.26	5.11E-03	36.31	3.16E-05
C9orf72	0.89	0.26	5.11E-03	36.44	3.02E-05
CCDC11	0.88	0.25	5.11E-03	35.87	3.74E-05
NR4A2	0.73	0.21	5.11E-03	26.16	1.31E-03
CKAP2L	-0.63	0.18	5.11E-03	19.06	1.48E-02
EGFL7	-0.63	0.18	5.11E-03	19.58	1.24E-02
HIC2	-1.27	0.37	5.11E-03	57.67	8.13E-09
RALGPS2	1.04	0.30	5.12E-03	47.41	4.50E-07
CCDC14	-0.63	0.18	5.13E-03	19.31	1.36E-02
SPC24	-0.63	0.18	5.14E-03	19.68	1.20E-02
GTPBP10	0.63	0.18	5.16E-03	19.60	1.23E-02
CCPG1	0.85	0.25	5.19E-03	33.72	8.42E-05
ADAMTS10	-0.80	0.23	5.19E-03	30.47	2.78E-04
ARL15	1.33	0.39	5.21E-03	66.63	1.99E-10
DNAI2	0.86	0.25	5.21E-03	34.53	6.25E-05
NUP50	-0.88	0.26	5.22E-03	33.54	9.00E-05
RTEL1	-0.94	0.27	5.22E-03	37.16	2.32E-05
ALPK3	1.08	0.31	5.24E-03	45.70	8.65E-07
AGTRAP	0.63	0.18	5.25E-03	19.82	1.15E-02
CACNA1B	-0.63	0.18	5.25E-03	19.63	1.22E-02
CCNA1	0.88	0.26	5.26E-03	36.06	3.47E-05
RXRG	1.61	0.47	5.26E-03	93.33	0.00E+00
LIMCH1	0.63	0.18	5.27E-03	20.42	9.42E-03
PID1	-0.63	0.18	5.27E-03	19.52	1.27E-02
TMEM161B	-0.63	0.18	5.27E-03	18.40	1.83E-02
TK2	0.63	0.18	5.29E-03	19.11	1.46E-02
ABT1	0.63	0.18	5.29E-03	19.65	1.21E-02
IGHMBP2	-0.63	0.18	5.29E-03	19.68	1.20E-02
APOBEC3C	0.68	0.20	5.30E-03	23.47	3.34E-03
RSPH9	1.01	0.29	5.31E-03	45.33	9.92E-07
TPPP	0.63	0.18	5.32E-03	19.55	1.26E-02
BEX2	0.67	0.19	5.32E-03	23.17	3.70E-03
ENOX2	0.79	0.23	5.33E-03	29.99	3.30E-04
LMOD1	-0.67	0.19	5.37E-03	22.87	4.10E-03
UBP1	-0.63	0.18	5.38E-03	18.45	1.80E-02
DIO3OS	-0.63	0.18	5.38E-03	19.11	1.46E-02
FTSJ1	0.63	0.18	5.38E-03	18.28	1.90E-02
TSNAXIP1	0.63	0.18	5.38E-03	20.18	1.02E-02
EHHADH	0.63	0.18	5.38E-03	19.01	1.51E-02
PTTG1	-0.63	0.18	5.39E-03	19.11	1.46E-02
ITPKA	-0.63	0.18	5.40E-03	20.94	7.90E-03
KLHL29	0.90	0.26	5.41E-03	36.53	2.93E-05
ADSSL1	-0.91	0.27	5.42E-03	36.37	3.09E-05
SMARCB1	-0.63	0.18	5.42E-03	19.13	1.45E-02
SCD	-0.82	0.24	5.43E-03	31.50	1.91E-04
OPTN	0.69	0.20	5.44E-03	23.90	2.90E-03
CFB	-0.62	0.18	5.44E-03	18.93	1.55E-02
TACC2	0.92	0.27	5.44E-03	39.00	1.16E-05
KIF20A	-0.63	0.18	5.45E-03	19.42	1.31E-02

DUT	0.84	0.25	5.46E-03	32.32	1.42E-04
NDFIP2	0.62	0.18	5.46E-03	18.71	1.67E-02
MAST4	0.64	0.19	5.47E-03	21.23	7.14E-03
PRMT3	0.62	0.18	5.48E-03	18.88	1.58E-02
DDX1	0.62	0.18	5.48E-03	18.00	2.08E-02
CXorf22	1.17	0.34	5.51E-03	54.84	2.41E-08
RPH3AL	0.62	0.18	5.53E-03	19.95	1.10E-02
ITGA2	0.62	0.18	5.54E-03	19.30	1.37E-02
ZNF792	-0.62	0.18	5.55E-03	18.84	1.59E-02
DYNLRB2	0.92	0.27	5.57E-03	38.70	1.30E-05
MTMR9	-0.62	0.18	5.57E-03	19.19	1.42E-02
GLT8D2	0.81	0.24	5.58E-03	31.14	2.19E-04
TFCP2L1	-0.62	0.18	5.58E-03	18.86	1.59E-02
SLC16A2	-0.68	0.20	5.60E-03	23.43	3.38E-03
MAML3	0.73	0.21	5.60E-03	26.29	1.25E-03
NAPRT1	-0.62	0.18	5.63E-03	18.65	1.70E-02
VSIG2	-0.62	0.18	5.63E-03	18.28	1.90E-02
HOXD10	0.62	0.18	5.64E-03	20.40	9.50E-03
PPAP2A	0.62	0.18	5.64E-03	19.48	1.29E-02
SMC4	-0.62	0.18	5.65E-03	19.63	1.22E-02
MRPL18	0.62	0.18	5.66E-03	19.69	1.20E-02
R3HDM1	-0.62	0.18	5.66E-03	19.48	1.29E-02
TCEANC	1.00	0.29	5.67E-03	43.67	1.90E-06
GBGT1	-0.62	0.18	5.68E-03	18.81	1.61E-02
LAMC3	-0.62	0.18	5.68E-03	18.99	1.52E-02
OSBPL11	0.62	0.18	5.70E-03	19.09	1.47E-02
SOX2-OT	0.62	0.18	5.70E-03	18.89	1.57E-02
SLC40A1	0.85	0.25	5.72E-03	34.04	7.48E-05
FAM131A	-0.62	0.18	5.72E-03	18.96	1.53E-02
PDLIM2	0.75	0.22	5.75E-03	26.60	1.12E-03
ADRA2A	-0.62	0.18	5.75E-03	18.64	1.70E-02
MBTPS2	0.62	0.18	5.75E-03	19.16	1.43E-02
DEPDC7	-0.62	0.18	5.76E-03	18.54	1.75E-02
STAC2	-0.62	0.18	5.76E-03	20.21	1.01E-02
GNB5	0.83	0.24	5.77E-03	31.80	1.71E-04
MAPK12	-0.62	0.18	5.77E-03	19.27	1.38E-02
UBE2F	0.62	0.18	5.79E-03	19.47	1.29E-02
LGALSL	0.65	0.19	5.79E-03	22.28	5.04E-03
MRPS16	0.62	0.18	5.83E-03	18.84	1.59E-02
UBE2N	0.62	0.18	5.84E-03	18.78	1.63E-02
PROS1	0.62	0.18	5.87E-03	20.27	9.87E-03
ARFIP1	0.73	0.21	5.88E-03	25.92	1.42E-03
ADHFE1	-0.77	0.22	5.90E-03	28.56	5.57E-04
FBXO36	0.84	0.25	5.92E-03	32.56	1.30E-04
UBE2E2	0.62	0.18	5.92E-03	19.79	1.16E-02
NLGN2	-0.69	0.20	5.92E-03	23.79	3.00E-03
PIGA	0.62	0.18	5.92E-03	18.90	1.56E-02
NFYB	1.01	0.30	5.93E-03	44.95	1.15E-06
KIF26B	-0.62	0.18	5.97E-03	20.69	8.60E-03
C11orf88	0.79	0.23	5.98E-03	30.20	3.06E-04
DYNC1H1	-0.62	0.18	5.98E-03	18.68	1.68E-02
TRPC7	0.62	0.18	6.02E-03	18.16	1.97E-02
NFAT5	-0.63	0.19	6.02E-03	20.87	8.08E-03
PITPNB	-0.69	0.20	6.02E-03	22.92	4.03E-03
EFCAB2	0.62	0.18	6.03E-03	19.93	1.11E-02
MMGT1	0.62	0.18	6.06E-03	18.16	1.97E-02
POC5	0.86	0.25	6.09E-03	33.93	7.81E-05
DLGAP5	-0.62	0.18	6.11E-03	19.71	1.19E-02

CDC14A	0.94	0.28	6.12E-03	39.97	7.89E-06
MRPL10	0.68	0.20	6.14E-03	22.78	4.23E-03
ARAP1	-0.62	0.18	6.14E-03	18.23	1.93E-02
AK7	0.90	0.27	6.19E-03	37.63	1.97E-05
HIST1H2BM	-0.62	0.18	6.20E-03	18.34	1.87E-02
SLC30A7	0.71	0.21	6.23E-03	24.74	2.16E-03
RARRES1	-0.62	0.18	6.23E-03	19.36	1.34E-02
CENPBD1	0.93	0.28	6.25E-03	38.80	1.26E-05
MYO1D	0.62	0.18	6.25E-03	18.34	1.86E-02
ARMC4	0.99	0.29	6.26E-03	44.29	1.48E-06
MTIF2	0.62	0.18	6.27E-03	18.98	1.53E-02
CTSZ	0.62	0.18	6.32E-03	19.82	1.15E-02
THOC2	0.62	0.18	6.32E-03	19.16	1.43E-02
LRRIQ1	0.62	0.18	6.33E-03	19.92	1.11E-02
YIPF5	0.85	0.25	6.34E-03	32.69	1.24E-04
DCHS1	-0.89	0.26	6.41E-03	36.44	3.01E-05
POLD1	-0.61	0.18	6.42E-03	17.52	2.43E-02
CCT5	0.61	0.18	6.43E-03	18.64	1.70E-02
AZGP1	-0.61	0.18	6.45E-03	18.90	1.56E-02
MTFR1	0.61	0.18	6.49E-03	19.05	1.49E-02
POLR2H	-0.61	0.18	6.49E-03	17.67	2.31E-02
METRN	0.61	0.18	6.51E-03	18.47	1.79E-02
HOXA10	2.01	0.60	6.51E-03	119.52	0.00E+00
SLC16A1	0.61	0.18	6.52E-03	19.75	1.18E-02
KIF2C	-0.61	0.18	6.55E-03	18.41	1.83E-02
NPTX1	-0.61	0.18	6.56E-03	18.47	1.80E-02
DAZAP2	0.61	0.18	6.57E-03	19.14	1.44E-02
KLHL13	0.77	0.23	6.57E-03	29.23	4.38E-04
AURKA	-0.61	0.18	6.57E-03	18.21	1.94E-02
HOXC13	0.73	0.22	6.58E-03	25.30	1.77E-03
CCDC18	-0.61	0.18	6.58E-03	19.45	1.30E-02
ZNF546	0.61	0.18	6.58E-03	19.21	1.41E-02
SULT4A1	-0.61	0.18	6.58E-03	19.47	1.29E-02
PPM1F	-0.75	0.22	6.59E-03	26.85	1.03E-03
CEP41	1.17	0.35	6.60E-03	57.31	9.14E-09
SLC7A14	-0.61	0.18	6.60E-03	18.45	1.80E-02
DHX40	0.99	0.29	6.61E-03	42.88	2.58E-06
OGG1	-0.61	0.18	6.62E-03	17.97	2.09E-02
COPS8	0.84	0.25	6.66E-03	32.80	1.19E-04
ARHGEF3	-0.61	0.18	6.66E-03	18.44	1.81E-02
MAGEH1	0.89	0.26	6.67E-03	36.10	3.43E-05
BRAP	0.61	0.18	6.67E-03	18.26	1.91E-02
GNG12	0.61	0.18	6.72E-03	17.93	2.12E-02
PKMYT1	-1.11	0.33	6.72E-03	47.40	4.50E-07
CLASP2	-0.61	0.18	6.72E-03	18.58	1.73E-02
VWA5B1	0.74	0.22	6.73E-03	26.90	1.01E-03
B3GALT4	0.61	0.18	6.73E-03	18.46	1.80E-02
SUN1	0.61	0.18	6.73E-03	18.26	1.91E-02
HPRT1	0.75	0.22	6.74E-03	27.71	7.61E-04
FGFR3	-0.75	0.22	6.77E-03	27.32	8.75E-04
CHRNB2	-0.61	0.18	6.78E-03	19.57	1.25E-02
NLRP14	0.61	0.18	6.79E-03	18.53	1.76E-02
SLC9B1	0.76	0.23	6.80E-03	27.73	7.55E-04
PHLDB1	0.81	0.24	6.82E-03	31.01	2.30E-04
SCGN	1.54	0.46	6.82E-03	86.95	3.67E-14
ST6GALNAC6	0.72	0.21	6.82E-03	25.47	1.67E-03
KEAP1	-0.72	0.21	6.85E-03	24.46	2.38E-03
AKT2	-0.61	0.18	6.85E-03	18.28	1.90E-02

WDR96	0.68	0.20	6.87E-03	23.72	3.07E-03
NOS1AP	-0.64	0.19	6.87E-03	21.25	7.08E-03
CYP46A1	-0.61	0.18	6.87E-03	18.72	1.66E-02
HDLBP	0.63	0.19	6.91E-03	20.52	9.08E-03
MEX3B	-0.61	0.18	6.91E-03	18.63	1.71E-02
MCFD2	0.78	0.23	6.96E-03	28.30	6.11E-04
PEX26	-0.61	0.18	6.98E-03	18.53	1.76E-02
RAP1GDS1	-0.61	0.18	7.00E-03	18.17	1.97E-02
ARFGAP2	-0.61	0.18	7.01E-03	18.11	2.00E-02
IQGAP3	-0.61	0.18	7.04E-03	18.34	1.86E-02
CHI3L2	-0.63	0.19	7.05E-03	21.26	7.06E-03
NUDT22	-0.61	0.18	7.07E-03	18.45	1.80E-02
ITPRIPL1	-0.61	0.18	7.08E-03	18.49	1.78E-02
KLHDC10	0.68	0.20	7.08E-03	23.13	3.76E-03
CNNM4	0.61	0.18	7.09E-03	18.56	1.75E-02
LIMS1	0.61	0.18	7.09E-03	19.15	1.44E-02
C21orf59	1.37	0.41	7.10E-03	73.79	1.10E-11
WDR63	0.87	0.26	7.10E-03	35.78	3.87E-05
HPS5	-0.61	0.18	7.10E-03	19.10	1.46E-02
SELRC1	-0.61	0.18	7.12E-03	17.95	2.11E-02
SNX32	-0.75	0.23	7.13E-03	27.23	9.01E-04
LYRM1	1.10	0.33	7.15E-03	50.99	1.09E-07
IP6K1	-0.61	0.18	7.15E-03	18.41	1.82E-02
C9orf116	0.93	0.28	7.17E-03	39.60	9.13E-06
GNS	0.61	0.18	7.17E-03	18.35	1.86E-02
GFPT2	-0.61	0.18	7.19E-03	19.13	1.45E-02
OXGR1	1.09	0.33	7.19E-03	50.97	1.10E-07
LRRTM4	-0.61	0.18	7.19E-03	18.97	1.53E-02
ZNF770	0.66	0.20	7.21E-03	22.48	4.68E-03
SAR1B	0.72	0.22	7.21E-03	24.88	2.06E-03
LRRC55	-0.61	0.18	7.21E-03	18.96	1.53E-02
SLC43A1	-0.61	0.18	7.21E-03	18.34	1.87E-02
CDK17	0.71	0.21	7.22E-03	24.83	2.10E-03
FBXL13	0.90	0.27	7.22E-03	37.41	2.14E-05
GATA6	1.06	0.32	7.23E-03	46.97	5.35E-07
GPR19	0.61	0.18	7.24E-03	18.04	2.05E-02
ERLIN1	0.74	0.22	7.24E-03	26.78	1.05E-03
PNPT1	0.61	0.18	7.24E-03	17.85	2.18E-02
BCAT2	-0.72	0.22	7.24E-03	25.54	1.63E-03
DTL	-0.61	0.18	7.24E-03	19.18	1.43E-02
CELF6	0.61	0.18	7.26E-03	18.18	1.96E-02
EZH1	0.61	0.18	7.26E-03	17.70	2.29E-02
MAU2	-0.61	0.18	7.28E-03	18.60	1.72E-02
TOPORS	0.61	0.18	7.32E-03	18.96	1.53E-02
SCN8A	-0.75	0.23	7.33E-03	27.81	7.35E-04
FAP	-0.61	0.18	7.34E-03	19.25	1.39E-02
CDC27	0.61	0.18	7.36E-03	18.33	1.87E-02
CES3	-0.60	0.18	7.39E-03	17.98	2.09E-02
ARHGAP20	-0.60	0.18	7.39E-03	18.05	2.04E-02
MARK3	-0.69	0.21	7.42E-03	22.55	4.58E-03
DNAJB12	0.61	0.18	7.43E-03	18.41	1.82E-02
JAZF1	0.60	0.18	7.43E-03	18.32	1.88E-02
ADSL	-1.11	0.33	7.43E-03	48.25	3.20E-07
APOLD1	-0.67	0.20	7.47E-03	22.76	4.26E-03
INPPL1	-0.73	0.22	7.47E-03	25.59	1.60E-03
FSD1L	1.04	0.31	7.49E-03	47.98	3.59E-07
CAPZA1	-0.61	0.18	7.50E-03	19.44	1.30E-02
RLBP1	-0.60	0.18	7.50E-03	19.65	1.21E-02

DACT1	-0.60	0.18	7.51E-03	19.36	1.34E-02
CCDC19	0.78	0.24	7.59E-03	29.80	3.56E-04
S100A10	0.60	0.18	7.61E-03	18.10	2.00E-02
PDHA1	0.60	0.18	7.62E-03	18.35	1.86E-02
CYR61	0.79	0.24	7.67E-03	29.51	3.96E-04
CALCOCO2	0.60	0.18	7.68E-03	18.83	1.60E-02
DUS4L	1.31	0.40	7.68E-03	68.14	1.08E-10
SHF	-0.60	0.18	7.68E-03	18.72	1.66E-02
NOTCH1	-0.60	0.18	7.69E-03	18.13	1.99E-02
DNAJA1	0.86	0.26	7.73E-03	34.30	6.80E-05
CCDC8	0.60	0.18	7.73E-03	18.77	1.63E-02
HTT	-0.60	0.18	7.73E-03	18.81	1.61E-02
BMF	-0.60	0.18	7.75E-03	18.32	1.87E-02
NCKAP5	-0.60	0.18	7.75E-03	19.35	1.35E-02
AMZ2P1	0.66	0.20	7.78E-03	21.85	5.79E-03
BLOC1S1	0.60	0.18	7.78E-03	18.39	1.84E-02
BCORL1	-0.60	0.18	7.78E-03	18.59	1.73E-02
LDLRAD3	-0.60	0.18	7.78E-03	18.17	1.96E-02
DZIP1L	0.60	0.18	7.78E-03	17.84	2.19E-02
ST6GAL2	-0.62	0.19	7.79E-03	20.59	8.91E-03
HLTF	0.60	0.18	7.80E-03	18.02	2.06E-02
PRKAR2A	0.60	0.18	7.80E-03	18.54	1.75E-02
UVRAG	-0.60	0.18	7.80E-03	17.49	2.45E-02
TBC1D15	0.73	0.22	7.82E-03	26.12	1.33E-03
TIE1	-0.60	0.18	7.82E-03	18.17	1.97E-02
CCT6B	1.25	0.38	7.84E-03	62.18	1.26E-09
SPTLC1	0.84	0.25	7.84E-03	32.63	1.27E-04
APOO	1.09	0.33	7.86E-03	51.27	9.73E-08
STEAP3	-0.60	0.18	7.89E-03	18.84	1.59E-02
C10orf11	0.97	0.29	7.90E-03	40.26	7.03E-06
ADCY5	-0.60	0.18	7.91E-03	17.72	2.28E-02
PSD3	-0.60	0.18	7.91E-03	17.78	2.23E-02
CYP2C8	0.89	0.27	7.92E-03	36.71	2.75E-05
PDHX	-0.64	0.19	7.93E-03	20.54	9.04E-03
MAFG	-0.60	0.18	7.97E-03	18.28	1.90E-02
TIGD7	0.94	0.28	7.98E-03	39.51	9.47E-06
TCEAL2	0.60	0.18	8.00E-03	18.44	1.81E-02
RBM25	-0.60	0.18	8.00E-03	18.69	1.67E-02
IGFBP5	0.86	0.26	8.01E-03	33.82	8.14E-05
LDOC1	0.74	0.23	8.04E-03	27.41	8.46E-04
TPST1	0.60	0.18	8.05E-03	17.83	2.20E-02
NEB	0.60	0.18	8.06E-03	19.04	1.49E-02
CCDC81	0.94	0.29	8.11E-03	40.85	5.58E-06
HSP90B1	0.60	0.18	8.15E-03	17.40	2.53E-02
ARHGEF6	0.61	0.19	8.16E-03	19.70	1.19E-02
ZMYND10	0.80	0.24	8.17E-03	30.72	2.55E-04
PRRX1	-0.61	0.19	8.18E-03	19.98	1.09E-02
C1orf54	-0.60	0.18	8.18E-03	17.82	2.20E-02
ADCY1	-0.60	0.18	8.20E-03	18.28	1.90E-02
ABCC4	-0.64	0.20	8.24E-03	21.54	6.43E-03
SPG21	0.60	0.18	8.25E-03	17.07	2.81E-02
ASPSCR1	-0.60	0.18	8.25E-03	18.29	1.89E-02
C20orf27	-0.60	0.18	8.25E-03	17.39	2.54E-02
DHRS3	-0.60	0.18	8.25E-03	18.03	2.06E-02
SLC7A1	-1.20	0.36	8.25E-03	59.60	3.71E-09
LPPR2	-0.60	0.18	8.25E-03	17.56	2.40E-02
RBPMS2	-0.60	0.18	8.26E-03	17.86	2.18E-02
LYSMD3	0.60	0.18	8.28E-03	18.71	1.67E-02

CASC1	0.73	0.22	8.28E-03	26.64	1.11E-03
C19orf18	0.60	0.18	8.29E-03	17.91	2.14E-02
DLD	0.91	0.28	8.29E-03	37.21	2.29E-05
ZNF175	0.60	0.18	8.29E-03	18.81	1.61E-02
TIMELESS	-0.60	0.18	8.29E-03	17.58	2.38E-02
POLG	-0.64	0.20	8.29E-03	20.36	9.60E-03
MOB2	-0.60	0.18	8.30E-03	17.25	2.65E-02
CCDC89	0.97	0.29	8.31E-03	41.54	4.27E-06
TBX19	-0.60	0.18	8.32E-03	17.28	2.63E-02
FBXO42	-0.60	0.18	8.33E-03	17.52	2.42E-02
FGF7	0.60	0.18	8.35E-03	18.85	1.59E-02
STOX1	0.90	0.27	8.35E-03	37.64	1.97E-05
TUSC2	0.73	0.22	8.35E-03	26.54	1.15E-03
XPC	0.78	0.24	8.35E-03	28.37	5.96E-04
ZHX1	-0.78	0.24	8.35E-03	28.33	6.06E-04
AKR1C1	0.60	0.18	8.36E-03	17.64	2.34E-02
CYTH2	-0.60	0.18	8.40E-03	17.58	2.38E-02
FLJ26850	1.11	0.34	8.41E-03	52.91	5.02E-08
PGAP2	-0.60	0.18	8.41E-03	18.73	1.66E-02
PDE8B	0.60	0.18	8.43E-03	18.71	1.67E-02
PTPRJ	0.60	0.18	8.43E-03	17.50	2.45E-02
KIF17	0.68	0.21	8.45E-03	23.18	3.69E-03
NOC2L	-0.60	0.18	8.45E-03	17.71	2.28E-02
CDKL2	0.79	0.24	8.46E-03	30.46	2.79E-04
IQCH	0.92	0.28	8.49E-03	39.11	1.11E-05
ARSF	-0.60	0.18	8.49E-03	18.66	1.69E-02
SYNE2	-0.60	0.18	8.49E-03	17.52	2.43E-02
GAS2L2	0.72	0.22	8.50E-03	25.89	1.44E-03
TDRD9	-0.60	0.18	8.55E-03	19.82	1.15E-02
UCK1	0.92	0.28	8.57E-03	37.75	1.89E-05
SERPINB9	0.71	0.22	8.57E-03	25.32	1.76E-03
SMAD1	-0.59	0.18	8.58E-03	17.65	2.32E-02
AMOT	0.73	0.22	8.63E-03	26.51	1.16E-03
TMEM120A	0.62	0.19	8.64E-03	19.83	1.15E-02
ITGA9	-0.59	0.18	8.66E-03	18.54	1.75E-02
TBC1D22A	-0.59	0.18	8.67E-03	18.31	1.88E-02
USP8	0.59	0.18	8.69E-03	18.17	1.97E-02
RNF6	1.08	0.33	8.71E-03	48.97	2.42E-07
CLSTN3	-0.80	0.24	8.73E-03	30.57	2.68E-04
GSG2	-0.59	0.18	8.73E-03	16.91	2.95E-02
CKS2	-0.59	0.18	8.74E-03	18.18	1.96E-02
C18orf8	0.59	0.18	8.74E-03	17.24	2.67E-02
MAOB	0.74	0.23	8.76E-03	27.21	9.07E-04
ZBTB4	0.90	0.27	8.76E-03	35.46	4.38E-05
ACE2	1.38	0.42	8.79E-03	74.44	8.57E-12
SDAD1	0.59	0.18	8.80E-03	17.47	2.47E-02
SCML1	-0.59	0.18	8.80E-03	19.14	1.44E-02
CNPY3	0.59	0.18	8.82E-03	17.20	2.70E-02
SSTR1	-0.59	0.18	8.82E-03	17.89	2.16E-02
STX6	-0.59	0.18	8.83E-03	18.15	1.98E-02
ZMYM5	-0.59	0.18	8.85E-03	18.43	1.81E-02
MED12L	0.59	0.18	8.85E-03	18.49	1.78E-02
NUP210L	0.71	0.22	8.88E-03	24.00	2.79E-03
BAALC	0.59	0.18	8.91E-03	17.89	2.15E-02
CLASRP	-0.78	0.24	8.94E-03	28.10	6.59E-04
SPATA24	0.60	0.19	8.94E-03	19.54	1.26E-02
DNMT1	-0.59	0.18	8.95E-03	17.23	2.67E-02
CACNA1D	0.69	0.21	8.96E-03	24.60	2.27E-03

DYX1C1	1.02	0.31	8.96E-03	46.47	6.45E-07
SAT2	0.59	0.18	8.97E-03	16.97	2.90E-02
TNS1	-0.69	0.21	8.98E-03	23.97	2.83E-03
CEP120	1.09	0.33	9.00E-03	50.67	1.24E-07
EIF2AK2	0.59	0.18	9.00E-03	17.28	2.63E-02
SNCAIP	0.91	0.28	9.00E-03	38.66	1.32E-05
TRABD	-0.93	0.29	9.00E-03	38.44	1.44E-05
PIK3C3	-0.59	0.18	9.01E-03	18.90	1.56E-02
CLIP4	0.59	0.18	9.02E-03	18.22	1.93E-02
SCN2B	-0.59	0.18	9.02E-03	17.23	2.67E-02
SLC7A3	-0.59	0.18	9.05E-03	17.75	2.25E-02
EDDM3A	1.26	0.39	9.09E-03	63.68	6.95E-10
GNG3	-0.59	0.18	9.10E-03	18.37	1.85E-02
LSM5	1.00	0.31	9.11E-03	43.78	1.82E-06
SLC16A10	-0.82	0.25	9.11E-03	32.25	1.46E-04
RIBC2	0.61	0.19	9.12E-03	19.88	1.13E-02
TMEM126B	0.59	0.18	9.13E-03	18.51	1.77E-02
EXT1	0.59	0.18	9.14E-03	17.79	2.22E-02
MCM5	-0.61	0.19	9.14E-03	19.75	1.17E-02
AQP3	-0.59	0.18	9.16E-03	17.16	2.73E-02
DNAJC14	0.59	0.18	9.16E-03	18.22	1.93E-02
FADD	-0.59	0.18	9.18E-03	16.95	2.92E-02
SORCS2	-0.83	0.26	9.22E-03	31.72	1.76E-04
KCNK3	-0.59	0.18	9.22E-03	19.03	1.50E-02
AKAP8L	-0.74	0.23	9.24E-03	26.98	9.81E-04
EP300	-0.59	0.18	9.28E-03	17.39	2.54E-02
LGALS4	-0.60	0.19	9.32E-03	18.76	1.63E-02
TRAM1	0.59	0.18	9.35E-03	18.77	1.63E-02
NKX6-1	1.07	0.33	9.37E-03	48.92	2.46E-07
MOB1A	0.78	0.24	9.41E-03	29.67	3.73E-04
KCNE4	-0.66	0.20	9.42E-03	22.71	4.33E-03
TWF2	-0.59	0.18	9.44E-03	16.92	2.95E-02
COL4A4	0.59	0.18	9.45E-03	18.09	2.02E-02
GABRA5	-0.59	0.18	9.47E-03	19.04	1.49E-02
TICAM1	-0.64	0.20	9.47E-03	20.68	8.63E-03
CDH7	-0.59	0.18	9.48E-03	17.60	2.37E-02
PPTC7	0.59	0.18	9.48E-03	16.89	2.97E-02
REEP2	0.66	0.20	9.50E-03	22.54	4.60E-03
HAUS6	0.73	0.22	9.50E-03	26.32	1.24E-03
GPATCH1	-0.60	0.19	9.51E-03	18.80	1.62E-02
TMEM132A	-0.92	0.29	9.52E-03	38.94	1.19E-05
RPUSD2	0.63	0.19	9.52E-03	20.17	1.02E-02
DSP	0.90	0.28	9.53E-03	37.94	1.75E-05
NDE1	0.83	0.26	9.53E-03	31.51	1.91E-04
KPNA1	0.79	0.25	9.59E-03	28.72	5.25E-04
C9orf37	0.59	0.18	9.67E-03	16.92	2.95E-02
C1orf21	-0.59	0.18	9.67E-03	17.23	2.67E-02
ELAVL3	-0.63	0.19	9.67E-03	21.04	7.64E-03
ICAM5	-0.59	0.18	9.67E-03	16.76	3.09E-02
WRAP53	0.59	0.18	9.67E-03	18.47	1.79E-02
C2orf73	0.73	0.23	9.68E-03	26.83	1.03E-03
COL9A3	-0.81	0.25	9.69E-03	30.87	2.41E-04
ABCG4	-0.59	0.18	9.69E-03	17.82	2.20E-02
RBM39	-0.59	0.18	9.74E-03	17.39	2.54E-02
PON2	0.67	0.21	9.75E-03	23.23	3.63E-03
Mar-03	0.59	0.18	9.79E-03	18.33	1.87E-02
NUP107	0.59	0.18	9.85E-03	18.43	1.81E-02
RSPH3	0.94	0.29	9.86E-03	40.13	7.39E-06

TNNI3	0.59	0.18	9.86E-03	17.30	2.61E-02
UBAC1	0.58	0.18	9.86E-03	16.41	3.47E-02
ARHGAP10	-0.68	0.21	9.87E-03	23.67	3.13E-03
B3GALT6	-0.65	0.20	9.87E-03	21.44	6.66E-03
MTMR9LP	-0.58	0.18	9.89E-03	16.80	3.06E-02
DNAJB6	0.58	0.18	9.90E-03	16.60	3.25E-02
MCOLN3	0.59	0.18	9.91E-03	18.29	1.90E-02
NAF1	0.59	0.18	9.91E-03	17.62	2.36E-02
VPS33A	0.62	0.19	9.92E-03	20.17	1.02E-02
CC2D1A	-0.81	0.25	9.93E-03	30.40	2.84E-04
RAB37	-0.58	0.18	9.93E-03	17.60	2.37E-02
CMC1	0.66	0.21	9.93E-03	22.50	4.65E-03
NKD1	-0.73	0.23	9.93E-03	26.17	1.31E-03
CTIF	-0.65	0.20	9.94E-03	21.61	6.28E-03
UAP1L1	-0.75	0.23	9.96E-03	27.54	8.09E-04
C17orf58	-0.58	0.18	9.99E-03	18.72	1.66E-02
TLE1	-0.58	0.18	1.00E-02	17.74	2.26E-02
TMSB4X	0.62	0.19	1.00E-02	20.69	8.60E-03
AVPR2	-0.58	0.18	1.00E-02	17.04	2.84E-02
RDH5	-0.58	0.18	1.00E-02	16.99	2.89E-02
C5orf64	0.58	0.18	1.00E-02	18.00	2.08E-02
RGL3	-0.61	0.19	1.00E-02	20.29	9.85E-03
CLDN11	-0.58	0.18	1.01E-02	17.76	2.25E-02
NEBL	0.58	0.18	1.01E-02	17.72	2.28E-02
CARKD	-0.58	0.18	1.01E-02	16.62	3.24E-02
ANXA11	0.58	0.18	1.01E-02	18.02	2.06E-02
BCAS4	0.76	0.24	1.01E-02	27.49	8.25E-04
ATP2A2	-0.75	0.24	1.01E-02	27.44	8.39E-04
COL9A2	-0.58	0.18	1.01E-02	17.39	2.54E-02
EID3	0.58	0.18	1.02E-02	17.04	2.84E-02
SALL3	0.58	0.18	1.02E-02	17.16	2.74E-02
TMEM14B	1.14	0.36	1.02E-02	55.16	2.12E-08
EI24	-0.58	0.18	1.02E-02	17.34	2.58E-02
KDSR	0.80	0.25	1.02E-02	30.31	2.93E-04
AVEN	0.74	0.23	1.03E-02	25.70	1.53E-03
RBKS	0.99	0.31	1.03E-02	44.74	1.24E-06
UTP15	0.64	0.20	1.03E-02	21.45	6.64E-03
TEKT3	1.27	0.40	1.04E-02	66.47	2.10E-10
JOSD1	-0.63	0.20	1.04E-02	21.02	7.69E-03
EFHC2	0.81	0.25	1.04E-02	32.18	1.50E-04
ILKAP	-0.58	0.18	1.04E-02	17.34	2.58E-02
SPAG1	0.80	0.25	1.04E-02	31.13	2.19E-04
F7	-0.74	0.23	1.04E-02	25.76	1.50E-03
ZNF417	-0.58	0.18	1.05E-02	17.31	2.60E-02
SPATS2L	0.98	0.31	1.05E-02	42.18	3.36E-06
DRG2	0.58	0.18	1.05E-02	17.90	2.14E-02
SLC35D2	0.58	0.18	1.06E-02	17.75	2.25E-02
WDR60	0.65	0.20	1.06E-02	21.90	5.69E-03
SPATA2L	-0.61	0.19	1.06E-02	19.50	1.28E-02
ACE	-0.64	0.20	1.06E-02	20.58	8.92E-03
BNIP2	0.58	0.18	1.07E-02	17.12	2.77E-02
PFDN1	0.58	0.18	1.07E-02	17.05	2.83E-02
THRB	-0.65	0.20	1.07E-02	21.99	5.54E-03
TRAPPC9	-0.58	0.18	1.07E-02	17.14	2.76E-02
ZNF691	-0.58	0.18	1.07E-02	17.22	2.68E-02
ACVRL1	-0.58	0.18	1.07E-02	17.39	2.54E-02
ERGIC2	0.58	0.18	1.07E-02	16.90	2.96E-02
EIF2S3	0.58	0.18	1.07E-02	16.97	2.90E-02

ULK3	0.58	0.18	1.07E-02	17.34	2.58E-02
ICT1	0.82	0.26	1.08E-02	31.62	1.83E-04
GDF10	-1.15	0.36	1.08E-02	53.37	4.23E-08
TMEM163	0.58	0.18	1.08E-02	17.73	2.27E-02
TTC30B	0.97	0.30	1.08E-02	43.16	2.32E-06
AMDHD1	1.27	0.40	1.08E-02	62.17	1.26E-09
SAMD11	-0.80	0.25	1.08E-02	29.63	3.78E-04
RARS	0.58	0.18	1.08E-02	17.78	2.23E-02
STGC3	-0.58	0.18	1.09E-02	17.45	2.48E-02
CCDC60	1.10	0.35	1.09E-02	52.32	6.31E-08
ANKRD28	0.58	0.18	1.09E-02	18.13	1.99E-02
GABRD	-0.58	0.18	1.09E-02	17.76	2.25E-02
PON1	0.78	0.25	1.09E-02	29.02	4.72E-04
KCNMB2	1.18	0.37	1.09E-02	59.04	4.63E-09
ORAI2	0.58	0.18	1.10E-02	16.93	2.94E-02
IGSF8	-0.58	0.18	1.10E-02	17.50	2.44E-02
TTK	-0.58	0.18	1.10E-02	16.78	3.08E-02
MTMR1	0.58	0.18	1.10E-02	17.04	2.84E-02
MTX1	0.58	0.18	1.10E-02	16.60	3.25E-02
PC	-0.58	0.18	1.10E-02	16.75	3.11E-02
FOXM1	-0.58	0.18	1.10E-02	17.27	2.64E-02
TGFBI	-0.58	0.18	1.11E-02	17.80	2.22E-02
ZNF597	0.58	0.18	1.12E-02	16.42	3.45E-02
CNKSR2	-0.58	0.18	1.12E-02	17.75	2.25E-02
TBC1D1	0.72	0.23	1.13E-02	26.22	1.28E-03
SYNM	0.58	0.18	1.13E-02	17.08	2.80E-02
TRIM14	0.58	0.18	1.13E-02	16.69	3.17E-02
TRIM29	0.83	0.26	1.13E-02	33.54	9.00E-05
PGLS	-0.58	0.18	1.13E-02	16.90	2.97E-02
TRIO	-0.83	0.26	1.13E-02	33.37	9.59E-05
ARHGAP28	-0.61	0.19	1.13E-02	19.96	1.10E-02
MAPK1IP1L	-0.63	0.20	1.13E-02	21.19	7.25E-03
TMEM219	0.61	0.19	1.13E-02	19.49	1.28E-02
NKAPP1	0.80	0.25	1.13E-02	30.43	2.81E-04
MCTS1	0.58	0.18	1.14E-02	17.30	2.61E-02
PODXL	0.58	0.18	1.14E-02	18.91	1.56E-02
TSPYL5	0.97	0.31	1.14E-02	42.73	2.73E-06
BAZ1A	-0.58	0.18	1.14E-02	18.33	1.87E-02
CCDC65	0.89	0.28	1.14E-02	37.71	1.91E-05
NAT14	0.58	0.18	1.15E-02	17.31	2.61E-02
FAM118A	-0.84	0.26	1.15E-02	32.75	1.21E-04
CAPRIN2	0.82	0.26	1.15E-02	29.98	3.31E-04
IQCK	1.14	0.36	1.15E-02	55.81	1.64E-08
KIAA0195	0.71	0.23	1.16E-02	25.36	1.74E-03
PEBP4	-0.57	0.18	1.16E-02	17.00	2.88E-02
KIAA0907	-0.57	0.18	1.16E-02	17.24	2.66E-02
SPEF2	0.68	0.22	1.16E-02	24.12	2.68E-03
MICALL2	-0.69	0.22	1.16E-02	24.18	2.63E-03
LAP3	0.57	0.18	1.17E-02	17.39	2.54E-02
UBXN11	0.57	0.18	1.17E-02	17.62	2.36E-02
SNRPC	0.57	0.18	1.18E-02	17.10	2.79E-02
SLC36A4	0.57	0.18	1.18E-02	16.80	3.06E-02
FERMT2	-0.57	0.18	1.18E-02	18.36	1.86E-02
HMGN5	0.57	0.18	1.18E-02	16.97	2.90E-02
PARP8	0.57	0.18	1.18E-02	16.49	3.37E-02
APBA1	-0.68	0.22	1.19E-02	23.85	2.94E-03
CARHSP1	-0.57	0.18	1.19E-02	17.54	2.42E-02
C15orf26	0.88	0.28	1.19E-02	36.30	3.17E-05

CPSF2	0.57	0.18	1.19E-02	16.51	3.36E-02
NUPL1	-0.69	0.22	1.19E-02	23.67	3.13E-03
SLC25A29	-0.86	0.27	1.19E-02	33.35	9.65E-05
KLHL17	-0.87	0.28	1.19E-02	32.92	1.14E-04
KLKB1	0.62	0.20	1.20E-02	20.36	9.60E-03
RUNDC3A	-0.57	0.18	1.20E-02	16.40	3.47E-02
RASL11A	-0.57	0.18	1.20E-02	16.63	3.23E-02
UACA	0.58	0.18	1.20E-02	18.62	1.71E-02
RPAP1	-0.57	0.18	1.20E-02	16.48	3.39E-02
COX11	0.91	0.29	1.20E-02	37.47	2.09E-05
MMD2	-0.57	0.18	1.21E-02	17.47	2.47E-02
SPA17	0.74	0.23	1.21E-02	27.53	8.11E-04
C22orf15	0.57	0.18	1.21E-02	17.56	2.40E-02
CCDC13	0.57	0.18	1.21E-02	17.55	2.40E-02
WFIKKN2	-0.57	0.18	1.21E-02	17.04	2.84E-02
PHKG1	0.63	0.20	1.21E-02	21.36	6.82E-03
ANGPTL1	-0.57	0.18	1.22E-02	17.55	2.41E-02
F11R	0.57	0.18	1.22E-02	16.76	3.09E-02
SUMO1	0.57	0.18	1.22E-02	15.71	4.32E-02
STMN4	-0.57	0.18	1.22E-02	18.15	1.97E-02
SEC11A	0.79	0.25	1.22E-02	29.60	3.83E-04
TTLL3	-0.57	0.18	1.22E-02	16.49	3.38E-02
PRSS3	-0.63	0.20	1.23E-02	20.94	7.90E-03
CPAMD8	-0.62	0.20	1.23E-02	20.95	7.88E-03
L1CAM	-0.57	0.18	1.23E-02	18.06	2.04E-02
CCDC146	0.86	0.27	1.23E-02	35.34	4.61E-05
RASSF7	-0.57	0.18	1.23E-02	16.65	3.21E-02
CABP4	-0.57	0.18	1.24E-02	17.00	2.88E-02
TP53I11	-0.57	0.18	1.24E-02	17.47	2.47E-02
FCHO1	-0.57	0.18	1.24E-02	17.32	2.60E-02
FXYD2	-0.57	0.18	1.24E-02	16.84	3.02E-02
IRS2	-0.57	0.18	1.25E-02	16.26	3.63E-02
MARCKSL1	-1.06	0.34	1.25E-02	45.41	9.67E-07
AGBL3	0.82	0.26	1.25E-02	32.07	1.55E-04
TOB2	-0.57	0.18	1.25E-02	17.09	2.79E-02
PIEZO1	-0.57	0.18	1.26E-02	17.81	2.21E-02
UBE2L3	-0.57	0.18	1.26E-02	16.47	3.40E-02
TMEM25	-0.84	0.27	1.26E-02	32.20	1.49E-04
AK5	0.79	0.25	1.26E-02	30.89	2.40E-04
ZDHHC17	0.70	0.22	1.26E-02	24.70	2.19E-03
AFF2	-0.65	0.21	1.26E-02	22.24	5.10E-03
COL6A1	-0.57	0.18	1.26E-02	17.85	2.18E-02
SNAPIN	0.57	0.18	1.26E-02	16.13	3.78E-02
EXOC3L2	-0.57	0.18	1.26E-02	16.81	3.05E-02
RNF145	0.57	0.18	1.26E-02	16.59	3.27E-02
RIPK1	0.59	0.19	1.26E-02	18.70	1.67E-02
SLC2A1	-0.97	0.31	1.27E-02	42.55	2.94E-06
ZNF34	-0.57	0.18	1.27E-02	15.61	4.45E-02
CRB2	0.57	0.18	1.27E-02	16.21	3.69E-02
TMEM135	-0.57	0.18	1.27E-02	16.90	2.97E-02
MGC39372	0.57	0.18	1.28E-02	17.80	2.21E-02
CD1D	0.57	0.18	1.28E-02	17.42	2.51E-02
SCN11A	0.68	0.22	1.28E-02	22.89	4.08E-03
TSGA10	0.82	0.26	1.28E-02	33.33	9.69E-05
CDH15	-0.59	0.19	1.28E-02	18.91	1.56E-02
SHB	-0.65	0.21	1.28E-02	21.94	5.64E-03
TMBIM6	0.57	0.18	1.28E-02	16.91	2.95E-02
PELI3	0.57	0.18	1.29E-02	17.53	2.42E-02

KCNB1	0.57	0.18	1.29E-02	16.62	3.24E-02
IGF2BP1	-0.57	0.18	1.29E-02	16.54	3.32E-02
TXLNG	0.95	0.30	1.30E-02	41.12	5.05E-06
ARHGEF16	-0.57	0.18	1.30E-02	16.81	3.05E-02
LXN	0.63	0.20	1.31E-02	21.00	7.73E-03
AVPI1	0.57	0.18	1.31E-02	16.88	2.98E-02
HILPDA	-0.61	0.20	1.31E-02	20.37	9.58E-03
UFSP2	0.61	0.20	1.31E-02	19.49	1.28E-02
DOCK9	-0.57	0.18	1.32E-02	17.38	2.55E-02
PCGF2	0.57	0.18	1.32E-02	16.69	3.17E-02
ALG9	-0.72	0.23	1.32E-02	24.41	2.43E-03
MYO6	-0.60	0.19	1.32E-02	19.80	1.16E-02
TRAF3IP1	1.35	0.43	1.32E-02	72.41	2.02E-11
ANKRA2	0.56	0.18	1.33E-02	16.30	3.58E-02
EIF3L	-0.69	0.22	1.33E-02	23.78	3.00E-03
CCDC57	-0.56	0.18	1.33E-02	16.73	3.13E-02
PIK3R4	0.56	0.18	1.33E-02	15.79	4.22E-02
SLC16A5	0.70	0.22	1.33E-02	24.67	2.21E-03
RLIM	0.62	0.20	1.34E-02	20.46	9.31E-03
GPAT2	-0.56	0.18	1.34E-02	16.92	2.95E-02
MAPK9	0.56	0.18	1.35E-02	16.74	3.11E-02
PBX3	0.60	0.20	1.35E-02	19.96	1.10E-02
HSPB11	0.70	0.23	1.35E-02	25.01	1.97E-03
CAPS2	0.88	0.28	1.35E-02	37.05	2.41E-05
MOB3A	-0.74	0.24	1.35E-02	27.29	8.82E-04
ECEL1	-0.56	0.18	1.36E-02	17.08	2.80E-02
COL20A1	-0.73	0.24	1.36E-02	26.04	1.36E-03
BTG1	-0.60	0.19	1.36E-02	19.58	1.24E-02
FAM194A	0.77	0.25	1.36E-02	29.20	4.43E-04
DHX32	0.56	0.18	1.36E-02	16.39	3.48E-02
FDX1	0.73	0.24	1.36E-02	26.70	1.08E-03
CHST4	0.56	0.18	1.37E-02	15.82	4.18E-02
ARIH1	0.64	0.21	1.38E-02	21.62	6.26E-03
ABCB1	-0.56	0.18	1.38E-02	17.66	2.32E-02
IL4R	-0.58	0.19	1.38E-02	18.53	1.76E-02
FRAS1	1.26	0.41	1.38E-02	60.13	3.01E-09
ZFHX4	0.63	0.20	1.38E-02	21.40	6.76E-03
EPHX1	0.56	0.18	1.38E-02	16.40	3.47E-02
COL6A2	-0.92	0.30	1.39E-02	39.03	1.15E-05
NEURL4	-0.64	0.21	1.39E-02	20.58	8.92E-03
BBX	0.56	0.18	1.39E-02	16.51	3.35E-02
ATP11C	0.76	0.25	1.39E-02	27.94	6.98E-04
COL1A1	-0.56	0.18	1.40E-02	16.47	3.40E-02
PIPOX	-0.56	0.18	1.40E-02	16.71	3.14E-02
TTC25	0.72	0.23	1.40E-02	26.96	9.89E-04
TRIM22	0.56	0.18	1.40E-02	16.93	2.94E-02
IFI27L1	-0.97	0.31	1.41E-02	40.64	6.04E-06
NPTX2	0.56	0.18	1.41E-02	16.27	3.62E-02
CCDC28B	-0.68	0.22	1.42E-02	23.04	3.88E-03
CUTA	0.56	0.18	1.42E-02	16.21	3.69E-02
MARCH10	1.18	0.38	1.42E-02	59.41	4.00E-09
PTPRF	0.76	0.25	1.42E-02	28.58	5.55E-04
SKAP1	0.68	0.22	1.42E-02	23.67	3.13E-03
NME4	0.56	0.18	1.43E-02	16.86	3.00E-02
FAM110A	-0.73	0.24	1.43E-02	25.20	1.84E-03
CCDC41	0.81	0.26	1.43E-02	32.07	1.55E-04
MGAT4A	0.63	0.20	1.44E-02	21.03	7.66E-03
SYT16	0.56	0.18	1.44E-02	17.53	2.42E-02

FAM98B	0.77	0.25	1.44E-02	28.87	4.98E-04
DUSP11	0.75	0.24	1.44E-02	27.46	8.33E-04
CDH10	-0.56	0.18	1.44E-02	16.38	3.49E-02
TRMT112	0.70	0.23	1.45E-02	24.85	2.08E-03
KIFC2	-0.56	0.18	1.45E-02	16.62	3.23E-02
KCTD15	-0.57	0.18	1.45E-02	18.02	2.06E-02
EEF2	-0.56	0.18	1.45E-02	15.74	4.28E-02
DDX26B	0.81	0.26	1.45E-02	32.37	1.39E-04
CDC25B	-0.87	0.28	1.45E-02	36.14	3.38E-05
TSNAX	0.56	0.18	1.45E-02	17.13	2.76E-02
NFATC4	-0.79	0.26	1.45E-02	30.84	2.44E-04
PHF12	0.56	0.18	1.46E-02	16.83	3.03E-02
C6orf62	0.98	0.32	1.46E-02	42.28	3.25E-06
SGK2	-0.56	0.18	1.46E-02	17.25	2.66E-02
GTSE1	-0.68	0.22	1.46E-02	23.62	3.18E-03
PRPF18	0.56	0.18	1.47E-02	16.47	3.40E-02
FAM63A	0.56	0.18	1.47E-02	15.93	4.04E-02
FAM53C	-0.56	0.18	1.47E-02	15.64	4.42E-02
RSPH1	0.74	0.24	1.47E-02	28.18	6.40E-04
NCKAP5L	-0.56	0.18	1.47E-02	15.65	4.41E-02
GPRC5B	-0.56	0.18	1.47E-02	16.20	3.70E-02
MED12	0.56	0.18	1.48E-02	15.47	4.67E-02
EXTL3	0.56	0.18	1.48E-02	16.76	3.10E-02
SULT1A1	-0.56	0.18	1.48E-02	16.19	3.72E-02
SMAD9	-0.56	0.18	1.48E-02	16.95	2.92E-02
SMOC1	-0.99	0.32	1.48E-02	42.06	3.53E-06
DNER	0.56	0.18	1.49E-02	16.08	3.84E-02
MAGED1	0.67	0.22	1.49E-02	23.66	3.14E-03
ZNFX1	-0.56	0.18	1.49E-02	16.72	3.14E-02
FNBP1L	-1.07	0.35	1.49E-02	45.65	8.77E-07
MKS1	0.56	0.18	1.50E-02	15.88	4.10E-02
PIH1D2	0.75	0.24	1.50E-02	28.43	5.83E-04
REEP5	0.56	0.18	1.51E-02	16.62	3.24E-02
SPATA4	1.02	0.33	1.51E-02	47.45	4.43E-07
ATP6AP1	0.56	0.18	1.51E-02	16.83	3.03E-02
GGH	-0.72	0.24	1.51E-02	26.02	1.37E-03
LRRCC1	0.68	0.22	1.52E-02	23.97	2.82E-03
VPS45	-0.56	0.18	1.52E-02	17.00	2.88E-02
EED	-0.55	0.18	1.52E-02	15.99	3.96E-02
MGC45800	-0.55	0.18	1.52E-02	15.97	3.98E-02
ENDOG	0.56	0.18	1.52E-02	16.65	3.21E-02
MRPL43	0.56	0.18	1.52E-02	16.57	3.28E-02
VKORC1L1	0.56	0.18	1.52E-02	15.98	3.96E-02
EFNA3	-0.55	0.18	1.52E-02	15.75	4.26E-02
LMLN	0.64	0.21	1.53E-02	21.92	5.67E-03
SLC9A1	0.62	0.20	1.53E-02	20.10	1.04E-02
MAP3K8	0.55	0.18	1.54E-02	16.77	3.09E-02
SCRG1	0.55	0.18	1.54E-02	15.99	3.96E-02
DOPEY2	0.55	0.18	1.54E-02	16.62	3.23E-02
KAT7	0.55	0.18	1.54E-02	16.41	3.47E-02
POP1	-0.55	0.18	1.54E-02	15.37	4.80E-02
TGOLN2	0.64	0.21	1.54E-02	21.39	6.78E-03
SCD5	0.55	0.18	1.55E-02	15.83	4.17E-02
MET	-0.55	0.18	1.55E-02	16.35	3.52E-02
CELSR3	-0.55	0.18	1.55E-02	16.47	3.40E-02
SPECC1L	-0.55	0.18	1.55E-02	16.20	3.70E-02
HENMT1	-0.55	0.18	1.56E-02	16.74	3.12E-02
CALN1	0.55	0.18	1.56E-02	16.35	3.52E-02

FAM89B	-0.84	0.27	1.56E-02	31.39	1.99E-04
RBMS3	-0.55	0.18	1.56E-02	15.77	4.24E-02
STEAP2	0.61	0.20	1.57E-02	20.40	9.50E-03
TRIM47	-0.55	0.18	1.57E-02	16.84	3.03E-02
DEPDC5	-0.59	0.19	1.57E-02	18.56	1.75E-02
BANF1	0.72	0.24	1.57E-02	26.52	1.16E-03
POLD2	1.00	0.33	1.57E-02	44.91	1.17E-06
DERL2	0.55	0.18	1.58E-02	15.70	4.34E-02
CPEB2	0.55	0.18	1.59E-02	17.02	2.85E-02
CHRNA4	-0.55	0.18	1.59E-02	16.17	3.74E-02
CCDC59	0.73	0.24	1.59E-02	26.98	9.81E-04
GDA	-0.55	0.18	1.59E-02	16.91	2.95E-02
ORC3	0.55	0.18	1.59E-02	16.67	3.19E-02
CPSF3L	-0.92	0.30	1.60E-02	38.52	1.39E-05
CCDC85C	-0.67	0.22	1.61E-02	23.14	3.74E-03
ITM2B	0.68	0.22	1.61E-02	23.65	3.15E-03
CMYA5	1.51	0.50	1.61E-02	84.81	9.84E-14
LRCH1	0.55	0.18	1.61E-02	16.22	3.68E-02
PLD6	0.87	0.29	1.61E-02	34.97	5.32E-05
ATG4C	0.89	0.29	1.61E-02	37.57	2.01E-05
WDR49	0.55	0.18	1.61E-02	16.03	3.90E-02
TAF6L	-0.72	0.24	1.61E-02	25.21	1.84E-03
ANKRD50	-0.70	0.23	1.62E-02	25.54	1.63E-03
UTS2	0.55	0.18	1.62E-02	16.50	3.37E-02
FOXJ1	0.55	0.18	1.63E-02	17.02	2.85E-02
LINC00341	-0.75	0.25	1.63E-02	27.38	8.55E-04
MRPS18A	0.55	0.18	1.64E-02	15.68	4.36E-02
CD69	0.64	0.21	1.64E-02	21.65	6.20E-03
C16orf13	0.55	0.18	1.65E-02	16.46	3.41E-02
PNPLA7	-0.55	0.18	1.66E-02	16.41	3.46E-02
ANKRD16	0.55	0.18	1.66E-02	15.45	4.70E-02
LAMA5	-0.55	0.18	1.66E-02	15.63	4.44E-02
CDH22	-0.83	0.27	1.66E-02	31.85	1.69E-04
ASB1	-0.55	0.18	1.66E-02	15.25	4.99E-02
CADM3	0.55	0.18	1.66E-02	17.27	2.64E-02
TOP3A	-0.55	0.18	1.66E-02	16.12	3.79E-02
ZC3HAV1	0.55	0.18	1.66E-02	16.20	3.70E-02
PNMAL1	0.89	0.30	1.67E-02	38.05	1.68E-05
NPPC	-0.55	0.18	1.67E-02	16.79	3.07E-02
ADAL	0.72	0.24	1.67E-02	26.42	1.20E-03
GFRA1	0.55	0.18	1.67E-02	16.10	3.81E-02
WDR16	0.85	0.28	1.68E-02	35.25	4.77E-05
CHEK2	2.00	0.66	1.69E-02	117.44	0.00E+00
ABCG2	-0.55	0.18	1.71E-02	16.49	3.37E-02
FARP1	-0.55	0.18	1.71E-02	15.98	3.97E-02
TEF	-0.55	0.18	1.71E-02	17.37	2.56E-02
TMEM39B	-0.55	0.18	1.71E-02	15.79	4.21E-02
PCDH20	-0.55	0.18	1.72E-02	17.30	2.61E-02
DLL3	-0.55	0.18	1.72E-02	16.87	3.00E-02
PLAG1	-0.55	0.18	1.72E-02	16.00	3.95E-02
TP53INP2	0.57	0.19	1.72E-02	18.16	1.97E-02
PSTPIP1	-0.55	0.18	1.72E-02	16.13	3.78E-02
MYL5	0.80	0.26	1.72E-02	30.32	2.93E-04
SIGIRR	-0.55	0.18	1.72E-02	16.37	3.51E-02
CNNM3	0.58	0.19	1.72E-02	18.34	1.87E-02
TDP2	0.91	0.30	1.72E-02	38.19	1.58E-05
COLEC11	-0.55	0.18	1.73E-02	16.05	3.88E-02
GIT1	-0.79	0.26	1.73E-02	30.41	2.84E-04

FAM124B	0.81	0.27	1.73E-02	30.96	2.34E-04
AHNAK2	-0.55	0.18	1.73E-02	16.55	3.31E-02
DIS3L	0.64	0.21	1.73E-02	21.59	6.33E-03
PLCH2	-0.57	0.19	1.73E-02	18.36	1.85E-02
IL6	0.69	0.23	1.74E-02	24.68	2.21E-03
ACAP3	-1.08	0.36	1.75E-02	49.70	1.80E-07
KIF5C	0.55	0.18	1.75E-02	16.54	3.32E-02
PAIP2	0.96	0.32	1.76E-02	42.33	3.18E-06
DUS3L	-0.73	0.24	1.76E-02	24.88	2.07E-03
HES4	-0.54	0.18	1.76E-02	15.46	4.69E-02
UBAP1	0.70	0.23	1.77E-02	24.79	2.13E-03
WWP2	0.62	0.21	1.77E-02	20.05	1.06E-02
NUDT11	0.54	0.18	1.77E-02	15.95	4.00E-02
PSMB3	0.70	0.23	1.77E-02	24.52	2.33E-03
NDUFAF1	0.54	0.18	1.78E-02	16.28	3.60E-02
CHD5	-0.54	0.18	1.78E-02	16.09	3.83E-02
COL7A1	-0.66	0.22	1.78E-02	22.63	4.45E-03
PMM1	-0.64	0.21	1.78E-02	21.77	5.97E-03
RASGRP1	-0.54	0.18	1.79E-02	15.63	4.44E-02
CSRNP3	0.54	0.18	1.79E-02	15.43	4.73E-02
SPAG5	-0.69	0.23	1.79E-02	24.72	2.18E-03
ZNF169	-0.66	0.22	1.79E-02	22.35	4.91E-03
UBXN10	0.87	0.29	1.80E-02	36.55	2.91E-05
PDZD11	0.58	0.19	1.80E-02	18.02	2.06E-02
TM2D1	0.66	0.22	1.80E-02	22.67	4.39E-03
DENND4B	-0.74	0.25	1.80E-02	27.32	8.75E-04
TSPAN13	-0.54	0.18	1.80E-02	16.09	3.83E-02
SETDB1	-0.54	0.18	1.81E-02	15.40	4.77E-02
PCDH8	-0.54	0.18	1.81E-02	15.86	4.12E-02
AQP4	-0.54	0.18	1.81E-02	15.76	4.26E-02
GPR146	-0.54	0.18	1.81E-02	16.42	3.45E-02
DAPK2	0.54	0.18	1.81E-02	17.11	2.78E-02
DHFRL1	0.54	0.18	1.81E-02	15.52	4.59E-02
KCTD19	-0.54	0.18	1.81E-02	16.11	3.81E-02
ERCC4	1.04	0.35	1.82E-02	48.35	3.07E-07
PI15	0.54	0.18	1.82E-02	15.29	4.93E-02
KIF27	1.11	0.37	1.83E-02	53.51	4.03E-08
LINC00310	-0.54	0.18	1.83E-02	16.47	3.40E-02
SCAND3	0.72	0.24	1.83E-02	26.14	1.32E-03
GRM1	-0.54	0.18	1.83E-02	15.61	4.46E-02
ST6GAL1	-0.68	0.23	1.84E-02	23.87	2.92E-03
FAM122B	0.54	0.18	1.84E-02	15.38	4.80E-02
ARL13B	0.72	0.24	1.84E-02	26.60	1.13E-03
ZCCHC12	1.04	0.35	1.84E-02	49.17	2.24E-07
IGSF9	-0.54	0.18	1.84E-02	15.47	4.68E-02
GGT7	-0.76	0.25	1.84E-02	28.46	5.76E-04
RPS6KA6	0.54	0.18	1.85E-02	16.53	3.33E-02
TM9SF3	0.54	0.18	1.85E-02	16.72	3.14E-02
HES6	-0.54	0.18	1.85E-02	16.80	3.06E-02
TTLL4	-0.81	0.27	1.86E-02	29.55	3.90E-04
FSIP1	1.13	0.38	1.86E-02	55.54	1.85E-08
SLC25A40	0.54	0.18	1.87E-02	15.98	3.96E-02
AGA	0.90	0.30	1.87E-02	37.72	1.91E-05
CACNA2D2	-0.54	0.18	1.87E-02	15.67	4.38E-02
DZANK1	0.54	0.18	1.88E-02	16.58	3.28E-02
FAM127B	0.54	0.18	1.90E-02	16.34	3.54E-02
ZBBX	0.72	0.24	1.90E-02	27.11	9.41E-04
SLC6A15	-0.54	0.18	1.90E-02	15.58	4.50E-02

CACNA1H	-0.54	0.18	1.90E-02	16.15	3.75E-02
GNB1L	-0.79	0.27	1.91E-02	29.46	4.03E-04
FNTB	-0.54	0.18	1.91E-02	15.27	4.96E-02
GFPT1	0.70	0.23	1.91E-02	25.04	1.95E-03
HEY1	0.64	0.22	1.92E-02	22.16	5.23E-03
WDR66	0.81	0.27	1.92E-02	32.76	1.21E-04
HIPK1	0.64	0.22	1.93E-02	22.05	5.43E-03
ALDH3B1	0.67	0.23	1.93E-02	22.81	4.18E-03
REV1	0.60	0.20	1.93E-02	19.34	1.35E-02
LAMC1	-0.54	0.18	1.94E-02	16.91	2.96E-02
UFM1	0.54	0.18	1.94E-02	15.88	4.10E-02
GPRC5C	0.54	0.18	1.94E-02	15.40	4.77E-02
TM6SF2	-0.63	0.21	1.95E-02	20.73	8.47E-03
ID1	-0.81	0.27	1.95E-02	31.82	1.70E-04
TIGD3	-0.54	0.18	1.95E-02	15.81	4.19E-02
SREBF1	-0.61	0.21	1.97E-02	20.18	1.02E-02
ABHD2	0.59	0.20	1.98E-02	19.33	1.36E-02
EFNA2	-0.66	0.22	1.98E-02	22.78	4.23E-03
ITGB3	0.77	0.26	1.99E-02	29.32	4.25E-04
TOM1L2	0.55	0.19	1.99E-02	17.14	2.76E-02
GPIHBP1	-0.54	0.18	1.99E-02	15.70	4.34E-02
HIST3H2A	-0.56	0.19	1.99E-02	17.90	2.15E-02
WDR73	0.54	0.18	1.99E-02	16.09	3.83E-02
NETO1	-0.57	0.19	1.99E-02	18.19	1.95E-02
PFN4	1.13	0.38	1.99E-02	55.21	2.09E-08
ERGIC3	0.79	0.27	1.99E-02	30.63	2.63E-04
FREM1	0.55	0.19	2.01E-02	17.25	2.66E-02
TTLL6	0.82	0.28	2.01E-02	32.94	1.13E-04
PCNX	-0.54	0.18	2.01E-02	15.28	4.94E-02
LRRC18	0.95	0.32	2.02E-02	42.18	3.36E-06
NGLY1	-0.66	0.22	2.03E-02	21.82	5.86E-03
SPAG17	0.67	0.23	2.03E-02	24.12	2.67E-03
RTKN2	-0.69	0.24	2.04E-02	25.35	1.74E-03
SCN4B	0.70	0.24	2.05E-02	25.76	1.51E-03
SLC37A3	0.53	0.18	2.05E-02	16.11	3.81E-02
KCNN1	-0.53	0.18	2.05E-02	16.57	3.29E-02
GEMIN5	0.53	0.18	2.05E-02	15.80	4.21E-02
DVL2	-0.53	0.18	2.05E-02	15.31	4.90E-02
GLI1	-0.53	0.18	2.06E-02	16.00	3.94E-02
CUL3	0.53	0.18	2.06E-02	15.48	4.65E-02
MARCH2	0.53	0.18	2.06E-02	16.00	3.94E-02
ATP4B	0.98	0.33	2.06E-02	44.23	1.52E-06
PNPLA4	1.18	0.40	2.06E-02	60.36	2.77E-09
IGDCC4	-0.69	0.23	2.07E-02	24.82	2.11E-03
GRHL3	-0.53	0.18	2.07E-02	16.20	3.70E-02
CYB5D1	1.00	0.34	2.07E-02	45.95	7.82E-07
GALR1	0.75	0.25	2.07E-02	27.78	7.42E-04
DHX57	0.80	0.27	2.08E-02	31.31	2.05E-04
LAGE3	0.53	0.18	2.08E-02	15.93	4.04E-02
FCHSD1	-0.53	0.18	2.09E-02	15.64	4.43E-02
POU2F1	-0.53	0.18	2.09E-02	16.40	3.47E-02
SKP1	0.77	0.26	2.09E-02	28.69	5.32E-04
TMEM123	0.93	0.32	2.09E-02	40.65	6.03E-06
IFT46	0.78	0.27	2.10E-02	30.99	2.31E-04
IQCF1	0.53	0.18	2.10E-02	15.74	4.28E-02
DDR2	-1.03	0.35	2.10E-02	46.26	6.97E-07
CREB3L2	0.53	0.18	2.11E-02	16.00	3.95E-02
CHTF18	-0.65	0.22	2.11E-02	21.33	6.91E-03

FAM171B	-0.68	0.23	2.12E-02	23.67	3.13E-03
SPOCK2	-0.53	0.18	2.12E-02	16.39	3.48E-02
PER2	0.70	0.24	2.13E-02	25.60	1.59E-03
CCT2	0.53	0.18	2.14E-02	15.36	4.82E-02
NIP7	0.58	0.20	2.14E-02	18.51	1.77E-02
MMS19	-0.95	0.32	2.15E-02	40.93	5.44E-06
PPP1R15A	0.53	0.18	2.15E-02	15.38	4.79E-02
C2orf44	-0.53	0.18	2.16E-02	15.37	4.81E-02
ALDOC	-0.53	0.18	2.16E-02	15.26	4.98E-02
UBQLN1	0.59	0.20	2.16E-02	19.19	1.42E-02
EMP3	-0.59	0.20	2.16E-02	19.49	1.28E-02
DHRS11	1.23	0.42	2.16E-02	61.82	1.45E-09
PLAA	0.53	0.18	2.17E-02	15.45	4.70E-02
TRMT12	0.84	0.29	2.17E-02	34.47	6.38E-05
FTSJ2	0.62	0.21	2.18E-02	20.47	9.25E-03
GPRASP1	0.56	0.19	2.19E-02	18.04	2.05E-02
BRSK2	-0.57	0.19	2.19E-02	18.18	1.96E-02
KCNRG	1.22	0.42	2.19E-02	63.44	7.68E-10
MCU	0.63	0.22	2.20E-02	21.49	6.54E-03
TAX1BP1	0.67	0.23	2.21E-02	23.91	2.88E-03
HOXA6	1.56	0.54	2.22E-02	91.22	0.00E+00
LRRC71	0.53	0.18	2.23E-02	15.55	4.55E-02
SWAP70	0.53	0.18	2.23E-02	16.25	3.64E-02
MAPRE3	0.53	0.18	2.24E-02	15.41	4.75E-02
RTDR1	0.57	0.20	2.24E-02	18.35	1.86E-02
SNAP25	-0.53	0.18	2.25E-02	15.30	4.91E-02
SYT14	1.50	0.52	2.25E-02	82.29	3.20E-13
PSMA4	0.53	0.18	2.25E-02	15.30	4.91E-02
CBR1	0.82	0.28	2.25E-02	33.66	8.64E-05
BTD	0.67	0.23	2.26E-02	24.05	2.74E-03
CCDC88A	-0.53	0.18	2.26E-02	16.21	3.69E-02
CIRH1A	0.66	0.23	2.26E-02	22.38	4.86E-03
BRPF1	-0.75	0.26	2.27E-02	26.09	1.34E-03
TMEM222	0.53	0.18	2.28E-02	15.32	4.89E-02
ATP6V0E1	0.53	0.18	2.28E-02	15.85	4.15E-02
P2RY2	-0.62	0.21	2.28E-02	20.54	9.03E-03
TLX1	-0.53	0.18	2.29E-02	15.46	4.69E-02
GRIK4	0.86	0.30	2.29E-02	35.50	4.33E-05
KIAA1522	0.64	0.22	2.29E-02	22.20	5.16E-03
FHDC1	-0.53	0.18	2.29E-02	15.88	4.10E-02
PTGFR	0.82	0.28	2.30E-02	32.59	1.29E-04
PHF7	0.63	0.22	2.31E-02	21.69	6.12E-03
CNOT4	0.61	0.21	2.32E-02	20.27	9.89E-03
AGT	0.53	0.18	2.32E-02	16.02	3.91E-02
SLC39A2	1.11	0.38	2.33E-02	53.11	4.70E-08
KDR	-0.79	0.27	2.33E-02	31.58	1.85E-04
SUPT6H	-0.52	0.18	2.33E-02	15.34	4.86E-02
PLXNB3	-0.52	0.18	2.33E-02	15.93	4.03E-02
PKIB	0.58	0.20	2.33E-02	18.57	1.74E-02
TRIOBP	-0.79	0.28	2.34E-02	30.43	2.81E-04
METTL16	0.68	0.24	2.34E-02	24.46	2.38E-03
PLK1	-0.68	0.24	2.35E-02	24.54	2.32E-03
ACP1	0.78	0.27	2.35E-02	30.89	2.40E-04
PPP1R3C	0.73	0.25	2.35E-02	27.55	8.05E-04
ACOT4	-0.52	0.18	2.35E-02	15.60	4.47E-02
SYT6	1.12	0.39	2.36E-02	53.92	3.42E-08
SNTB2	-0.52	0.18	2.38E-02	15.38	4.79E-02
ZFP90	0.52	0.18	2.40E-02	15.82	4.19E-02

CINP	-0.57	0.20	2.40E-02	17.96	2.10E-02
PARK7	0.71	0.25	2.41E-02	26.15	1.32E-03
SHFM1	0.73	0.25	2.41E-02	25.47	1.67E-03
LGALS1	-0.72	0.25	2.41E-02	27.29	8.82E-04
TRMT61A	-0.53	0.19	2.42E-02	15.80	4.21E-02
SOX8	-0.59	0.21	2.42E-02	19.64	1.22E-02
METTL7B	-0.87	0.30	2.44E-02	36.49	2.97E-05
ZNF516	0.55	0.19	2.46E-02	17.58	2.38E-02
RUNDC3B	-0.52	0.18	2.46E-02	15.32	4.88E-02
EPHA7	-0.66	0.23	2.47E-02	23.50	3.31E-03
NUP62CL	0.93	0.33	2.47E-02	41.81	3.89E-06
ZNF408	-0.63	0.22	2.48E-02	20.78	8.34E-03
SGOL2	-0.52	0.18	2.48E-02	15.61	4.46E-02
GTF2H4	-0.65	0.23	2.48E-02	22.24	5.09E-03
TM4SF18	-0.52	0.18	2.49E-02	15.46	4.68E-02
RGS7	0.52	0.18	2.49E-02	15.81	4.19E-02
MSH2	0.55	0.19	2.49E-02	17.37	2.55E-02
PCSK5	0.89	0.31	2.49E-02	36.46	3.00E-05
KIAA0040	0.76	0.27	2.49E-02	29.73	3.65E-04
GK	0.98	0.34	2.50E-02	43.89	1.73E-06
NADK	-0.64	0.22	2.50E-02	21.97	5.57E-03
ELAC1	0.92	0.32	2.51E-02	39.76	8.55E-06
FBP1	0.97	0.34	2.53E-02	41.89	3.75E-06
TCEAL7	0.63	0.22	2.53E-02	21.63	6.24E-03
EXOSC5	-0.59	0.21	2.54E-02	19.28	1.37E-02
PLA2G4A	1.05	0.37	2.54E-02	49.96	1.63E-07
TMBIM1	-0.54	0.19	2.55E-02	16.80	3.06E-02
EFCAB1	0.74	0.26	2.55E-02	28.52	5.64E-04
STK33	0.52	0.18	2.57E-02	15.73	4.30E-02
IRF2	0.71	0.25	2.57E-02	26.11	1.33E-03
FOXRED2	-0.79	0.28	2.57E-02	31.17	2.17E-04
SYP	-0.52	0.18	2.58E-02	15.46	4.69E-02
MTMR10	0.63	0.22	2.58E-02	21.37	6.82E-03
SLC39A6	0.61	0.21	2.60E-02	20.16	1.02E-02
CCNB1IP1	-0.58	0.20	2.61E-02	18.68	1.68E-02
USP6NL	-0.57	0.20	2.62E-02	18.24	1.92E-02
FSIP2	0.73	0.26	2.62E-02	28.07	6.65E-04
AXDND1	0.78	0.28	2.63E-02	31.17	2.16E-04
CSNK1G2	-0.73	0.26	2.63E-02	25.74	1.51E-03
EDIL3	-0.52	0.18	2.64E-02	15.28	4.95E-02
RFC1	0.65	0.23	2.65E-02	22.85	4.13E-03
GAPVD1	0.85	0.30	2.65E-02	32.08	1.55E-04
C17orf72	0.56	0.20	2.66E-02	17.99	2.08E-02
SLC30A5	0.69	0.24	2.66E-02	25.37	1.73E-03
ADD2	0.56	0.20	2.66E-02	18.11	2.00E-02
MIIP	-0.85	0.30	2.67E-02	33.41	9.44E-05
KCTD2	0.69	0.24	2.67E-02	24.78	2.13E-03
GRHPR	0.82	0.29	2.67E-02	31.83	1.70E-04
NAMPT	0.84	0.30	2.67E-02	33.62	8.74E-05
FAM81B	0.79	0.28	2.68E-02	31.76	1.74E-04
C6orf165	0.79	0.28	2.69E-02	31.98	1.60E-04
OR2H1	1.27	0.45	2.70E-02	66.79	1.87E-10
HOGA1	-0.65	0.23	2.70E-02	22.90	4.07E-03
WDR35	0.95	0.33	2.71E-02	42.45	3.04E-06
NRG4	0.96	0.34	2.72E-02	44.07	1.62E-06
FUBP3	0.54	0.19	2.72E-02	16.63	3.23E-02
COQ7	1.30	0.46	2.72E-02	67.95	1.18E-10
BAZ1B	0.70	0.25	2.72E-02	25.79	1.49E-03

LAMB2	-0.58	0.21	2.73E-02	18.65	1.70E-02
GALNT13	0.86	0.30	2.73E-02	36.12	3.40E-05
DLG4	-0.56	0.20	2.73E-02	17.60	2.37E-02
TOX2	1.16	0.41	2.73E-02	56.00	1.53E-08
DST	0.54	0.19	2.75E-02	17.09	2.79E-02
STK32C	-0.51	0.18	2.75E-02	15.56	4.53E-02
MLLT1	0.56	0.20	2.75E-02	17.56	2.40E-02
SLC30A2	-0.51	0.18	2.75E-02	15.36	4.82E-02
LRRC16B	-0.73	0.26	2.76E-02	25.49	1.66E-03
DCDC5	0.57	0.20	2.76E-02	18.45	1.80E-02
BBS9	0.64	0.23	2.76E-02	22.54	4.60E-03
LINC00472	0.97	0.35	2.77E-02	41.37	4.56E-06
ERI3	0.77	0.27	2.77E-02	29.36	4.18E-04
WDR78	0.86	0.31	2.78E-02	37.17	2.31E-05
ELL2	0.51	0.18	2.80E-02	15.53	4.58E-02
ANXA2P1	0.81	0.29	2.80E-02	31.45	1.94E-04
DNAJC17	-0.73	0.26	2.81E-02	26.36	1.22E-03
CFD	0.51	0.18	2.81E-02	15.43	4.73E-02
C7orf61	-0.80	0.28	2.83E-02	31.21	2.12E-04
TMC6	-0.74	0.27	2.83E-02	27.97	6.92E-04
TIMM8A	0.78	0.28	2.83E-02	29.30	4.27E-04
MGAT1	-0.60	0.21	2.84E-02	19.84	1.14E-02
MGST3	1.00	0.36	2.84E-02	44.35	1.44E-06
SIN3A	0.73	0.26	2.84E-02	27.15	9.26E-04
CKB	-0.75	0.27	2.85E-02	27.75	7.50E-04
ST3GAL5	0.80	0.29	2.86E-02	30.79	2.49E-04
PHB	0.81	0.29	2.86E-02	30.25	3.01E-04
RNF122	-0.58	0.21	2.86E-02	18.41	1.82E-02
SNAP91	-0.74	0.26	2.88E-02	28.02	6.79E-04
PWWP2B	-0.60	0.21	2.90E-02	19.94	1.11E-02
BGN	-0.93	0.33	2.90E-02	40.12	7.41E-06
GPR35	-0.66	0.24	2.91E-02	22.04	5.45E-03
NDUFA2	0.78	0.28	2.91E-02	29.07	4.64E-04
KCND2	-0.55	0.20	2.91E-02	17.90	2.15E-02
ADARB1	-0.66	0.24	2.92E-02	23.30	3.54E-03
CAB39	0.61	0.22	2.93E-02	20.71	8.54E-03
UNC119B	0.81	0.29	2.93E-02	33.58	8.87E-05
IRX3	0.55	0.20	2.93E-02	17.53	2.42E-02
ZBTB26	0.63	0.23	2.94E-02	21.47	6.57E-03
FBXL21	-0.53	0.19	2.94E-02	16.88	2.98E-02
DCP1A	-0.58	0.21	2.95E-02	18.61	1.72E-02
ETAA1	0.56	0.20	2.95E-02	17.66	2.32E-02
CTH	-0.61	0.22	2.96E-02	20.35	9.63E-03
MTMR3	-1.00	0.36	3.00E-02	42.57	2.92E-06
PLOD2	0.58	0.21	3.02E-02	19.10	1.46E-02
SHROOM2	-0.65	0.23	3.03E-02	22.50	4.66E-03
CPSF4	0.75	0.27	3.05E-02	26.43	1.20E-03
SRRT	-0.55	0.20	3.07E-02	17.19	2.71E-02
THOC5	-0.90	0.33	3.07E-02	37.90	1.78E-05
CALU	0.63	0.23	3.09E-02	21.30	6.97E-03
SECISBP2L	0.75	0.27	3.09E-02	29.00	4.74E-04
BCAR3	0.89	0.32	3.09E-02	38.51	1.40E-05
UBAP2	0.59	0.21	3.09E-02	19.29	1.37E-02
CLUAP1	1.02	0.37	3.10E-02	48.67	2.72E-07
CUL4B	0.63	0.23	3.11E-02	21.60	6.29E-03
CCNB3	-0.66	0.24	3.12E-02	22.01	5.52E-03
NLGN3	-0.55	0.20	3.13E-02	17.40	2.53E-02
MBNL2	0.74	0.27	3.14E-02	27.79	7.41E-04

FBXL6	-0.64	0.23	3.14E-02	21.38	6.79E-03
TSPAN18	-0.76	0.27	3.15E-02	29.08	4.61E-04
TRAPPC2L	-0.61	0.22	3.15E-02	20.40	9.50E-03
SDHB	0.52	0.19	3.15E-02	15.87	4.11E-02
CYTL1	1.06	0.38	3.16E-02	49.22	2.20E-07
CHCHD5	0.97	0.35	3.16E-02	40.80	5.69E-06
AHCYL1	-0.50	0.18	3.16E-02	15.42	4.74E-02
AGBL2	0.69	0.25	3.16E-02	25.52	1.64E-03
CDHR3	0.97	0.35	3.17E-02	44.89	1.18E-06
CA5B	0.96	0.35	3.17E-02	42.38	3.13E-06
MAMLD1	-0.57	0.21	3.20E-02	18.79	1.62E-02
UBQLN2	0.60	0.22	3.21E-02	20.18	1.02E-02
DEXI	0.52	0.19	3.22E-02	15.77	4.24E-02
DYRK2	0.52	0.19	3.24E-02	15.90	4.08E-02
CLIC4	-0.63	0.23	3.24E-02	22.11	5.33E-03
SRRD	-0.71	0.26	3.25E-02	25.03	1.95E-03
SPIN3	0.63	0.23	3.26E-02	21.15	7.35E-03
RRP8	-0.62	0.22	3.26E-02	20.14	1.03E-02
SH3PXD2B	0.60	0.22	3.27E-02	19.31	1.37E-02
PPP1R1A	-0.50	0.18	3.27E-02	15.50	4.62E-02
SPATA17	0.66	0.24	3.27E-02	23.96	2.84E-03
C1orf50	-0.52	0.19	3.27E-02	15.61	4.46E-02
SLC27A1	0.50	0.18	3.29E-02	15.33	4.87E-02
NASP	-0.64	0.23	3.29E-02	21.52	6.47E-03
CITED4	0.76	0.28	3.29E-02	29.49	3.99E-04
UFL1	0.59	0.22	3.29E-02	19.25	1.39E-02
ARHGAP18	0.71	0.26	3.31E-02	26.78	1.05E-03
TXNDC15	0.80	0.29	3.31E-02	32.75	1.22E-04
NT5DC1	1.19	0.44	3.35E-02	62.33	1.19E-09
NECAB1	0.91	0.33	3.36E-02	39.71	8.70E-06
SCAMP2	0.77	0.28	3.36E-02	29.53	3.94E-04
GLO1	0.54	0.20	3.37E-02	16.85	3.01E-02
PRKCD	-0.85	0.31	3.37E-02	34.68	5.92E-05
CCDC102A	0.59	0.22	3.37E-02	18.67	1.69E-02
C9orf123	1.18	0.43	3.39E-02	59.76	3.51E-09
HIRA	-0.87	0.32	3.40E-02	35.90	3.70E-05
FGGY	0.66	0.24	3.40E-02	23.73	3.06E-03
SSB	0.91	0.33	3.40E-02	40.44	6.57E-06
AKTIP	0.77	0.28	3.40E-02	30.19	3.07E-04
IQSEC1	-0.79	0.29	3.42E-02	29.49	3.99E-04
GPC6	-0.76	0.28	3.47E-02	28.30	6.10E-04
FAM198A	-0.69	0.25	3.49E-02	24.50	2.35E-03
IRF6	0.90	0.33	3.49E-02	39.80	8.41E-06
MBD3	-0.56	0.21	3.49E-02	17.52	2.43E-02
KIAA0101	-0.51	0.19	3.50E-02	15.95	4.00E-02
C11orf1	0.80	0.29	3.50E-02	32.43	1.37E-04
RPS6	0.61	0.23	3.50E-02	20.60	8.87E-03
KCND1	-0.53	0.20	3.50E-02	16.50	3.37E-02
HOXC8	2.28	0.84	3.51E-02	155.79	0.00E+00
DNAJC10	0.81	0.30	3.54E-02	32.70	1.24E-04
ITGB7	0.57	0.21	3.54E-02	18.64	1.70E-02
ZIC4	-1.11	0.41	3.55E-02	50.22	1.47E-07
SEC11C	0.64	0.24	3.57E-02	23.09	3.82E-03
MAGEF1	0.76	0.28	3.57E-02	29.19	4.45E-04
RAD52	-0.60	0.22	3.58E-02	19.75	1.18E-02
ABCF2	0.68	0.25	3.59E-02	24.80	2.12E-03
ACSM3	0.94	0.35	3.62E-02	41.58	4.22E-06
MOCS3	0.96	0.36	3.63E-02	41.97	3.65E-06

TSPAN14	-0.77	0.28	3.63E-02	30.14	3.12E-04
ATL1	0.55	0.20	3.64E-02	17.59	2.38E-02
KLF4	0.59	0.22	3.65E-02	19.75	1.18E-02
PCDH12	-0.56	0.21	3.66E-02	17.94	2.11E-02
NXF3	0.86	0.32	3.68E-02	32.84	1.17E-04
IFT81	0.80	0.30	3.69E-02	33.28	9.89E-05
MLXIP	0.59	0.22	3.70E-02	19.65	1.21E-02
C9orf24	0.81	0.30	3.70E-02	33.89	7.94E-05
ITGA10	-0.53	0.20	3.74E-02	16.27	3.62E-02
PCYT1B	0.53	0.20	3.74E-02	16.54	3.32E-02
GMCL1	0.72	0.27	3.74E-02	26.81	1.04E-03
B3GAT3	-0.58	0.22	3.74E-02	18.94	1.54E-02
RPAP2	-0.59	0.22	3.75E-02	17.91	2.14E-02
ADAP1	-0.59	0.22	3.78E-02	19.67	1.20E-02
SERINC5	-0.76	0.28	3.80E-02	30.18	3.07E-04
SMG9	-0.69	0.26	3.81E-02	24.09	2.70E-03
ATG10	0.52	0.19	3.81E-02	16.09	3.82E-02
RNF216	0.70	0.26	3.81E-02	25.45	1.68E-03
CDK13	0.81	0.30	3.82E-02	33.27	9.95E-05
CACNG6	0.61	0.23	3.82E-02	20.33	9.71E-03
DCT	0.54	0.20	3.82E-02	17.10	2.79E-02
HPR	1.07	0.40	3.83E-02	50.48	1.34E-07
MRPS9	0.62	0.23	3.84E-02	21.12	7.43E-03
PPP2R5A	-0.72	0.27	3.84E-02	26.02	1.37E-03
RUVBL1	0.61	0.23	3.84E-02	20.97	7.80E-03
ZNF610	0.57	0.21	3.85E-02	18.55	1.75E-02
CCDC121	1.07	0.40	3.86E-02	52.55	5.79E-08
WDR65	0.76	0.28	3.90E-02	30.52	2.74E-04
MAPKAP1	0.95	0.36	3.95E-02	42.59	2.90E-06
HP	1.31	0.49	3.95E-02	70.62	4.04E-11
PEX12	0.70	0.26	3.96E-02	25.35	1.74E-03
DHRS7B	0.68	0.26	3.96E-02	23.85	2.95E-03
ZNF680	0.51	0.19	3.97E-02	15.69	4.35E-02
C17orf97	1.01	0.38	4.01E-02	48.44	2.96E-07
DYNC2LI1	1.04	0.39	4.01E-02	47.67	4.04E-07
VAT1	0.59	0.22	4.01E-02	19.47	1.29E-02
VIM	0.89	0.33	4.01E-02	36.21	3.29E-05
CXorf23	0.71	0.27	4.03E-02	26.34	1.23E-03
BBS7	0.62	0.23	4.04E-02	21.63	6.24E-03
HOXC10	1.47	0.55	4.05E-02	85.23	9.84E-14
SQRDL	0.89	0.34	4.06E-02	36.99	2.46E-05
SPRYD7	1.00	0.38	4.10E-02	47.70	4.02E-07
HDC	-0.49	0.19	4.12E-02	15.34	4.85E-02
FGF11	-0.65	0.25	4.13E-02	23.25	3.60E-03
C11orf63	0.70	0.26	4.14E-02	26.70	1.08E-03
ZMAT5	-0.77	0.29	4.14E-02	28.51	5.65E-04
TMED2	0.58	0.22	4.15E-02	19.48	1.29E-02
GHITM	0.62	0.23	4.15E-02	21.22	7.16E-03
CHST12	-0.67	0.25	4.15E-02	23.23	3.63E-03
TOB1	0.77	0.29	4.15E-02	31.45	1.95E-04
PI4KA	-0.76	0.29	4.16E-02	28.55	5.58E-04
FAM135B	1.33	0.50	4.18E-02	70.34	4.48E-11
ZSCAN30	-0.94	0.36	4.18E-02	41.95	3.68E-06
RCAN3	0.57	0.21	4.20E-02	18.81	1.61E-02
MINPP1	0.55	0.21	4.20E-02	17.63	2.34E-02
TMEM5	0.73	0.28	4.22E-02	28.57	5.55E-04
ILVBL	-0.67	0.25	4.22E-02	23.63	3.17E-03
AKAP10	0.57	0.22	4.22E-02	17.78	2.23E-02

CDK5RAP2	0.58	0.22	4.23E-02	19.19	1.42E-02
MAP1S	-0.64	0.24	4.28E-02	21.53	6.45E-03
HNMT	0.67	0.26	4.30E-02	24.35	2.47E-03
ECHDC2	0.57	0.22	4.31E-02	18.84	1.59E-02
PTX3	-0.53	0.20	4.32E-02	16.81	3.05E-02
ALDH6A1	-0.55	0.21	4.33E-02	17.99	2.08E-02
PRSS23	0.59	0.22	4.35E-02	19.82	1.15E-02
VRK2	0.51	0.19	4.36E-02	15.80	4.21E-02
C14orf80	-0.53	0.20	4.39E-02	16.61	3.25E-02
RGS19	-0.73	0.28	4.41E-02	27.34	8.68E-04
MRE11A	0.64	0.24	4.42E-02	22.93	4.03E-03
EXOSC3	0.70	0.27	4.44E-02	25.45	1.68E-03
GUSBP11	-0.51	0.19	4.44E-02	15.42	4.73E-02
KDELC1	-0.67	0.26	4.45E-02	24.95	2.02E-03
ZNF71	-0.72	0.28	4.46E-02	24.90	2.05E-03
CEP19	0.60	0.23	4.46E-02	20.95	7.86E-03
TNC	-0.75	0.29	4.46E-02	30.15	3.12E-04
NRM	-0.60	0.23	4.47E-02	20.54	9.04E-03
DNAH10	0.75	0.29	4.47E-02	29.74	3.65E-04
TP63	1.03	0.39	4.48E-02	46.56	6.22E-07
ISCA1	0.94	0.36	4.49E-02	42.53	2.96E-06
PSME3	0.77	0.30	4.49E-02	29.18	4.46E-04
NUP93	0.90	0.34	4.50E-02	38.52	1.39E-05
ABCC10	-0.69	0.26	4.50E-02	24.78	2.14E-03
NDUFB10	0.97	0.37	4.52E-02	42.66	2.81E-06
PERP	0.81	0.31	4.54E-02	32.91	1.14E-04
OTUD5	0.93	0.36	4.59E-02	41.51	4.32E-06
ABHD10	0.87	0.34	4.63E-02	37.61	1.98E-05
TAS2R4	-0.64	0.25	4.65E-02	23.09	3.82E-03
TMEM47	0.71	0.28	4.68E-02	27.90	7.09E-04
TNIP2	-0.56	0.22	4.69E-02	18.54	1.75E-02
MN1	-0.61	0.23	4.70E-02	20.81	8.26E-03
NUDT9	0.61	0.24	4.72E-02	20.80	8.30E-03
KATNAL2	0.92	0.36	4.74E-02	41.21	4.90E-06
RELL1	0.55	0.21	4.74E-02	17.74	2.26E-02
FLT1	-0.67	0.26	4.75E-02	24.67	2.22E-03
AIMP2	0.82	0.32	4.76E-02	34.25	6.91E-05
GABARAPL2	0.76	0.30	4.77E-02	29.38	4.15E-04
RSAD1	-0.78	0.30	4.77E-02	31.35	2.01E-04
COMMD6	0.53	0.21	4.77E-02	16.79	3.06E-02
PDK3	0.73	0.28	4.79E-02	28.19	6.37E-04
DUSP26	-0.57	0.22	4.80E-02	18.61	1.72E-02
EPB41L5	0.65	0.25	4.84E-02	23.47	3.34E-03
ABHD15	-0.55	0.21	4.84E-02	17.74	2.26E-02
SNRNP70	-0.71	0.28	4.84E-02	26.64	1.11E-03
FBXO16	0.63	0.25	4.85E-02	22.47	4.69E-03
C11orf80	-0.69	0.27	4.86E-02	25.86	1.45E-03
C11orf70	0.81	0.32	4.87E-02	34.36	6.65E-05
MIF	-0.53	0.20	4.87E-02	16.55	3.32E-02
REPIN1	0.70	0.27	4.89E-02	26.27	1.26E-03
EIF2AK1	0.63	0.25	4.90E-02	22.78	4.22E-03
HOXB13	1.23	0.48	4.91E-02	61.91	1.41E-09
ABCC3	0.75	0.29	4.91E-02	28.56	5.57E-04
AMIGO2	0.83	0.33	4.92E-02	36.12	3.40E-05
FOPNL	0.87	0.34	4.92E-02	37.01	2.45E-05
MRPS23	0.76	0.30	4.93E-02	28.56	5.58E-04
HIRIP3	0.58	0.23	4.93E-02	18.96	1.53E-02
PHF14	0.59	0.23	4.95E-02	19.70	1.19E-02

MORN3	0.91	0.35	4.95E-02	41.07	5.13E-06
ARF3	0.51	0.20	4.97E-02	15.64	4.43E-02
ZIC5	-1.36	0.53	5.00E-02	68.94	7.96E-11
