# Supplementary material for: Expression alterations define unique molecular characteristics of spinal ependymomas
Source: Oncotarget. 2015 Mar 30;6(23):19780–91. doi: 10.18632/oncotarget.3715 (PMC4637320; doi:10.18632/oncotarget.3715)
Supplement: Supplementary file 3 [file oncotarget-06-19780-s003.pdf]

Supplementary Table 2. Gene Ontology (GO) enrichment analysis. Results of GO analysis performed on 1,866 up-regulated genes in spinal ependymomas. GO analysis was carried out with biological processes (FDR < 0.05) and the redundant GO terms were removed using ReviGO.

GO ID	GO TERM	N	FDR
GO:0009952	anterior/posterior pattern specification	27	9.37E-10
GO:0006915	apoptotic process	72	6.70E-08
GO:0048704	embryonic skeletal system morphogenesis	15	3.46E-07
GO:0005975	carbohydrate metabolic process	41	4.61E-06
GO:0007049	cell cycle	52	1.68E-05
GO:0000278	mitotic cell cycle	40	3.76E-05
GO:0042384	cilium assembly	13	5.64E-05
GO:0006457	protein folding	28	8.12E-05
GO:0042493	response to drug	38	1.30E-04
GO:0006260	DNA replication	24	2.52E-04
GO:0006120	mitochondrial electron transport, NADH to ubiquinone	10	1.34E-03
GO:0006184	GTP catabolic process	22	1.70E-03
GO:0008283	cell proliferation	35	2.62E-03
GO:0006977	DNA damage response, signal transduction by p53 class mediator resulting in cell cycle arrest	13	3.04E-03
GO:0006979	response to oxidative stress	17	4.72E-03
GO:0034329	cell junction assembly	15	5.01E-03
GO:0060271	cilium morphogenesis	8	5.96E-03
GO:0006869	lipid transport	12	7.36E-03
GO:0007596	blood coagulation	44	7.37E-03
GO:0007155	cell adhesion	51	7.37E-03
GO:0002474	antigen processing and presentation of peptide antigen via MHC class I	15	7.39E-03
GO:0051301	cell division	31	7.65E-03
GO:0043123	positive regulation of I-kappaB kinase/NF-kappaB cascade	19	8.19E-03
GO:0006289	nucleotide-excision repair	12	9.42E-03
GO:0006302	double-strand break repair	11	1.12E-02
GO:0001539	ciliary or flagellar motility	5	1.60E-02
GO:0045454	cell redox homeostasis	10	2.11E-02
GO:0000718	nucleotide-excision repair, DNA damage removal	6	2.16E-02
GO:0030433	ER-associated protein catabolic process	7	3.85E-02
GO:0043410	positive regulation of MAPK cascade	10	4.01E-02
GO:0006508	proteolysis	46	4.06E-02
GO:0010001	glial cell differentiation	5	4.10E-02
GO:0006950	response to stress	18	4.19E-02
GO:0006813	potassium ion transport	18	4.43E-02
