# Supplementary material for: Expression alterations define unique molecular characteristics of spinal ependymomas
Source: Oncotarget. 2015 Mar 30;6(23):19780–91. doi: 10.18632/oncotarget.3715 (PMC4637320; doi:10.18632/oncotarget.3715)
Supplement: Supplementary file 4 [file oncotarget-06-19780-s004.pdf]

Supplementary Table 3. **Enrichment of 34 cellular senescence genes among highly expressed genes in spinal ependymomas**. Genes with pooled effect size (ES) > 0 and significant at FDR < 0.05 by both meta-analysis methods are considered.

	Effect size (ES) based meta-analysis			P-value based meta-analysis	
**Gene**	**ES**	**SE**	**FDR**	**S**	**FDR**
PLA2R1	1.88	0.20	8.18E-18	106.47	0.00E+00
IGF1	1.70	0.20	3.56E-15	92.42	0.00E+00
NR4A3	1.59	0.19	1.63E-13	82.59	2.67E-13
FGF1	1.44	0.19	1.92E-11	70.89	3.62E-11
IGFBP7	1.24	0.19	6.86E-09	55.78	1.66E-08
BLVRA	1.07	0.19	5.82E-07	43.37	2.14E-06
CPEB1	1.01	0.19	3.03E-06	40.59	6.17E-06
GCLM	0.96	0.19	1.05E-05	37.62	1.98E-05
WNT16	1.26	0.26	2.91E-05	59.00	4.71E-09
SOD1	1.42	0.33	3.64E-04	71.18	3.39E-11
ABI3BP	0.78	0.18	3.67E-04	26.73	1.07E-03
SPIN1	1.00	0.24	4.25E-04	41.90	3.75E-06
RAC1	0.77	0.18	4.48E-04	25.98	1.39E-03
C2orf40	0.76	0.18	5.39E-04	26.82	1.04E-03
MDM2	0.74	0.18	8.05E-04	25.20	1.84E-03
CDK6	0.73	0.18	9.97E-04	24.22	2.59E-03
BCL2	0.71	0.18	1.33E-03	23.86	2.93E-03
MECP2	1.07	0.30	3.30E-03	48.48	2.93E-07
ASF1A	0.65	0.18	3.51E-03	21.25	7.10E-03
ING2	0.90	0.26	5.06E-03	37.17	2.31E-05
AGTRAP	0.63	0.18	5.25E-03	19.82	1.15E-02
PNPT1	0.61	0.18	7.24E-03	17.85	2.18E-02
CYR61	0.79	0.24	7.67E-03	29.51	3.96E-04
IGFBP5	0.86	0.26	8.01E-03	33.82	8.14E-05
PCGF2	0.57	0.18	1.32E-02	16.69	3.17E-02
ENDOG	0.56	0.18	1.52E-02	16.65	3.21E-02
CHEK2	2.00	0.66	1.69E-02	117.44	0.00E+00
ERCC4	1.04	0.35	1.82E-02	48.35	3.07E-07
RPS6KA6	0.54	0.18	1.85E-02	16.53	3.33E-02
PER2	0.70	0.24	2.13E-02	25.60	1.59E-03
PPP1R15A	0.53	0.18	2.15E-02	15.38	4.79E-02
AGT	0.53	0.18	2.32E-02	16.02	3.91E-02
MRE11A	0.64	0.24	4.42E-02	22.93	4.03E-03
TP63	1.03	0.39	4.48E-02	46.56	6.22E-07
