# Supplementary material for: Expression alterations define unique molecular characteristics of spinal ependymomas
Source: Oncotarget. 2015 Mar 30;6(23):19780–91. doi: 10.18632/oncotarget.3715 (PMC4637320; doi:10.18632/oncotarget.3715)
Supplement: Supplementary file 5 [file oncotarget-06-19780-s005.pdf]

Supplementary Table 4. Enrichment of chromosomal regions with significantly down-regulated genes by positional gene enrichment (PGE) and gene set enrichment analysis (GSEA) methods. To investigate the possibility of enrichment of differential expression on chromosomes, we performed PGE analysis to detect overrepresented chromosomal regions in the significantly down-regulated 1316 genes in SEPN. The table shows the chromosomal regions that were enriched in these analyses (Number of genes, Hits > 20 and FDR < 0.05). We repeated the enrichment analysis with the GSEA method. Chromosome 22 is indicated in bold.

Positional Gene Enrichment (PGE) Analysis

CHR	Start position	End position	Hits	N	% Enrichment	P	FDR
22	43325255	135720974	81	298	27.18	6.65E-27	1.77E-23
22	324739	32822302	22	64	34.38	4.06E-10	2.07E-09

Gene Set Enrichment Analysis (GSEA)

CHR	Gene set name	# Genes in Overlap (k)	# Genes in Gene Set (K)	% Enrichment	P	FDR
**22**	**chr22q13**	**51**	**235**	**21.7**	**4.33E-30**	**1.41E-27**
19	chr19p13	59	645	9.15	4.27E-15	5.88E-13
**22**	**chr22q12**	**27**	**143**	**18.88**	**5.41E-15**	**5.88E-13**
**22**	**chr22q11**	**40**	**369**	**10.84**	**5.57E-13**	**4.54E-11**
19	chr19q13	59	948	6.22	2.06E-08	1.34E-06
20	chr20q13	25	244	10.25	3.70E-08	2.01E-06
17	chr17q25	23	218	10.55	7.49E-08	3.49E-06
1	chr1p36	36	504	7.14	5.02E-07	2.05E-05
11	chr11q13	28	347	8.07	8.65E-07	3.13E-05
3	chr3p21	23	271	8.49	3.45E-06	1.12E-04

Enrichment of chromosomal regions with *NF2* correlated genes by gene set enrichment analysis (GSEA) method

Gene Set Enrichment Analysis (GSEA)

CHR	Gene set name	# Genes in Overlap (k)	# Genes in Gene Set (K)	% Enrichment	P	FDR
22	chr22q13	34	235	14.47	1.36E-37	4.43E-35
22	chr22q12	12	143	8.39	3.76E-11	6.13E-09
22	chr22q11	14	369	3.79	2.88E-08	3.12E-06
22	chr22q	3	6	50	3.45E-06	2.82E-04
14	chr14q23	5	85	5.88	1.22E-04	7.96E-03
14	chr14q24	6	142	4.23	1.60E-04	8.68E-03
1	chr1q25	5	116	4.31	5.18E-04	2.41E-02
