# Supplementary material for: Expression alterations define unique molecular characteristics of spinal ependymomas
Source: Oncotarget. 2015 Mar 30;6(23):19780–91. doi: 10.18632/oncotarget.3715 (PMC4637320; doi:10.18632/oncotarget.3715)
Supplement: Supplementary file 6 [file oncotarget-06-19780-s006.pdf]

Supplementary Table 5. **Correlation of genes with *NF2* gene expression**. Pearson correlation coefficients of genes with NF2 gene expression were calculated in 3 different studies (Nots, Tor, and Heid) and combined using the DerSimonian-Laird random-effect meta-analytical approach. Correlation coefficients were transformed to Fisher's z-scores and the obtained P values were corrected for multiple testing. Genes with Z-mean of correlation coefficients > 0.4 and FDR < 0.05 were selected.

			Meta-analysis of Correlation coefficient						
**Gene**	**CHR**	**Location**	**Nots**	**Tor**	**Heid**	**Zmean**	**Zse**	**P**	**FDR**
MIEF1	22	22q13	0.62	0.77	0.74	0.91	0.09	6.00E-23	8.34E-19
TCF20	22	22q13.3	0.47	0.61	0.53	0.61	0.06	2.27E-22	1.58E-18
ITPK1	14	14q31	0.52	0.59	0.48	0.60	0.06	1.37E-21	6.35E-18
TTC38	22	22q13	0.48	0.60	0.50	0.60	0.06	4.19E-21	1.46E-17
TTLL12	22	22q13.31	0.56	0.55	0.45	0.58	0.06	2.21E-20	6.14E-17
NOL12	22	22q13.1	0.43	0.56	0.53	0.56	0.06	4.08E-19	9.44E-16
KIAA0930	22	22q13.31	0.47	0.53	0.52	0.56	0.06	6.52E-19	1.29E-15
BZW2	7	7p21.1	0.47	0.57	0.44	0.55	0.06	1.94E-18	3.37E-15
SGSM3	22	22q13.1-q13.2	0.56	0.52	0.39	0.54	0.06	6.72E-18	1.04E-14
BCR	22	22q11.23	0.46	0.57	0.42	0.54	0.06	1.25E-17	1.74E-14
DHTKD1	10	10p14	0.43	0.57	0.45	0.54	0.06	1.86E-17	2.35E-14
C1orf50	1	1p34.2	0.39	0.55	0.50	0.53	0.06	4.32E-17	5.00E-14
PITPNB	22	22q12.1	0.47	0.65	0.64	0.68	0.08	4.74E-17	5.06E-14
SREBF2	22	22q13	0.46	0.52	0.44	0.52	0.06	1.21E-16	1.20E-13
GLTP	12	12q24.11	0.44	0.59	0.44	0.55	0.07	4.68E-16	4.33E-13
BID	22	22q11.1	0.43	0.47	0.51	0.51	0.06	5.83E-16	5.06E-13
ZMAT5	22	22cen-q12.3	0.40	0.49	0.50	0.51	0.06	9.41E-16	7.68E-13
LETM1	4	4p16.3	0.48	0.47	0.43	0.50	0.06	1.22E-15	9.40E-13
RBX1	22	22q13.2	0.40	0.43	0.56	0.50	0.06	2.04E-15	1.49E-12
ZCCHC17	1	1p35.2	0.38	0.52	0.56	0.54	0.07	2.95E-15	2.05E-12
GLRX5	14	14q32.13	0.46	0.40	0.53	0.50	0.06	3.27E-15	2.16E-12
SMARCD3	7	7q35-q36	0.39	0.45	0.53	0.49	0.06	4.13E-15	2.61E-12
RPS19BP1	22	22q13.1	0.48	0.68	0.59	0.68	0.09	1.31E-14	7.94E-12
ESAM	11	11q24.2	0.41	0.50	0.41	0.48	0.06	2.09E-14	1.17E-11
PAG1	8	8q21.13	0.35	0.52	0.50	0.50	0.07	2.11E-14	1.17E-11
DGCR2	22	22q11.21	0.44	0.55	0.64	0.61	0.08	2.25E-14	1.20E-11
FAM89B	11	11q13	0.39	0.45	0.49	0.48	0.06	2.60E-14	1.29E-11
NAV1	1	1q32.3	0.50	0.43	0.41	0.48	0.06	2.52E-14	1.29E-11
PPIA	7	7p13	0.50	0.46	0.36	0.48	0.06	2.77E-14	1.33E-11
USP13	3	3q26.2-q26.3	0.43	0.45	0.44	0.47	0.06	4.20E-14	1.94E-11
ATP2A2	12	12q24.11	0.41	0.43	0.48	0.47	0.06	5.35E-14	2.40E-11
C1orf21	1	1q25	0.36	0.54	0.51	0.52	0.07	5.78E-14	2.43E-11
RGL1	1	1q25.3	0.42	0.56	0.39	0.51	0.07	5.71E-14	2.43E-11
NCAPH2	22	22q13.33	0.36	0.45	0.49	0.47	0.06	8.19E-14	3.34E-11
FOXN3	14	14q31.3	0.39	0.50	0.59	0.54	0.07	9.72E-14	3.86E-11
SSR2	1	1q21-q23	0.33	0.51	0.48	0.48	0.07	1.18E-13	4.54E-11
SLC25A44	1	1q22	0.37	0.51	0.41	0.47	0.06	1.28E-13	4.80E-11

CAPZA1	1	1p13.2	0.35	0.51	0.42	0.46	0.06	1.43E-13	5.22E-11
JARID2	6	6p24-p23	0.38	0.45	0.46	0.46	0.06	2.31E-13	8.23E-11
TOMM40L	1	1q23.3	0.40	0.51	0.34	0.46	0.06	2.78E-13	9.66E-11
PDXK	21	21q22.3	0.42	0.45	0.41	0.46	0.06	2.93E-13	9.91E-11
VPS16	20	20p13	0.51	0.54	0.35	0.52	0.07	3.33E-13	1.10E-10
TBC1D22A	22	22q13.3	0.37	0.45	0.45	0.45	0.06	4.69E-13	1.52E-10
HN1	17	17q25.1	0.33	0.47	0.46	0.45	0.06	5.44E-13	1.72E-10
ACVRL1	12	12q13.13	0.46	0.40	0.42	0.45	0.06	5.62E-13	1.73E-10
MTHFD1	14	14q24	0.47	0.64	0.47	0.60	0.08	7.27E-13	2.20E-10
IL17RB	3	3p21.1	0.36	0.54	0.43	0.49	0.07	8.55E-13	2.53E-10
MTSS1	8	8p22	0.38	0.47	0.40	0.45	0.06	9.15E-13	2.65E-10
FOXRED2	22	22q12.3	0.33	0.49	0.42	0.45	0.06	9.45E-13	2.66E-10
MGAT3	22	22q13.1	0.39	0.43	0.44	0.45	0.06	9.78E-13	2.66E-10
YPEL1	22	22q11.2	0.44	0.36	0.47	0.45	0.06	9.63E-13	2.66E-10
CHDH	3	3p21.1	0.36	0.40	0.50	0.45	0.06	1.14E-12	2.98E-10
DACT3	19	19q13.32	0.47	0.33	0.47	0.45	0.06	1.14E-12	2.98E-10
ZCCHC24	10	10q22.3	0.48	0.41	0.36	0.45	0.06	1.27E-12	3.26E-10
EIF3L	22	22q	0.40	0.60	0.55	0.58	0.08	1.39E-12	3.51E-10
TAB1	22	22q13.1	0.43	0.36	0.47	0.44	0.06	1.82E-12	4.50E-10
DYNC1H1	14	14q32	0.33	0.50	0.40	0.45	0.06	1.90E-12	4.63E-10
H1F0	22	22q13.1	0.38	0.41	0.46	0.44	0.06	2.20E-12	5.28E-10
PMM1	22	22q13.2	0.39	0.47	0.36	0.44	0.06	2.51E-12	5.92E-10
ECE1	1	1p36.1	0.46	0.44	0.31	0.44	0.06	3.29E-12	7.62E-10
ZFAND2B	2	2q35	0.34	0.46	0.42	0.44	0.06	3.65E-12	8.31E-10
CHST1	11	11p11.2	0.42	0.47	0.32	0.43	0.06	4.68E-12	1.05E-09
ACBD6	1	1q25.1	0.32	0.43	0.47	0.43	0.06	5.47E-12	1.20E-09
SAMM50	22	22q13.31	0.43	0.64	0.53	0.61	0.09	5.69E-12	1.24E-09
PXMP2	12	12q24.33	0.33	0.45	0.53	0.47	0.07	6.72E-12	1.43E-09
MARCKSL1	1	1p35.1	0.45	0.48	0.29	0.44	0.07	7.38E-12	1.55E-09
TBC1D7	6	6p24.1	0.43	0.37	0.42	0.43	0.06	8.08E-12	1.68E-09
TST	22	22q13.1	0.39	0.42	0.41	0.43	0.06	8.34E-12	1.70E-09
EDNRA	4	4q31.22	0.35	0.50	0.35	0.44	0.06	9.19E-12	1.85E-09
R3HDM1	2	2q21.3	0.36	0.46	0.38	0.43	0.06	1.02E-11	2.01E-09
MAPT	17	17q21.1	0.37	0.48	0.33	0.43	0.06	1.06E-11	2.08E-09
CGREF1	2	2p23.3	0.38	0.40	0.42	0.42	0.06	1.65E-11	3.19E-09
CDC42BPB	14	14q32.3	0.32	0.48	0.37	0.42	0.06	1.70E-11	3.24E-09
ELOVL2	6	6p24.2	0.34	0.47	0.36	0.42	0.06	1.77E-11	3.32E-09
C1orf54	1	1q21.2	0.39	0.44	0.35	0.42	0.06	2.01E-11	3.71E-09
RAB35	12	12q24.31	0.36	0.55	0.41	0.48	0.07	2.06E-11	3.76E-09
FCHSD2	11	11q13.4	0.37	0.57	0.57	0.57	0.09	2.52E-11	4.51E-09
TMEM206	1	1q32.3	0.33	0.47	0.35	0.41	0.06	3.63E-11	6.38E-09
EFR3B	2	2p23.3	0.38	0.40	0.39	0.41	0.06	3.80E-11	6.59E-09
ZMIZ1	10	10q22.3	0.37	0.42	0.37	0.41	0.06	3.95E-11	6.77E-09
MFNG	22	22q12	0.33	0.35	0.50	0.41	0.06	4.90E-11	8.30E-09
PTN	7	7q33	0.43	0.42	0.30	0.41	0.06	5.06E-11	8.47E-09
ATP1A2	1	1q23.2	0.40	0.41	0.34	0.41	0.06	5.18E-11	8.57E-09

DHCR24	1	1p32.3	0.46	0.49	0.30	0.46	0.07	5.28E-11	8.63E-09
NALCN	13	13q32.3	0.35	0.48	0.30	0.41	0.06	5.56E-11	8.98E-09
SERINC5	5	5q14.1	0.27	0.44	0.44	0.41	0.06	6.24E-11	9.85E-09
UFD1L	22	22q11.21	0.43	0.66	0.58	0.64	0.10	6.24E-11	9.85E-09
MTOR	1	1p36.2	0.36	0.45	0.32	0.41	0.06	7.19E-11	1.11E-08
NTM	11	11q25	0.42	0.41	0.32	0.41	0.06	7.19E-11	1.11E-08
GFOD1	6	6pter-p22.1	0.53	0.35	0.54	0.51	0.08	7.38E-11	1.13E-08
FKBP3	14	14q21.2	0.30	0.50	0.43	0.44	0.07	7.76E-11	1.17E-08
RUNDC3B	7	7q21.12	0.33	0.53	0.44	0.47	0.07	1.03E-10	1.53E-08
SLC22A23	6	6p25.2	0.48	0.30	0.43	0.42	0.07	1.52E-10	2.18E-08
CAMK2D	4	4q26	0.47	0.60	0.38	0.55	0.09	1.66E-10	2.30E-08
DBI	2	2q12-q21	0.36	0.58	0.50	0.53	0.09	2.60E-10	3.26E-08
ACAT2	6	6q25.3	0.37	0.55	0.39	0.48	0.08	3.10E-10	3.81E-08
NDUFA6	22	22q13.2	0.47	0.72	0.65	0.73	0.12	3.86E-10	4.54E-08
SNX6	14	14q13.1	0.38	0.60	0.48	0.54	0.09	5.68E-10	6.32E-08
LZTR1	22	22q11.21	0.39	0.49	0.28	0.42	0.07	1.76E-09	1.59E-07
PREX1	20	20q13.13	0.50	0.58	0.33	0.53	0.09	1.79E-09	1.60E-07
CECR5	22	chr22	0.34	0.52	0.59	0.53	0.09	2.26E-09	1.99E-07
MPST	22	22q13.1	0.56	0.35	0.55	0.53	0.09	2.28E-09	1.99E-07
SLC39A11	17	17q21.31	0.40	0.48	0.27	0.41	0.07	2.71E-09	2.27E-07
PEX26	22	22q11.21	0.35	0.56	0.41	0.49	0.08	4.22E-09	3.21E-07
ZFYVE1	14	14q24.2	0.33	0.56	0.54	0.53	0.09	4.97E-09	3.69E-07
BIN1	2	2q14	0.27	0.42	0.49	0.42	0.07	5.29E-09	3.89E-07
EPAS1	2	2p21-p16	0.31	0.52	0.38	0.44	0.08	5.56E-09	4.04E-07
LAMTOR1	11	11q13.4	0.30	0.51	0.40	0.44	0.08	8.50E-09	5.55E-07
NOSTRIN	2	2q31.1	0.32	0.50	0.32	0.42	0.07	9.36E-09	5.96E-07
TATDN2	3	3p25.3	0.31	0.51	0.36	0.42	0.08	1.35E-08	8.02E-07
C11orf48	11	11q12.3	0.35	0.31	0.51	0.41	0.07	1.71E-08	9.94E-07
TRIM24	7	7q32-q34	0.28	0.46	0.51	0.45	0.08	2.01E-08	1.14E-06
RCC2	1	1p36.13	0.28	0.37	0.50	0.41	0.07	2.05E-08	1.15E-06
SCARB1	12	12q24.31	0.27	0.49	0.43	0.43	0.08	3.10E-08	1.59E-06
CMPK1	1	1p32	0.34	0.59	0.47	0.52	0.10	3.81E-08	1.91E-06
CLPB	11	11q13.4	0.41	0.60	0.38	0.52	0.10	4.69E-08	2.26E-06
TSPAN14	10	10q23.1	0.46	0.54	0.29	0.48	0.09	6.04E-08	2.80E-06
YY1	14	14q	0.31	0.46	0.57	0.49	0.09	6.74E-08	3.06E-06
PPP2R5A	1	1q32.2-q32.3	0.42	0.64	0.43	0.56	0.11	7.28E-08	3.26E-06
TTC13	1	1q42.2	0.31	0.54	0.39	0.45	0.09	7.31E-08	3.26E-06
PYGB	20	20p11.21	0.35	0.53	0.31	0.43	0.08	8.70E-08	3.75E-06
FAM32A	19	19pter-p13.3	0.40	0.59	0.35	0.50	0.10	1.50E-07	5.94E-06
RRP7A	22	22q13.2	0.33	0.55	0.38	0.46	0.09	1.79E-07	6.78E-06
CCNYL1	2	2q33.3	0.28	0.51	0.39	0.43	0.09	2.63E-07	9.30E-06
DHCR7	11	11q13.4	0.27	0.52	0.41	0.43	0.09	2.81E-07	9.82E-06
DCBLD1	6	6q22.1	0.42	0.57	0.31	0.48	0.10	3.09E-07	1.06E-05
ACSS2	20	20q11.22	0.45	0.46	0.23	0.41	0.08	3.89E-07	1.27E-05
MCM5	22	22q13.1	0.32	0.58	0.42	0.48	0.10	3.88E-07	1.27E-05
CYFIP2	5	5q33.3	0.29	0.56	0.46	0.48	0.10	5.12E-07	1.59E-05

FSCN1	7	7p22	0.43	0.47	0.23	0.41	0.08	5.48E-07	1.67E-05
ETFB	19	19q13.3	0.26	0.50	0.43	0.43	0.09	5.61E-07	1.70E-05
BACE1	11	11q23.2-q23.3	0.23	0.44	0.47	0.41	0.09	7.87E-07	2.23E-05
TPMT	6	6p22.3	0.29	0.54	0.39	0.44	0.09	1.05E-06	2.79E-05
KIAA0586	14	14q23.1	0.25	0.50	0.44	0.43	0.09	1.09E-06	2.86E-05
ALDH6A1	14	14q24.3	0.32	0.57	0.38	0.46	0.10	1.18E-06	3.09E-05
GPRC5B	16	16p12	0.28	0.54	0.40	0.45	0.09	1.24E-06	3.23E-05
IDH1	2	2q33.3	0.27	0.54	0.42	0.45	0.10	1.26E-06	3.28E-05
MCCC1	3	3q27	0.24	0.49	0.43	0.42	0.09	1.49E-06	3.76E-05
ROBO4	11	11q24.2	0.54	0.28	0.44	0.45	0.10	1.85E-06	4.46E-05
DRG1	22	22q12.2	0.34	0.65	0.61	0.61	0.13	2.19E-06	5.14E-05
MORC2	22	22q12.2	0.32	0.62	0.51	0.54	0.12	2.21E-06	5.17E-05
STX6	1	1q25.3	0.25	0.53	0.44	0.44	0.10	2.78E-06	6.19E-05
HIF1A	14	14q23.2	0.25	0.52	0.43	0.44	0.10	3.00E-06	6.62E-05
RAP1A	1	1p13.3	0.30	0.55	0.36	0.44	0.10	3.32E-06	7.20E-05
FAM177A1	14	14q13.2	0.24	0.50	0.43	0.42	0.09	3.36E-06	7.25E-05
WDR20	14	14q32.31	0.27	0.55	0.43	0.45	0.10	3.50E-06	7.51E-05
IDI1	10	10p15.3	0.29	0.55	0.36	0.44	0.10	3.58E-06	7.63E-05
ZNF410	14	14q24.3	0.39	0.55	0.27	0.45	0.10	5.57E-06	1.08E-04
PDHA1	X	Xp22.1	0.23	0.50	0.40	0.41	0.09	6.61E-06	1.24E-04
KCTD20	6	6p21.31	0.34	0.60	0.37	0.48	0.11	7.10E-06	1.31E-04
GNPNAT1	14	14q22.1	0.44	0.57	0.26	0.47	0.11	7.49E-06	1.37E-04
RAP2B	3	3q25.2	0.41	0.63	0.34	0.51	0.12	8.03E-06	1.45E-04
COA7	1	1p32.3	0.29	0.57	0.40	0.46	0.11	8.22E-06	1.48E-04
NETO2	16	16q11	0.29	0.56	0.38	0.45	0.10	8.84E-06	1.56E-04
MTMR3	22	22q12.2	0.34	0.69	0.60	0.63	0.15	9.77E-06	1.69E-04
SLC9A9	3	3q24	0.23	0.50	0.42	0.41	0.10	1.12E-05	1.89E-04
LIMA1	12	12q13	0.24	0.54	0.47	0.46	0.11	1.16E-05	1.94E-04
NAPEPLD	7	7q22.1	0.26	0.57	0.48	0.48	0.11	1.26E-05	2.08E-04
TRMU	22	22q13	0.38	0.55	0.27	0.44	0.10	1.26E-05	2.08E-04
ADSL	22	22q13.2	0.44	0.76	0.52	0.69	0.17	1.69E-05	2.66E-04
KEAP1	19	19p13.2	0.24	0.53	0.40	0.42	0.10	2.07E-05	3.14E-04
TIMM9	14	14q21	0.30	0.62	0.47	0.52	0.13	2.24E-05	3.35E-04
CPE	4	4q32.3	0.22	0.51	0.49	0.45	0.11	2.30E-05	3.41E-04
MMD	17	17q	0.34	0.63	0.39	0.50	0.13	2.86E-05	4.07E-04
DRAP1	11	11q13.3	0.27	0.55	0.35	0.43	0.11	3.24E-05	4.47E-04
TMEM184B	22	22q12	0.36	0.34	0.63	0.48	0.12	3.56E-05	4.86E-04
TM4SF18	3	3q25.1	0.36	0.67	0.43	0.55	0.14	3.59E-05	4.89E-04
STARD7	2	2q11.2	0.38	0.57	0.27	0.45	0.11	3.65E-05	4.95E-04
DLST	14	14q24.3	0.40	0.57	0.25	0.45	0.11	4.33E-05	5.64E-04
CBX6	22	22q13.1	0.39	0.63	0.32	0.50	0.13	5.33E-05	6.67E-04
JOSD1	22	22q13.1	0.27	0.64	0.56	0.56	0.14	5.36E-05	6.70E-04
HMGXB4	22	22q13.1	0.26	0.62	0.53	0.53	0.14	5.68E-05	7.03E-04
RAP2A	13	13q34	0.36	0.64	0.36	0.51	0.13	5.74E-05	7.10E-04
PKIA	8	8q21.12	0.39	0.67	0.38	0.55	0.14	6.79E-05	8.17E-04
LSAMP	3	3q13.2-q21	0.25	0.54	0.35	0.41	0.11	6.87E-05	8.24E-04

SACS	13	13q12	0.20	0.47	0.51	0.42	0.11	7.66E-05	9.02E-04
POLR2F	22	22q13.1	0.26	0.64	0.58	0.56	0.15	9.62E-05	1.08E-03
UBE2L3	22	22q11.21	0.45	0.71	0.37	0.59	0.16	1.02E-04	1.13E-03
EIF3D	22	22q13.1	0.27	0.61	0.69	0.61	0.16	1.10E-04	1.20E-03
GPR137B	1	1q42-q43	0.21	0.55	0.46	0.45	0.12	1.14E-04	1.24E-03
PIK3R3	1	1p34.1	0.49	0.51	0.18	0.43	0.12	1.21E-04	1.29E-03
COX16	14	14q24.2	0.19	0.49	0.52	0.44	0.12	1.33E-04	1.41E-03
FAM49B	8	8q24.21	0.22	0.56	0.43	0.45	0.12	1.36E-04	1.43E-03
SNRPD3	22	22q11.23	0.24	0.60	0.59	0.54	0.15	1.41E-04	1.47E-03
CRYL1	13	13q12.11	0.25	0.56	0.36	0.43	0.12	1.41E-04	1.47E-03
DEPDC5	22	22q12.3	0.23	0.58	0.60	0.53	0.15	1.43E-04	1.49E-03
ELOVL6	4	4q25	0.29	0.61	0.36	0.47	0.13	1.44E-04	1.49E-03
IL17RA	22	22q11.1	0.32	0.59	0.30	0.44	0.12	1.52E-04	1.57E-03
HDDC2	6	6q13-q24.3	0.30	0.56	0.29	0.42	0.12	1.55E-04	1.59E-03
ASCC2	22	22q12.1	0.59	0.80	0.38	0.73	0.20	1.79E-04	1.78E-03
THOC5	22	22q12.2	0.41	0.72	0.38	0.58	0.17	2.01E-04	1.96E-03
DAP3	1	1q22	0.17	0.48	0.48	0.41	0.12	2.11E-04	2.04E-03
APEX1	14	14q11.2	0.22	0.55	0.37	0.41	0.12	2.16E-04	2.08E-03
CXorf36	X	Xp11.3	0.22	0.55	0.37	0.41	0.12	2.34E-04	2.22E-03
ARPC2	2	2q36.1	0.30	0.58	0.30	0.43	0.12	2.38E-04	2.25E-03
UBR7	14	14q32.12	0.40	0.69	0.34	0.54	0.16	2.74E-04	2.52E-03
BABAM1	19	19p13.11	0.25	0.59	0.37	0.45	0.13	2.87E-04	2.62E-03
MTMR9	8	8p23-p22	0.23	0.60	0.43	0.47	0.14	2.96E-04	2.69E-03
LRRC40	1	1p31.1	0.19	0.55	0.44	0.43	0.13	3.48E-04	3.05E-03
TCFL5	20	20q13.33	0.20	0.56	0.44	0.44	0.13	3.48E-04	3.05E-03
ABHD4	14	14q11.2	0.20	0.54	0.38	0.41	0.12	3.79E-04	3.28E-03
EIF4ENIF1	22	22q11.2	0.25	0.69	0.61	0.60	0.18	3.93E-04	3.37E-03
PHF5A	22	22q13.2	0.25	0.66	0.50	0.54	0.16	4.31E-04	3.63E-03
HIRA	22	22q11.21	0.22	0.59	0.43	0.46	0.14	4.49E-04	3.75E-03
HPS4	22	22cen-q12.3	0.22	0.41	0.62	0.46	0.14	4.50E-04	3.75E-03
PARP1	1	1q41-q42	0.19	0.57	0.47	0.45	0.14	4.56E-04	3.78E-03
IVNS1ABP	1	1q25.1-q31.1	0.19	0.55	0.43	0.43	0.13	4.65E-04	3.85E-03
MARCKS	6	6q22.2	0.17	0.42	0.54	0.41	0.12	4.68E-04	3.86E-03
CAPZB	1	1p36.1	0.46	0.53	0.16	0.42	0.13	4.77E-04	3.92E-03
TSPAN7	X	Xp11.4	0.19	0.54	0.40	0.41	0.12	4.98E-04	4.05E-03
EIF2B2	14	14q24.3	0.27	0.59	0.32	0.43	0.13	5.98E-04	4.67E-03
RRP1B	21	21q22.3	0.23	0.56	0.32	0.41	0.13	6.68E-04	5.10E-03
ABCB1	7	7q21.12	0.31	0.62	0.29	0.45	0.14	6.77E-04	5.15E-03
LDOC1L	22	22q13.31	0.19	0.59	0.53	0.49	0.15	7.09E-04	5.35E-03
CISD1	10	10q21.1	0.23	0.61	0.39	0.46	0.14	7.22E-04	5.42E-03
FLI1	11	11q24.1-q24.3	0.22	0.57	0.34	0.41	0.13	7.38E-04	5.53E-03
SRRD	22	22q12.1	0.30	0.74	0.51	0.61	0.19	7.59E-04	5.65E-03
PDHB	3	3p21.1-p14.2	0.27	0.63	0.35	0.46	0.15	8.40E-04	6.12E-03
ENOPH1	4	4q21.22	0.22	0.60	0.38	0.44	0.14	8.87E-04	6.40E-03
UBE2D1	10	10q21.1	0.26	0.60	0.32	0.44	0.14	9.03E-04	6.49E-03
PDCL	9	9q12-q13	0.17	0.53	0.58	0.47	0.15	9.06E-04	6.51E-03

DUSP6	12	12q22-q23	0.21	0.58	0.36	0.42	0.14	9.94E-04	7.01E-03
DCTN2	12	12q13.3	0.20	0.60	0.41	0.45	0.14	1.04E-03	7.25E-03
RAP1GDS1	4	4q23-q25	0.21	0.59	0.37	0.43	0.14	1.17E-03	7.98E-03
IMMT	2	2	0.17	0.57	0.43	0.43	0.14	1.30E-03	8.68E-03
EIF2S1	14	14q23.3	0.37	0.71	0.32	0.54	0.18	1.31E-03	8.76E-03
WDR3	1	1p12	0.15	0.54	0.47	0.42	0.14	1.36E-03	8.99E-03
FEZ1	11	11q24.2	0.24	0.60	0.30	0.42	0.14	1.53E-03	9.88E-03
SLC12A2	5	5q23.3	0.17	0.59	0.44	0.44	0.15	1.64E-03	1.04E-02
COMMD9	11	11p13	0.31	0.59	0.22	0.41	0.14	1.74E-03	1.08E-02
AP1B1	22	22q12.2	0.46	0.66	0.20	0.50	0.17	1.76E-03	1.09E-02
ADO	10	10q21.3	0.24	0.65	0.37	0.47	0.16	1.82E-03	1.12E-02
COPS3	17	17p11.2	0.17	0.61	0.45	0.46	0.16	1.82E-03	1.12E-02
PAK1IP1	6	6p24.2	0.17	0.58	0.40	0.42	0.15	1.91E-03	1.17E-02
PNP	14	14q13.1	0.14	0.57	0.45	0.43	0.15	2.20E-03	1.30E-02
EI24	11	11q24	0.22	0.63	0.36	0.45	0.16	2.21E-03	1.30E-02
APPBP2	17	17q23.2	0.13	0.55	0.46	0.42	0.15	2.41E-03	1.39E-02
HECTD3	1	1p34.1	0.41	0.60	0.15	0.43	0.15	2.66E-03	1.51E-02
STX12	1	1p35.3	0.20	0.60	0.32	0.41	0.15	2.78E-03	1.56E-02
ARFGAP3	22	22q13.2	0.17	0.65	0.49	0.50	0.18	2.93E-03	1.62E-02
KLF13	15	15q12	0.31	0.62	0.23	0.43	0.16	2.96E-03	1.63E-02
ATP6V1E1	22	22q11.1	0.17	0.66	0.51	0.51	0.19	3.12E-03	1.70E-02
ENTPD5	14	14q24	0.48	0.59	0.12	0.45	0.17	3.62E-03	1.91E-02
KIAA0141	5	5q31.3	0.26	0.63	0.28	0.44	0.16	3.76E-03	1.97E-02
DHRS7	14	14q23.1	0.15	0.60	0.41	0.43	0.16	3.88E-03	2.02E-02
PRKCH	14	14q23.1	0.26	0.62	0.26	0.42	0.16	3.88E-03	2.02E-02
GPR116	6	6p12.3	0.25	0.64	0.27	0.44	0.17	4.71E-03	2.34E-02
UQCR10	22	22q12.2	0.13	0.65	0.59	0.53	0.20	4.85E-03	2.39E-02
TUG1	22	22q12.2	0.12	0.62	0.67	0.55	0.22	5.18E-03	2.50E-02
PRR14L	22	22q12.2	0.28	0.66	0.27	0.45	0.18	5.42E-03	2.60E-02
DGCR14	22	22q11.21	0.17	0.69	0.44	0.50	0.20	6.55E-03	2.99E-02
GPHN	14	14q23.3	0.20	0.64	0.31	0.43	0.17	6.63E-03	3.02E-02
ATXN10	22	22q13.31	0.34	0.77	0.33	0.58	0.24	7.11E-03	3.19E-02
HMOX2	16	16p13.3	0.19	0.63	0.31	0.42	0.17	7.30E-03	3.26E-02
UBE2G1	17	17p13.2	0.16	0.62	0.35	0.42	0.17	7.69E-03	3.39E-02
TINF2	14	14q12	0.09	0.61	0.47	0.44	0.18	8.96E-03	3.81E-02
EP300	22	22q13.2	0.37	0.66	0.16	0.45	0.19	9.31E-03	3.91E-02
NUP50	22	22q13.31	0.22	0.32	0.74	0.50	0.22	1.04E-02	4.21E-02
YWHAB	20	20q13.1	0.12	0.61	0.37	0.41	0.18	1.05E-02	4.26E-02
RANGAP1	22	22q13	0.23	0.68	0.28	0.45	0.20	1.06E-02	4.28E-02
