# Supplementary material for: Expression alterations define unique molecular characteristics of spinal ependymomas
Source: Oncotarget. 2015 Mar 30;6(23):19780–91. doi: 10.18632/oncotarget.3715 (PMC4637320; doi:10.18632/oncotarget.3715)
Supplement: Supplementary file 7 [file oncotarget-06-19780-s007.pdf]

Supplementary Table 6. Description of gene expression microarray data sets used in the current analysis

	Intracranial			Spinal			
**Data set**	**N**	**Age**	**Gender**	**N**	**Age**	**Gender**	**Data source**
CBTRC	71	10.6 (0.4-61)	44/27	12	25.6 (1-59)	9/3	GSE21687
Toronto	85	8.1 (0.4-57)	48/36	15	35.6 (9-68)	8/6	GSE27279
Heidelberg	65	18.7 (1-63)	35/30	10	35.3 (20-55)	4/6	GSE27287
